# Patterns of facility and patient related factors to the orthopedic and trauma admissions at the Kenyatta National Hospital: A qualitative assessment

**DOI:** 10.1371/journal.pgph.0002323

**Published:** 2024-01-25

**Authors:** Maxwell Philip Omondi, Joseph Chege Mwangi, Fred Chuma Sitati, Herbert Onga’ngo

**Affiliations:** 1 Department of Surgery, Thematic Unit–Orthopedic Surgery, University of Nairobi, Nairobi, Kenya; 2 Department of Orthopedics, Kenyatta National Hospital, Nairobi, Kenya; Yale University School of Medicine, UNITED STATES

## Abstract

Inappropriate utilization of higher-level health facilities and ineffective management of the referral processes in resource-limited settings is increasingly becoming a concern in health care management in developing countries. This is characterized by self-referrals and frequent bypassing of nearest health facilities coupled with low formal referral mechanisms. This scenario lends itself to a situation where uncomplicated medical conditions are unnecessarily managed in a high-cost health facility. This situation compromises the ability and capacity of Kenyatta National Hospital (KNH) to function as a tertiary referral health facility as envisioned by Kenya Health Sector Referral Implementation Guidelines of 2014, Kenya 201 constitution and KNH legal statue of 1987. The study objective was to assess the patterns of facility and patient related factors to the orthopaedic and trauma admissions at the KNH. This was a descriptive qualitative study design. The study was conducted amongst the orthopaedic and trauma admission caseload for 2021. Data collection was done through a) data abstraction from 905 patients charts admitted during February to December 2021 and b) 10 (ten) semi-structured interviews with 10 major health facilities that refer to KNH to understand the reasons for referral to KNH. Quantitative data was analysed using Statistical Package for Social Science version 21.0 to calculate the frequency distribution. Qualitative data from the data abstraction and transcripts from the KIIs were analysed using NVivo version 12. The major facility and patient related factors to the orthopaedic and trauma admissions at KNH were inadequate human resource capacity and availability (42.7%), financial constraints (23.3%), inadequate Orthopaedic equipment’s and implants availability (20.0%) and inadequate health facility infrastructure (6.3%) while the major patient related factor was patient’s preference (23.4%). In conclusion, to decongest KNH requires that the lower-level health facilities need to be better equipped and resourced to handle essential orthopaedic and trauma care.

## Introduction

The types of admission need to reflect the level of health facility with tertiary facilities expected to handle more complex cases while lower-level health facilities should focus on the lesser-complicated cases due to the presence of limited equipment, physical infrastructure, and human resources. The reason for bypassing nearest health facilities seems to be multifactorial: patients’ perception of high quality of health care and resource availability at referral hospitals play a role [1, 2]. Tertiary hospitals in resource-limited countries treat not only patients referred but also act as the first level of care for the vast majority of patients [3].

One of the challenges in health care delivery in resource-limited settings is inappropriate utilization of tertiary health facilities that results in patients’ congestion in referral hospitals with simple conditions that can be effectively managed at the lower peripheral health facilities. The majority of these patients are self-referred, bypassing lower-level health facilities in the process [1, 4–6]. The net result is lesser medical conditions end up being managed in high-cost referral health facilities leading to overcrowding, long waiting times, and scarce staff time consumed by lesser medical conditions at the expense of complex medical conditions [3, 7].

Orthopaedic wards in KNH have consistently recorded the highest bed occupancy percent for the last couple of years. In 2018, 2019 and 2020 it recorded bed occupancy percent of 142.2%, 138.2% and 116.5% respectively against the KNH bed occupancy percent of 106.2%, 113.4% and 91.5% respectively [8]. The consequence of this is the low nurse-patient ratio of 1:10 that compromises not only the quality of nursing care given to patients but also compromises the ability of KNH to effectively perform its statutory obligations.

However, there is paucity of local data regarding the facility and patient related factors to the orthopaedic and trauma admissions in developing countries and particularly sub-Saharan Africa. Understanding the patterns of facility and patient related factors to the orthopaedic and trauma admissions at the KNH is important for patient planning and management and in a university facility, this is also critical in planning for quality training. The purpose of this study was to assess the patterns of facility and patient related factors to the orthopaedic and trauma admissions at the KNH.

## Materials and methods

### Ethical considerations

University of Nairobi/Kenyatta National Hospital Ethics and Research Committee granted ethical approval (ERC No: P852/10/2021). Administrative approval was also granted by KNH Medical Research Department and KNH Orthopaedics Department.

### Study design

This was descriptive qualitative study design.

### Study area

KNH Orthopaedic Wards. KNH is the national teaching and referral hospital based in Upperhill, Nairobi, the Capital city of Kenya.

### Data collection

Data collection was done through chart abstractions and Key Informant Interviews (KIIs).

**a) Chart abstractions.** *Study period*. Data was abstracted from patients’ charts covering 1^st^ February to 31^st^ December 2021. The data abstraction was done from 1^st^ January to 31^st^ March 2022 during the data collection period.

*Study population*. KNH Orthopaedic inpatient caseload admitted from 1^st^ February to 31^st^ December 2021.

*Sample size*. 905 chart reviews. A systematic sampling technique was used. This was part of a study that sought to determine the patterns of orthopaedic and trauma admissions at Kenyatta National Hospital.

*Pilot testing*. Pilot testing was done to assess the feasibility and appropriateness of the data abstraction tool to collect the data required to answer the study objective.

*Data collection procedures*. Data abstraction form was used to extract data from the patient files. Three (3) research assistants (2 females and 1 male) were recruited, trained on data abstraction process from the patients’ charts. The data collection was done at KNH Health Information Office. The principal investigator did data quality checks for all the abstracted data before and after data entry.

*Data management*, *analysis*. Quantitative data was analysed using Statistical Package for Social Sciences (SPSS) version 21.0 using frequencies. The qualitative data obtained from the chart abstractions were entered into excel spreadsheets and were subjected to thematic analysis using NVIVO 12 Pro. The research questions and responses from excel spreadsheets were reviewed for the identification of themes and patterns and a coding framework was developed. The coding framework and excel spreadsheet were then imported directly into NVIVO for coding. The coding process entailed going through the texts on the excel spreadsheet, dragging and dropping into correct nodes. Some codes were modified and new ones created as they came up while reviewing more spreadsheets entries. By analyzing the content of each code to check if they are correctly placed, those related to the objective of the study were noted down for summary report.

**b) Key Informant Interviews (KIIs).** *Study period*. Key Informant Interviews were done in January 2022 and the respondents shared their experiences and observations dating back from 1^st^ February to 31^st^ December 2021 period.

*Study population*. Key Orthopaedic and trauma staff from the 10 (ten) health facilities namely KNH, Mama Lucy Kibaki Hospital, Mbagathi Hospital, Machakos County Hospital, Mwingi County Hospital, Thika Level 5 Hospital, Ngong Sub- County Hospital, St Peters Orthopaedic Specialist Hospital, St Francis Community Hospital and Arthi River Shalom Community Hospital.

*Sample size*. 10 (ten) KIIs were conducted. These were purposively sampled. These were the health facilities that referred majority of patients to KNH during the study period.

*Pilot testing*. Pilot testing was done to assess the feasibility and appropriateness of the KII guide to collect the data required to answer the study objective.

*Data collection procedures*. The interviewees were medical officers and orthopaedic consultants responsible for patient orthopaedic and trauma referrals at the sampled health facilities. The interviews were interviewer-administered. The Principal Investigator (male) who is an orthopaedic surgeon conducted the interviews. The interviewees were briefed on the purpose of the study. They reviewed the participant information consent form prior to giving their written informed consent to participate in the study. The interviews were done at the sampled health facilities referral office/desk. KII interview guide was used to guide the Key Informant Interviews. The focus of the interviews was on common orthopaedic and trauma referrals to KNH, reasons for referrals to KNH, challenges, lessons learnt and their recommendations with a focus to improve orthopaedic and trauma care at their respective health facilities. The interviews lasted about 30 to 45 mins. Audio recordings were taken for each interview session and later transcribed into transcripts by one transcriber. The principal investigator went through the 10 audio recordings and the transcripts to ensure they were in verbatim and verified the accuracy of the transcription process.

### Data management, analysis

Audio recordings were transcribed and subjected to content analysis using NVIVO 12 Pro. The research questions and responses from transcripts were reviewed for the identification of themes and patterns and a coding framework was developed. The coding framework and transcripts were then imported directly into NVIVO for coding. The coding process entailed going through the texts on the transcripts, dragging and dropping into correct nodes. Some codes were modified and new ones created as they came up while reviewing more transcripts. By analyzing the content of each code to check if they are correctly placed, those related to the objective of the study were noted down for summary report.

### Study limitations

- Incomplete and missing records–this was addressed by making phone calls to patients to access the required data;
- All admissions were reviewed and therefore not necessarily focussing on admissions appropriate to KNH as a national referral health facility.
- Recall bias–the interviewees were to recall the reasons for referrals to KNH during the review period, February to December, 2022.

## Results

### Sample characteristics

The mean age was 33.8 years (SD 16.5) with range of 1–93 years. Majority 600 (66.3%) were between 25–64 years with those above 65 years being 40 (4.4%). About 453 (50.1%) were facility referrals while 452 (49.9%) were walk-ins. Majority of orthopaedic and traumatic admissions were through Accident and Emergency Department 707 (78.1%) followed by Corporate Outpatient Centre 135 (14.9%) and Orthopaedic Clinic 63 (7.0%). Of the 905 admissions 712 (78.7%) were emergency admissions while 188 (20.8%) were elective admissions (Table 1).

**Table 1:** Sample characteristics (N = 905).

Variable	Category	Frequency n (%)
**Age**	0–14 years	99 (10.9%)
	15–24 years	166 (18.3%)
	25–64 years	600 (66.3%)
	Above 65 years	40 (4.4%)
**Sex**	Female	198 (21.9%)
	Male	703 (77.7%)
	Missing	4
**Occupation**	Businessman/woman	112 (12.4%)
	Casual	405 (44.8%)
	Employed	135 (14.9%)
	Other	59 (6.5%)
	unemployed	183 (20.2%)
	Missing	11
**Education**	None	55 (6.1%)
	Pre-school	22 (2.4%)
	Primary	308 (34.0%)
	Secondary	317 (35.0%)
	Tertiary	182 (20.1%)
	Missing	21
**Mode of payment**	Cash	621 (68.6%)
	Insurance	281 (31.0%)
	Missing	3

Frequency distribution of key socio-demographic characteristics. Missing variables varied from 3 to 11 but they were excluded from proportional analysis for the variables concerned.

About 405 (44.8%) were casual workers, 183 (20.2%) unemployed. With regard to education level 308 (34.0%) had primary education, 317 (35.0%) had secondary education (Table 1).

The facility and patient related factors to the orthopaedic and trauma admissions at the KNH included human resource capacity and availability, health facility infrastructure, Orthopaedic equipment’s and implants availability, patient’s preference, unaccompanied patients and financial considerations among others (Table 2).

**Table 2:** Table showing the frequency distribution of the major facility and patient related factors to the orthopaedic and trauma admissions at the KNH.

Sample size (n)	Human resource capacity and availability	Patients’ preference	Financial constraints’	Orthopaedic equipment’s and Implant availability	Health facility Infrastructure
**905**	386 (42.7%)	212 (23.4%)	211 (23.3%)	181 (20.0%)	57 (6.3%)

This was a cumulative tally of the facility and patient related factors to the orthopaedic and trauma admissions at the KNH as a proportion of the sample size (n).

#### 1. Human resource capacity and availability

Human resource capacity and availability was the most frequently cited reason for bypassing the nearby health facilities at 42.7%. Chart reviews showed that most orthopaedic and trauma admissions to KNH were due to perceived or real inadequate human resource capacity and availability from the peripheral health facilities, both public and private health facilities. KNH is believed to have highly specialized personnel to manage diverse orthopaedic and associated conditions like plastic surgeons and neurosurgeons. Some health facilities with orthopaedic surgeons still refer polytrauma orthopaedic cases due to luck of other specialist personnel/care like neurosurgeons, Intensive Care Units or High Dependency Units care (Hospital 7 KII, Hospital 1 KII, Hospital 3 KII, Hospital 4 KII, Hospital 5 KII, Hospital 6 KII).

*“…. patient was taken to Kangundo hospital but there was not specialist to attend to the patient….” Walk-in patient from Machakos county*.

*“…. went to Thika level 5 hospital then they were transferred to KNH since they lacked a spine specialist….” Facility referral from Thika Level 5 Hospital*

There are those patients who were referred from the health facility due to the fact that there was no orthopaedic surgeon at the time of emergency at the referring health facility.

*“……there was no orthopaedic surgeon at that particular night so they decided to refer the patient to KNH to get assistance…*.*”* Facility referral from Mama Lucy Kibaki Hospital

*“…he needed an orthopaedic doctor for treatment of his condition*, *and Machakos did not have one at that moment…*.*”* Facility Referral from Machakos Level 5 Hospital

*“…I would say maybe especially at night*, *we refer Kenyatta because of the visiting consultants are not available at night…”* Hospital 6 KII

#### 2. Patients’ preference

Patients’ preference was the second most frequently cited reason for bypassing the nearby health facilities at 23.4%. Chart reviews from admission files revealed that some patients, their families, relatives and friends opted to go to KNH or be referred to KNH from other health facilities due to personal reasons and preferences. For some patients, either themselves or their relatives have been treated at KNH before and had good experience and outcome and ended by encouraging their loved ones and friends to seek services at KNH. This was triangulated with KII findings that revealed orthopaedic patients ask for referrals or decide on their own volition to present themselves to KNH for treatment based on their previous experience and perception of better quality of care (Hospital 4 KII, Hospital 3 KII, Hospital 2 KII).

*“…Patient prefers KNH since family members have been treated there and fully recovered”, Walk-in from Westlands*

*“…*. *his family wanted him to come closer to where they are since the father is a doctor at KNH…*.*” Facility Referral*, *Tenri Embu Children Hospital*.

#### 3. Financial constraints

Financial constraints were the third most frequently cited reason for bypassing the nearby health facilities at 23.3%. According to significant number of orthopaedic admissions, the referral was done because of financial constraints since they could ill afford the private health facilities, they were in. Most of the private health facilities were not accepting the government National Health Insurance Fund cover for orthopaedic and trauma admissions. This was triangulated with KII findings that revealed the most common reason for referral from private health facilities to KNH was financial constraints. Majority of admissions to KNH are from low socio-economic status with no insurance cover and therefore could not afford to meet the cost of private health facilities (Hospital 1 KII, Hospital 2 KII, Hospital 4 KII, Hospital 5 KII).

*“…she couldn’t afford to pay at Nairobi South hospital so her doctor requested for the surgery to be done at KNH since the doctor was also from KNH*…*”* Facility Referral from Nairobi South Hospital

*“…Metropolitan Hospital was too expensive for the patient thus opting for KNH…*.*”* Facility Referral from Metropolitan Hospital

*“…we mainly refer due to cost…*.*”* Hospital 9 KII

#### 4. Orthopaedic equipment and implants availability

Orthopaedic equipment and implants availability was the fourth most frequently cited reason for bypassing the nearby health facilities at 20.0%. Chart reviews showed that most health facilities and patients believed KNH had better orthopaedic equipment’s and implants to manage diverse and complicated orthopaedic and trauma conditions for example Computed Tomography (CT) scans, Magnetic Resonance Imaging (MRI) and X-rays for diagnosis and patient management compared to the peripheral health facilities. These were triangulated with findings from KIIs done with the main referring public health facilities that revealed most of these public hospitals lack imaging equipment’s like X-rays, CT scan and MRI and for those who have, the machines are sometimes faulty and in a sorry state. Patients then end up being referred to KNH for imaging studies (Hospital 1 KII, Hospital 3 KII, Hospital 2 KII, Hospital 4 KII, Hospital 6 KII, Hospital 5 KII)

*“…Kenyatta is better equipped*…*”* Walk-in patient

*“…Sinai Hospital lacked machines to do X-rays…” Facility Referral from Sinai Hospital, Rongai*

*“Mama Lucy Hospital lacked the metals that were supposed to be put on the leg*… ..*”* Facility Referral from Mama Lucy Kibaki Hospital

In addition, some health facilities had non-functioning imaging machines and so they had to refer them to KNH where they believed machines were available and in good working condition.

*“…X-rays machines were not working at that particular time*…*”* Facility Referral from Mbagathi DH

*“…*. *machines were not working at Mama Lucy”* Facility Referral from Mama Lucy Kibaki Hospital

Orthopaedic implants and sets are not available in most of these public and private peripheral health facilities. Most of these implants are outsourced and this requires that have to pay for them in advance before they implant can be procured. Those patients with no insurance cover, low socio-economic status and therefore cannot afford to pay for the implants, end up being referred to KNH (Hospital 10 KII, Hospital 8 KII).

#### 5. Health facility infrastructure

Health facility infrastructure was one of the less frequently cited reason for bypassing the nearby health facilities at 6.3%. From chart reviews, a number of patients were referred to KNH because of the unavailability of infrastructure and better facilities to handle and perform orthopaedic operations. KNH is considered to have superior and capacious infrastructure to handle complex orthopaedic complications.

*“…*. *KNH had better structure and facilities to help in his condition…”* Facility referral from Mama Lucy Kibaki Hospital

*“…there was no bed space in Mama Lucy…” Facility Referral from Mama Lucy Kibaki Hospital*

This was triangulated with KII findings that revealed there was no enough infrastructure in some public hospitals with some lacking operation rooms while others health facilities have to share the theatre space making it hard for orthopaedic procedures to be done when required. Some public hospitals have no ICU or have few ICU beds. Some health facilities simply have inadequate bed capacity for orthopaedic admissions and have to refer to KNH when their capacity gets exceeded (Hospital 1 KII, Hospital 8 KII, Hospital 2 KII, Hospital 7 KII, Hospital 4 KII).

*“…because we have two theatres*, *but there is one specifically for maternity*. *This other one we use for Obs and gynae*, *general surgery*, *ENT*. *So*, *we have one day each for each department*…*”* Hospital 4 KII

#### 6. Quality of health services

Quality of health services also came up as one of the factors affecting orthopaedic and trauma admission to KNH. Chart reviews showed some patients preferred to be referred to KNH because of the poor health care and services they got from other peripheral health facilities like nurses being rude, unprofessional conduct. Some of these patients had received unfavourable commendations about peripheral health facilities with regard to the poor quality of health care provision.

*“…i opted for KNH after getting advice from several people who had attended Mama Lucy Hospital…”* Walk in from Mathare North

*“…nurses at Mama Lucy are rude unlike Kenyatta where they are friendly…”* Walk-in from Embakasi West

#### 7. Proximity to KNH

Chart reviews showed that some patients were either brought or came to KNH because it was the nearest facility from their residence or from the scene of the accident. Triangulated findings from KII revealed that referrals to KNH was convenient because it was near. The patients preferred being referred to KNH for convenience’s sake (Hospital 1 KII, Hospital 7 KII).

*“…was rushed by an ambulance to KNH as it was the nearest from the scene”* Walk-in from Mavoko

*“…area of accident was closer to KNH than any other health facility…”* Walk-in from Ruiru, Kiambu county

#### 8. Unaccompanied

From the data abstractions, some admissions were unconscious and just found themselves at KNH when they woke up not knowing how they got there. For others, it was the ambulance, good Samaritans or police officers that brought them direct to KNH from the accident scene. Some paramedics thought it wise to bring them to KNH given the perceived severity of their injuries.

*“…the people who were present at the area of accident opted for KNH”*, Walk-in from Embakasi South

*“…he was unconscious and was picked by an ambulance and decided to bring to KNH…”* Walk-in from Embakasi Central

*“…the patient was brought in to KNH by police…*.” Walk-in from Starehe

## Discussions

### 1. Human resource capacity and availability

Human resource for health is a critical pillar in WHO health system strengthening and a key component in access to health care. Quantity and quality of human resource for health determines the quality of care received. This study showed that most of the admissions to KNH were due to need for specialized orthopaedic and trauma surgeons to handle orthopaedic (spine and pelvic injuries) and other injuries including polytrauma patients. This compares with studies done on reasons for inter-hospital transfer of trauma patients that revealed that severely injured trauma patients and those with pelvic injuries are likely to be transferred to advanced trauma centres for management and for better outcomes [9–12]. The advanced centres are presumed to have well trained personnel with relevant experience to handle complicated cases. This reinforces studies that revealed limited staff capacity at the peripheral facilities necessitates surgical referrals in low- and middle-income countries [13–18]. Most of these polytrauma patients require multidisciplinary team for effective management and this is usually not available at the peripheral facilities.

### 2. Patients’ preferences

Patients’ preferences refer to patients’ values, beliefs, expectations and health goals that influences their health choices and health facility choices as well. This study did reveal the role of family, friends and the society at large in health seeking behaviour of orthopaedic admissions.

The study did show that some patients, their families, relatives and friends opted to go to KNH or be referred to KNH from other health facilities due to personal reasons, and preferences. For some patients, either themselves or their relatives have been treated at KNH before and had good experience and outcome and ended up encouraging their loved ones and friends to seek services at KNH. This compares favourably with a study done in Israel indicated that about 33% of patients seek second opinion on recommendation of a relative and/or friend [18, 19]. However, this contradicts a review done in Northern Tanzania that showed patients preference accounts for about 1% of surgical patient’s referral to a Tertiary health facility [18]. A systematic review of factors patients consider in choice of a surgeon reveal that hospital reputation, rather than surgeon’s reputation, was of primary importance and most patients relied on word-on-mouth and physician preference to decide the hospital of choice [20].

### 3. Financial constraints

Personal finance is key factor in the choice of a hospital with patients of high socio-economic status and with insurance cover opting for high -end private health facilities while patients of low-socio-economic status and mostly with no insurance cover opting to seek services in government health facilities.

This study did show that for a significant number of admissions, the referral was done because of financial constraints since they could ill afford the private health facilities, they were in. In addition, most of the private health facilities would require additional financial top ups in addition to the NHIF cover while others would outrightly reject the NHIF cover due to its low financial allocations for orthopaedic procedures. A number of private orthopaedic patients were advised by their doctors to seek care at KNH because of their relative affordability and ability to make use of the NHIF cover to sort their medical bills, given that most of the admissions are not well-endowed financially.

### 4. Orthopaedic equipment and implants availability

Equipments, supplies are another WHO building block for effective health service delivery. Orthopaedic equipment’s, sets and implants are generally costly and require significant capital outlays. Availability of Orthopaedic equipment’s, sets and implants are key to management of orthopaedic patients. Given the level and the nature of health care financing in Kenya, most health facilities—both government and private—are not able to acquire and maintain these equipment’s.

KNH being a premier referral facility in the country is believed to be well resourced and therefore best equipped in this regard including CT scans, MRI and X-rays to manage diverse and complicated orthopaedic conditions compared to the peripheral health facilities. This compares with a qualitative study done in Uganda that revealed unavailability, non-functional or intermittent functioning of imaging facilities like CT scan, MRI that hindered surgical service provision [14, 18]. This also compares with a study done in Muhimbili National Hospital in Tanzania that revealed lack of equipment as the main reason for referral to the Tertiary facility [21]. Similar studies done in Kenya, Uganda, Tanzania, Rwanda and Ghana revealed infrastructure gap at the peripheral health facilities necessitated referrals to tertiary health facilities [22].

### 5. Health infrastructure

Health Infrastructure is one of the WHO pillars of health system strengthening and provides the structural framework for provision of health care. It refers to availability of health amenities, adequate ward, theatre rooms, physiotherapy, occupational therapy spaces for Orthopaedic and Trauma and Rehabilitation service provisions. Poor infrastructure means compromised health service delivery.

Orthopaedic patients were also referred or self-referred to KNH because of the unavailability of bed space and poor facilities to handle and perform orthopaedic operations. This compares with a prospective study done in Aga Khan University Hospital in Kenya that revealed bed availability was one of the commonest reasons cited for referral [23]. Similar studies in Uganda, Rwanda have shown inadequate health infrastructure with limited ward and theatre capacity hinders provision and access to surgical services [14–16, 18]. The operating rooms are shared by other surgical specialities and most often overburdened by urgent obstetric cases that often lead to planned orthopaedic operations delayed or postponed due to the urgent obstetric procedures. This effectively means reduced operative capacity for orthopaedic procedures. Some of these public health facilities in addition to limited operative capacities also lacked ICU or HDU capacities to handle polytrauma patients with neurosurgical complications.

#### 6. Quality of health services

Quality of health service delivery is one of the WHO pillars of health care strengthening and it requires provision of quality health care. This study did show that some patients preferred to be referred or self-referred to KNH because of either perceived or real poor health care services they got from other peripheral health facilities like the nurses being rude or unprofessional conduct. The unfavourable commendations about peripheral health facilities with regard to the poor quality of health care provision influenced their decision to seek services at KNH. This compares favourably with a number of studies have shown that quality of service influences the choice of a health facility with hospitals with high quality of care being hospitals of choice [24, 25].

### 7. Proximity to KNH

Geographical distance is a key consideration for choice of a hospital. Long distance travel has financial implications but also it is not convenient for friends, relatives and family of the patients who seek to visit and take care of them seek ones.

A number of admissions were either brought or came to KNH because it was the nearest facility from their residence or from the scene of the accident; the paramedics decided to rush them to KNH being the nearest government hospital that’s is affordable. For some, they had relatives in Nairobi and so it was easier for the relatives to monitor them. This compares favourably with a systematic review of factors patients consider in choice of health provider that revealed hospital distance was of primary importance in choosing facility of choice [20, 24].

### 8. Unaccompanied

Unaccompanied means that these are admissions that were brought in by good Samaritans or police officers and not relatives as unconscious and not much was known about them at the time of admission. They were victims of road traffic accidents, assaults or mentally ill patients that sustained orthopaedic related injuries. The polices officers, the good Samaritans are normally the first responders at the scene of accident and most of them make the decision to refer the patients to KNH either due to financial considerations given KNH is a government facility or due to proximity to KNH given that majority of the RTAs occur within Nairobi County. The unaccompanied and unconscious patients at the scene of accident are deemed to have serious injuries. This compares with studies that showed Police officers are usually the first responders at the scene of accident [26, 27]. Similarly a study done on post-crash emergency care in India showed good Samaritans and police officers referred about 90% post-crash victims to government hospitals as the first contact health facility [28].

## Conclusions

The patient and facility related factors to the orthopaedic and trauma admissions at the KNH included inadequate human resource capacity and availability, inadequate health facility infrastructure, inadequate Orthopaedic equipment’s and implants availability, patient’s preference, unaccompanied patients and financial constraints.

## Recommendations

### 6.2.1 Recommendations to County Government

1. Ensure availability of accessible and high-quality orthopedic and trauma health services at the lower tier health facilities;
2. Provide regular supportive supervision and capacity building of county health facilities on the referral system;
3. Allocate more resources to human resource for health for recruitment of more orthopaedic and trauma surgeons and also fund training for more specialized orthopaedic speciality including pelvic and spine specialities;
4. Improve the health infrastructure and operative capacity of peripheral facilities to be able to handle orthopaedic and trauma cases;
5. The high cost of Orthopaedic sets and implants is an impediment to orthopaedic care. The government to consider subsiding the cost of orthopaedic equipment’s, sets and implants to reduce the overall cost of orthopaedic procedures and make it more affordable and accessible;

#### 6.2.2 Recommendations to KNH

1. Regular training of staff at the referral coordination Unit to ensure adherence to the referral guidelines;
2. Educate and sensitize the public, the police force as to the role of KNH as a premier National Teaching and Referral facility that is mandated to manage complex referrals and should not be the first point of contact for patients seeking orthopaedic care;
3. Have a written standard operating procedure on orthopaedic and trauma referrals to KNH. This referral guideline should be reviewed and updated as necessary;
4. Consider providing outreaches/mentorship to high volume peripheral facilities on a regular basis in management of pelvic and spine injuries as part of mentorship and capacity building;
5. Offer fellowships and short-courses on targeted themes on orthopaedic and trauma care namely spine, pelvic/acetabular management, polytrauma management to improve capacity for the referring health facilities;

#### 6.2.3 Recommendations to health facilities

1. Human resource capacity building for Orthopaedic surgeons and fund subspeciality trainings in orthopaedics to handle complex orthopaedic cases like spine, pelvic and hand injuries;
2. Need to address the culture of Ministry of Health staff with regards to unavailability/ absenteeism at work to avoid unnecessary referrals of patients and also for timely management of orthopaedic patients;
3. Improvement in health infrastructure and this includes increasing the operative capacity of the major health facilities bed capacity, imaging equipment’s like CT scan, MRI, X-rays to reduce on unnecessary referrals;
4. Strengthen the maintenance department to ensure that facilities with imaging equipment’s, theatre equipment’s are serviced on a timely manner to minimize on frequent breakages and non-functioning equipment’s that impede effective and timely service provision;
5. Fund the procurement of basic Orthopaedic equipment’s, sets and implants for basic orthopaedic procedures that can be handled by resident orthopaedic surgeons;

## Supporting information

S1 Checklist STROBE statement—checklist of items that should be included in reports of *cross-sectional studies*. (DOC)

S2 Checklist Consolidated criteria for reporting qualitative studies (COREQ): 32-item checklist. (DOCX)

S1 Data Dataset SPSS. (SAV)

S2 Data Spreadsheet database. (XLSX)

S1 Text Health facility keys. (DOCX)

S1 File Key informant interviews TRANSCRIPTS. (ZIP)
