# Supplementary material for: Patterns of facility and patient related factors to the orthopedic and trauma admissions at the Kenyatta National Hospital: A qualitative assessment
Source: PLOS Glob Public Health. 2024 Jan 25;4(1):e0002323. doi: 10.1371/journal.pgph.0002323 (PMC10810445; doi:10.1371/journal.pgph.0002323)
Supplement: S2 Checklist — (DOCX) [file pgph.0002323.s002.docx]

**Manuscript:** Patterns of Facility and Patient Related Factors in Orthopedic and Trauma Admissions at Kenyatta National Hospital, Kenya: A Qualitative Assessment

**Consolidated criteria for reporting qualitative studies (COREQ): 32-item checklist**

Developed from:

Tong A, Sainsbury P, Craig J. Consolidated criteria for reporting qualitative research (COREQ): a 32-item checklist for interviews and focus groups. *International Journal for Quality in Health Care*. 2007. Volume 19, Number 6: pp. 349 – 357

| **No. Item** | **Guide questions/description** | **Reported on Page #** |
| --- | --- | --- |
| **Domain 1: Research team and reﬂexivity** |  |  |
| *Personal Characteristics* |  |  |
| 1. Inter viewer/facilitator | Which author/s conducted the interview or focus group? | The 3 Research assistants abstracted the data from the patients charts. Page 5(Line 100 and 101) The principal investigator conducted the KIIs Page 6 (Line 132 and 133) |
| 2. Credentials | What were the researcher’s credentials? E.g. PhD, MD | MD, PhD |
| 3. Occupation | What was their occupation at the time of the study? | The key informant interviewees were medical officers and orthopedic consultants at the sampled health facilities. Page 6 (Line 130 - 132) |
| 4. Gender | Was the researcher male or female? | Page 5  (Line 100) and page 6 (Line 132 – 133) |
| 5. Experience and training | What experience or training did the researcher have? | Page 5 (Line 100 - 101)  Page 6 (Line 132 - 133) |
| *Relationship with participants* |  |  |
| 6. Relationship established | Was a relationship established prior to study commencement? | **Yes**  The 10 health facilities sampled had been referring patients to KNH before the study comemncement. Page 9 (Line 125 – 127) |
| 7. Participant knowledge of the interviewer | What did the participants know about the researcher? e.g., personal goals, reasons for doing the research | Participants were briefed on the purpose of the study and understood it. Ethical had granted, participants reviewed the participant information documentation prior to giving their written informed consent to be involved. Pages 6 and 7 (Line 133 - 135) |
| 8. Interviewer characteristics | What characteristics were reported about the inter viewer/facilitator? e.g., Bias, assumptions, reasons and interests in the research topic | Page 6 (Line 132 – 133) |

| **Domain 2: study design** |  |  |
| --- | --- | --- |
| *Theoretical framework* |  |  |
| 9. Methodological orientation and Theory | What methodological orientation was stated to underpin the study? e.g., grounded theory, discourse analysis, ethnography, phenomenology, content analysis | Grounded theory. See introduction Pages 3 -4 |
| *Participant selection* |  |  |
| 10. Sampling | How were participants selected? e.g., purposive, convenience, consecutive, snowball | 1. Systematic sampling - Page 5 (Line 95 -97) 2. Purposive sampling. Page 6 (Line 125 -127) |
| 11. Method of approach | How were participants approached? e.g., face-to-face, telephone, mail, email | Data abstraction from patients’ charts. Page 5 (Line 100 -104) and Face to face for KIIs page 6 (Line 131 -133) |
| 12. Sample size | How many participants were in the study? | Page 5 (Line 95 -97)  Page 6 (Line 125 -127) |
| 13. non-participation | How many people refused to participate or dropped out? Reasons? | **None** |
| *Setting* |  |  |
| 14. Setting of data collection | Where was the data collected? e.g., home, clinic, workplace | At the hospital.  Page 5 (Line 102 – 103)  Page 6 (Line 132 - 133) |
| 15. Presence of non-participants | Was anyone else present besides the participants and researchers? | **No** |
| 16. Description of sample | What are the important characteristics of the sample? e.g., demographic data, date | Page 9 Table 1 |
| *Data collection* |  |  |
| 17. Interview guide | Were questions, prompts, guides provided by the authors? Was it pilot tested? | Page 5 (Line 98 -99)  Page 7 (Line 137 - 138)  Page 6 (Line 129 – 130) |
| 18. Repeat interviews | Were repeat inter views carried out? If yes, how many? | **No** |
| 19. Audio/visual recording | Did the research use audio or visual recording to collect the data? | **Yes**  Page 7 (Line 142) |
| 20. Field notes | Were ﬁeld notes made during and/or after the interview or focus group? | Page 7 (Line 142 - 145) |
| 21. Duration | What was the duration of the inter views or focus group? | Page 7 (Line 141 - 142) |
| 22. Data saturation | Was data saturation discussed? | **No** |
| 23. Transcripts returned | Were transcripts returned to participants for comment and/or correction? | **No**  Since they were audio-recorded and verification could be done without reference to participants |
| **Domain 3: analysis and ﬁndings** |  |  |
| *Data analysis* |  |  |
| 24. Number of data coders | How many data coders coded the data? | **One (The author)** |
| 25. Description of the coding tree | Did authors provide a description of the coding tree? | Page 7 (Line 147 - 155) |
| 26. Derivation of themes | Were themes identiﬁed in advance or derived from the data? | Page 7 (Line 147 - 155)  Themes were derived from the data |
| 27. Software | What software, if applicable, was used to manage the data? | **Microsoft Word and Excel spreadsheets** |
| 28. Participant checking | Did participants provide feedback on the ﬁndings? | **No** |
| *Reporting* |  |  |
| 29. Quotations presented | Were participant quotations presented to illustrate the themes/ﬁndings? Was each quotation identiﬁed? e.g., participant number | **Yes.**  Page 10 - 17 |
| 30. Data and ﬁndings consistent | Was there consistency between the data presented and the ﬁndings? | **Yes**  Data triangulation was done. Page 7 (Line155 -156) |
| 31. Clarity of major themes | Were major themes clearly presented in the ﬁndings? | **Yes**  Page 10 table 2 |
| 32. Clarity of minor themes | Is there a description of diverse cases or discussion of minor themes? | **Yes**  Page 10 table 2 |
