# Supplementary material for: Patterns of facility and patient related factors to the orthopedic and trauma admissions at the Kenyatta National Hospital: A qualitative assessment
Source: PLOS Glob Public Health. 2024 Jan 25;4(1):e0002323. doi: 10.1371/journal.pgph.0002323 (PMC10810445; doi:10.1371/journal.pgph.0002323)
Supplement: S1 Text — (DOCX) [file pgph.0002323.s005.docx]

HEALTH FACILITY KEYS

| HEALTH FACILITY | KEYS |
| --- | --- |
| Kenyatta National Hospital | 1 |
| Mbagathi District Hospital | 2 |
| Mama Lucy Kibaki Hospital | 3 |
| Mwingi County Referral Hospital | 4 |
| Arthi River Shallom Hospital | 5 |
| St Francis Community Hospital | 6 |
| Ngong Sub- County Hospital | 7 |
| Machakos Level 5 Hospital | 8 |
| St Peters Orthopedic Hospital | 9 |
| Thika Level 5 Hospital | 10 |
