# Supplementary material for: Patterns of facility and patient related factors to the orthopedic and trauma admissions at the Kenyatta National Hospital: A qualitative assessment
Source: PLOS Glob Public Health. 2024 Jan 25;4(1):e0002323. doi: 10.1371/journal.pgph.0002323 (PMC10810445; doi:10.1371/journal.pgph.0002323)
Supplement: S1 File — (ZIP) [file pgph.0002323.s006.zip › KII TRANSCRIPTS/ARTHI RIVER SHALLOM HOSPITAL (1).docx]

| **FACILITY** | **ARTHI RIVER SHALLOM HOSPITAL** |
| --- | --- |
| **INTERVIEWER** | **Dr Maxwell Omondi** |
| **TRANSCRIBER** | **Dora Bloch** |

**I: My name is Dr Maxwell Omondi, I am an orthopaedic registrar, and you are Dr Raphael?**

R; Yes, Dr Raphael., head of head of clinical.

**I: I’m an orthopaedic registrar Kenyatta, my third year and we wanted to basically understand the issue around orthopaedic referrals from Shallom a private facility to Kenyatta. Because Shallom is one of the facilities that; top 10 facilities that actually refer patients to Kenyatta. We have gone through others that have done so and I think I’m left with Machakos which I’m waiting to be given the date to know when I can go to Machakos to do this and Kenyatta. Those are the two facilities left.**

R: Okay.

**I: We took the top ten facilities for last year; year 2021 that referred to Kenyatta orthopaedic patients that end up in admission. So, one thing I wanted to basically understand, is, where do you refer most of your orthopaedic patients?**

R: Orthopaedic patients, we either refer to Machakos or Kenyatta.

**I: Refer to…**

R: Machakos…

**I: Or Machakos.**

R: Mmhh.

**I: But majority where do you refer them mostly?**

R: Kenyatta.

**I: Kenyatta?**

R: Mmhh.

**I: Kenyatta. And what are the patterns; what are the patterns, what are the conditions, what are the type of injuries orthopaedic cases that you refer?**

R: Let me say multi-trauma patients who need maybe ICU care.

**I; Multi-trauma patients.**

R: Yes, then there is all those knuckle femur fracture; actually here we don’t do. And then things like knee replacement, we don’t do, and foot surgery we don’t do. So those cases we refer to Kenyatta.

**I: Foot surgery you don’t do?**

R: Yes.

**I: Hip?**

R: Hip we don’t do.

**I: Even…**

R: Hip, knee, and foot we don’t do.

**I: Those are the cases that you actually refer?**

R: Yes.

**I: What of the long bones femur?**

R: Femur we do, tibia we do…

**I: There is a consultant here?**

R: There is a consultant.

**I: Orthopaedic?**

R: Orthopaedic yes.

**I: Resident?**

R: Yes, I can say resident.

**I: There is one?**

R: There is one. We have Dr Ayuka, also we have Dr Zembi. Dr Zembi is a senior registrar, she has already finished.

**I: She’s a [inaudible 02: 49]?**

R: No, she’s a UON MD

**I: Oh, the orthopaedic?**

R: Mmhh.

**I: Called who?**

R: Dr Zembi.

**I: I don’t think I know her.**

R: She has finished her training and currently she is working with Dr Ayuka for those two years she was getting a registration as an orthopaedic surgeon.

**I: Oh.**

R: Yeah, those are the two.

**I: So those are the cases they cover? Most which cases are they, which ones do you say are the majority?**

R: Majority, hip-knees…No Hip.

**I; Majority are…**

R: Hip.

**I: Hip replacement?**

R: Yes, hip replacement.

**I: These are; we are talking of referrals to Kenyatta?**

R: Kenyatta yes.

**I: Kenyatta particularly.**

R: Yes.

**I: If you look at it better, are you aware of the guideline that we had; KNH came up with a referral guideline from 1^st^ of July last year.**

R: 1^ST^ OF July last year?

**I: Mmhh.**

R: What I know about KNH, is whenever we get a patient we want to refer, first we call the emergency department, talk to them about the patient. If it’s a patient who likely needs an ICU, they will connect us to ICU we talk to the guys in ICU. If there is a bed, then they will accept the patient. They will admit the patient. If not, then the patient will be admitted. Or else if it is just a stable patient, the surgery can’t be done here, we just talk to guys in casualty and they connect us to the ortho department because there are registrars who…

**I: On call?**

R: Yes, on call let me say that. We then talk to them, if it is possible, we can send a patient. Most of the time we send patients after calling Kenyatta. The…

**I: Even for the likes of femur fractures?**

R: Yes they take.

**I: You don’t have an issue where you will call and they say no?**

R: Sometimes they are like “Can you send the patient to Machakos”, but most of the time after calling Machakos, we call KNH because Machakos will say they the doc is not around; the ones doing those procedures, it’s better you send to KNH. And you see Kenyatta has so many consultants, whenever you sent a patient for HIP replacement or DHS, that one will be done in Kenyatta other than Machakos; for a long stay of the patient in the ward.

**I: Are you aware of the referral guideline for KNH came up with?>**

R: That one no.

**I: They didn’t tell you?**

R: They didn’t.

**I: They didn’t call you like…**

R: There is this process of referral in KNH?

**I: yeah.**

R: No.

**I: They have not…**

R: But what we know is you call before the patient be referred. You call and when they accept, you send the patient.

**I: What is the profile; what kind of patients do you end up referring? Not in terms of the nature of orthopaedic injury or orthopaedic cases, but in terms of the patient characteristics; age, sex, status, religion. Is there any pattern that you’ve noted that this are the kind that you end up referring?**

R: Kenyatta, I think most are a bit young, let me say in between 30-45.

**I: 45 years?**

R: Mmhh, because most of them get those bad injuries maybe an RTA, and then you send them to Kenyatta.

**I: Which kind of RTA?**

R: It is just…

**I: Is it motorcycle, is it pedestrian?**

R: Motorcycle most of the time.

**I: The motorcycle people?**

R: The motorcycle people and others who drive.

**I: Just the normal…**

R; Just the normal, yes.

**I: Vehicle accident.**

R: Vehicle accidents yes.

**I: But majority you say are which ones?**

R: Motorcycle; majority is motorcycle.

**I: Motorcycle.**

R: Mmhh.

**I: Sex?**

R: Mostly they are male.

**I: Mostly are male?’**

R: Yeah.

**I: Female?**

R: Female are few; they are very few.

**I: They are very few?**

R: Yes, very few

**I: And children?**

R: Children, very rare.

**I: Rare children?**

R: Mmhh.

**I: In terms of economic status, are they people who have insurance, they don’t have insurance; what is…**

R: Most of them have NHIF, few of them cash…

**I; Most of the ones you refer have NHIF?**

R: Yes, have NHIF.

**I: Few are cash payers?**

R: Yes, cash. Some people have insurance but they don’t tell you that they have insurance. If they have insurance, then they will ask for another hospital; another private hospital where they can be taken cared for very first. But most of the time…

**I: They go to KNH.**

R: They go to KNH.

**I: But they still have NHIF some of them cash**

R: Yes, and some cash, yes. But they go mostly to KNH; like 90 % go to KNH.

**I; 90% GO TO KNH. What would you say is the catchment population of these orthopaedic cases; where are they coming from mostly?**

R: Mostly it’s from this Athi River area and Syokimau.

**I: What are the common orthopaedic trauma cases… I think this one you have talked about.**

R: Mmhh.

**I: The common orthopaedic cases, we talked about politrauma who need ICU care.**

R: ICU care, those ones of hip.

**I: Hip.**

R: Fractures and then…

**I: Hip fractures.**

R: Knee surgeries.

**I: mostly are these ones?**

R: Mmhh.

**I: And you said most of them are actually male, very few are children that you end up referring. What are the common; factors that are associated with the referrals? What grounds do you end up…The basis of referring patients to Kenyatta?**

R: The basis for…

**I: The reason.**

R: The reason we say is infrastructure.

**I: What do you mean by infrastructure?**

R: Okay, is it really on infrastructure or let me say human resource.

**I: Human resource?**

R: Yes, human resource.

**I: You said you have 2 orthopaedic surgeons.**

R: orthopaedic surgeons.

**I: How…**

R: But whenever we call them they that surgery they can’t do it in Shallom. That’s what I was saying infrastructure.

**I: What is missing?**

R: Back then it was a SIAM, we didn’t have a SIAM but it was bought.

**I: When was it bought?**

R: Like 4 months ago.

**I: This year?**

R: This year basically.

**I: But we are checking for last year, so it means last year there were no SIAM?**

R: No, there was no SIAM, but even if we have SIAM, there is no hip surgery that…Until now there is no hip surgery that was done. Even if we have SIAM. So, I don’t know if that is human resource or it is infrastructure because…

**I: The two surgeons are not comfortable…**

R: It seems like they are not comfortable. It seems they are nor comfortable because I remember we have done a hip replacement with Dr, Sang.

**I; Here?**

R: Yes, a hip replacement with Dr Sang and it went well.

**I: So it is all about expertise?**

R: Yeah.

**I: So, there is lack of expertise to do these kind of surgeries?**

R: Yes.

**I: What are the reasons; you say would be the reasons for that one?**

R: In terms of infrastructure, back then we didn’t have an ICU room, now we have. Set up one, it depends; Kenyatta is a public and Shallom is private, so the cost maybe in private and public, it does make the difference. Because when you start with us it will be 200, but Kenyatta will be a bit lower in terms of the deposit and the ICU care itself. Most of the time if we see a patient can’t afford our ICU, then we refer to Kenyatta.

**I; So financial constraints?**

R: And mostly…Maybe, yeah financial constraints sometimes.

**I; To afford the ICU care.**

R: Yes.

**I: Anything else you think could be the reason for referral?**

R: No…. Equipment and implant availability, yeah we have.

**I: You have all the implants?**

R: Yes we have,

**I: You can do all the femur, DHS.**

R: DHS implants are there.

**I: You have in store or you get via your sources?**

R: We have sources.

**I: So you have got the companies that…**

R: Yes.

**I: The implant is not an issue?**

R: The implant is not an issue.

**I: It is the cost?**

R: The cost

**I: Patient can’t afford.**

R: Patient can’t afford.

**I: Do you have a situation whereby you refer because a patient can’t afford a DHS for example? Or a [inaudible 13:05] femur [inaudible 13:07]**

R: For us we prefer sending the patient to Kenyatta; I don’t know but, Kenyatta at the same time is a school, so whenever you send a patient, there is a cost and care. Sending a patient to another private hospital, maybe the patient will be getting the same issue; maybe cost, maybe implant. But in Kenyatta, us we believe there is everything.

**I; The main issue that we talk about these referral, is actually expertise.**

R: Expertise, yes.

**I: The orthopaedic surgeons that are here are not comfortable with doing these kind of cases like the [inaudible 13:47] femur?**

R: Yes, maybe; I feel like, because we have done one or even two new replacement with Dr Sang. I told you it went...

**I: Well.**

R: Well and the patient was discharged. Then they say “Those kind of cases we can’t do in Shallom”. We were like “These cases were done in Shallom, and then now…” you start asking yourself at the same time you don’t judge.

**I: Are issue about patient preference…Do you have cases whereby patients want to go to Kenyatta because it is just their preference to go to Kenyatta?**

R: Yes, but few patients. They are there, but few.

**I: But few?**

R: Yeah.

**I: The few who want to go there, why do they recommend; what are the reasons for them?**

R: The reasons for them, sometimes they extrapolate condition of the patient whereby maybe a patient has pain and that is normal for a fracture when you start managing and then the pain…It’s kind of managed “I feel our patient will be well taken care of at Kenyatta, please refer to Kenyatta”.

**I; Who?**

R: Themselves, they believe in Kenyatta it will well taken care than Shallom. Also, maybe they are from Nairobi and then travelling everyday Nairobi to Shallom Athi River, they can’t make. So they prefer “Send us to Kenyatta so at least…”

**I: Proximity?**

**R: Proximity.**

I: But why did they come here in the first place if they are saying proximity.

R: Those patients whenever they get an accident, the police bring them like the first point of care. They come here, then we treat them then the family comes. At that time then you know this patient is from Nairobi because they are asking for a referral to go to Kenyatta because it’s a near hospital.

**I: It will be convenient for them to visit…**

R: Yes

**I: Do you have any recommendations; programmatic, what do you recommend for these referrals, how do we streamline it, any challenges you have experienced?**

R: The challenges about Kenyatta?

**I: About the referrals of orthopaedic cases to Kenyatta.**

R: One, if Kenyatta can put like every department has its own line whereby when you want to call an ortho department, you just call straight. Even if the case will pass through casualty, you call straight to the orthopaedic department, the person will call to casualty that there is a patient who is coming, please receive that patient I’ll be to see the patient, and then meet the patient. We end up having problems with people in Kenyatta whereby we call, no one is picking. Or we call you tell them about the cases and you state the patient very well; you tell them this kind of surgery we don’t do it at Shallom, and at the end of the day they tell you “We don’t see any need of you to refer this patient to Kenyatta”. You are like “We don’t do this surgery in Shallom, in Machakos they said they can’t do it. So the only place we can send this patient is Kenyatta”. Then they are like “Okay, yes doc, we are okay with that but we don’t see why you are sending the patient to Kenyatta”. So that is another problem we are getting with Kenyatta.

**I: You usually talk to the nurse, or who usually…**

R: Sometimes it’s the nurses.

**I: So if you talked to a clinician is much better?**

R: Sometimes it’s nurses where they don’t even understand the condition and since the vital signs are okay, for them the patient is stable; the patient can stay in the facility, so there is no problem. If they can put a referral process whereby there is maybe a medical officer or a clinical officer. You talk to a medical officer, it’s better; you talk to a clinical officer, it’s better. Because most of the time it’s like paediatric patient when you want to send to Kenyatta, you talk to a nurse, she will tell you “That patient is okay we don’t have to receive this patient” and if you ask for a registrar who is on call that day, you talk to a registrar then ‘That patient is very sick just send the patient”. We are like, this one was refusing the patient and you talk to a registrar then the patient is accepted. That is a very big challenge with Kenyatta. I think they will address that issue. And then if they can communicate the process of referral. Whenever they say if you want to refer… like you ask me the referral process. We are part of all those hospitals referring to Kenyatta, if they can be sending like a copy “Kindly note that this is our referral process and if you observe this process, the patient will be helped”. We are waiting for that since June last year, now it’s June this year, you are the first one to inform me that there is a referral process that was made in June last year, it’s almost one year.

**I; Yeah.**

R: There is no communication, and when you call, they are like “No, you should call casualty”, you call casualty and they are like “Call the supervisor”, I don’t know the supervisor…that person who is connecting everyone to every department. You call there, he’s like “I’ve told you to call maternity”. So I’m like okay guys if it’s a case of maternity, just tell me call maternity, if it’s a case of maybe an RTA, call casualty. If they can give us numbers from departments so that is you call the department directly and the department orders people in casualty “There is a patient coming this and this and this admit, I will come to review or I’ll come to admit” that is okay. But this issue of going through a nurse, explain everything then after taking like 15 minutes, she says “Let me connect you to the doc who is around”. I’ve spent 15 minutes talking to you, and now I will spend another 15 minutes talking to another person. So, too much consuming

**I: Those are good suggestions, because I will have to give feedback to Kenyatta; part of this is actually sponsored by Kenyatta. Part of it is actually sponsored so I have to give a feedback to Kenyatta. After this one, they will get a report…**

R: At least it will be better.

**I: They will get a report on this issues especially the challenges in private facilities.**

R: At least it will be better. Let me tell you another incident; it was I think last year or last year but one. We had a case of HBH, the mother went into DIC and the gynae came do a sub-total it was not successful.

**I: Here?**

R: Yes. Then the gynae proceed with a total [inudible 21:53] but the patient was not stable, we wanted to send to Kenyatta. It took us like 3 hours until the patient died in the ambulance. Just calling; you call the ICU they say call maternity, you call maternity they say I think that is a case of emergency, you call maternity, they…It took us 3 good hours and the patient succumbed in the ambulance.

**I; That was last year?**

R: I think it was last year, if not last year then last year but one.

**I: But for orthopaedic you don’t have that much challenges?**

R: Orthopaedic, we don’t have such challenges because an orthopaedic patient you have stabilized the patient, you can send the patient. It end up sometimes you call Kenyatta they don’t pick, you call…That patient is stable and then the family is like give us the referral of hospital of choice, we shall go to Kenyatta. Then you give that referral. But they are not that much; they are not that many.

**I: Oh?**

R: Yes, they are very few, especially when it it that time of changing or shift, we can call and no one will pick that call.

**I: Because they are busy going round and overworked…**

R: No one will pick that call. But If you are handling patients, can someone be there to pick call for other hospitals because you are the main hospital referral hospital. If we are calling, it’s because we can’t manage a patient and at the same time the referral hospital don’t pick a call for two hours.

**I: It becomes an issue.**

R: Mmhh.

**I: I think that was good feedback, those are the issues that we will have to raise on the referral. But for orthopaedic cases I think it is very clear from what you have explained; it is the human capacity issue. But do you have the CT-Scans here, MRI?**

R: We have a CT-Scan not MRI.

**I: You have a CT-Scan?**

R: Yes, we have a CT-Scan.

**I: So that one works; you have a radiographer who runs the CT-scan?**

R: Yes, 24/7

**I: Pelvic injuries, spine injuries?**

R: We get spine injuries sometimes we send to Kenyatta, sometimes we send to kikuyu.

**I: But mostly you send them where?**

R: Spin, Kikuyu.

**I: Mostly Kikuyu?**

R: Yes.

**I: Why Kikuyu not Kenyatta?**

R: Personally I send them to Kikuyu. Why? I’ve tried send spine surgery patients to Kenyatta, all the time; like 95%, they are rejected. You call them “This patient has a spine injury” and all that, and then they say “Doc I think that patient just put on bed rest and then after two weeks you can review the patient”, I’m like “Are you mad? This is a spine injury you are talking about”. And then I just call friends in Kikuyu “I’m sending a patient for this and this”. Actually spine patients I have stopped completely.

**I: You get them and just…**

R: I send them to Kikuyu.

**I: And the pelvic?**

R: Pelvic yes, sometimes I send to Kenyatta, sometimes to kikuyu.

**I: But mostly kikuyu?**

R: Mostly Kikuyu.

**I: Here they can’t do pelvic as well?**

R: they can’t do pelvic as well.

**I: They can’t do spine?**

R: Yes.

**I: It goes down to what you were saying; it looks more of a capacity issue.**

R: Mmhh.

**I: But Sam can’t be coming this side?**

R: Sam?

**I: He stopped coming this side?**

R: Yeah, he stopped. I think they have issues with the hospital and then they stopped.

**I: In terms of?**

R: I think.

**I: They were not paying him?**

R: Mmhh. This hospital had some small issues and the consultants are like no we won’t come. And then paying someone like Sam, Sam is so expensive. So they stopped, but we are trying with these others and the outcome is not bad.

**I: Okay.**

R: Mmhh. We have a good outcome.

**I: I think we have gone through all the questions; I appreciate your time it was nice talking to you.**

R: Thank you.
