# Supplementary material for: Patterns of facility and patient related factors to the orthopedic and trauma admissions at the Kenyatta National Hospital: A qualitative assessment
Source: PLOS Glob Public Health. 2024 Jan 25;4(1):e0002323. doi: 10.1371/journal.pgph.0002323 (PMC10810445; doi:10.1371/journal.pgph.0002323)
Supplement: S1 File — (ZIP) [file pgph.0002323.s006.zip › KII TRANSCRIPTS/KNH KII Transcript.docx]

| **FACILITY** | **KNH** |
| --- | --- |
| **INTERVIEWER** | **Dr. Maxwell Omondi** |
| **TRANSCRIBER** | **Dora Bloch** |

**I: So, my name is Dr. Maxwell Omondi, an orthopaedic registrar at Kenyatta…at University of Nairobi; it’s my 3^rd^ year. This study is part of a part of a proposal thesis for KNH. We seek to determine the patterns and the distribution types of orthopaedic admissions to KNH; before and after the guideline was implemented. This guideline was implemented on the 1^st^ of July last year, 2021. So, we analyse data from before that; 5 months before and 5 months after that to see if the referral guideline had an effect in terms of the patterns of distribution…**

R: distribution of the referrals

**I: distribution of the referrals. Okay?**

R: Mmhh.

**I: So, apart from knowing the patterns, we also want to know the reasons behind what we see, okay?**

R: Yes.

**I: So, from your perspective, we will take about 30 minutes just to get your understanding; for how long have you been in this place? You are the in charge of A&E?**

R: I am the one in charge of the referrals; basically we do appraise our referrals every three months. We audit and…We audit our referrals and then we write a report every quarter.

**I: Okay.**

R: Yes, and I lead the team that does that.

**I: So you are in charge of the referrals for A&E department?**

R: Yes.

**I: How long have you been in KNH?**

R: This is my 6^th^ year.

**I: Oh, 6 years? 6 years, KNH. Okay, good.**

R: Yes.

**I: Where do you receive most of your referrals? Orthopaedic; you know discussion here is about…**

R: Orthopaedic referrals.

**I: Orthopaedic referrals. Which are the; you can say top 5 or top 10 facilities that you know?**

R: Top 5, I would first of course highlight Mama Lucy; Mama Lucy is on the daily basis. And then we have Mbagathi, and then we have Kiambu Level 5 and also need to put Machakos level 5 there and I would also maybe mention Thika level 5.

**I: Thika level 5?**

R: Yes, so that would be my top 5 because any day of the week you might get a referral from one of those hospitals.

**I: Orthopaedic cases?**

R: Yes.

**I: And…**

R: Most of them it will be a femur fracture, mostly femur fracture, compound and tibiofibular fractures. Most come as commonly…most trauma related.

**I: Trauma related?**

R: Mmhh.

**I: Okay, and the private facilities that refer here?**

R: No, it’s not as common. The private facilities that refer to us, I would say level 1, 2; those small private facilities that receives a patient as a… That interact with a patient first probably from an accident scene and if I was to think of one at the top of…at my finger tip, I would probably say a hospital like Sinai. I don’t even know where it is, but just from interacting with the referrals, I was able to note some small facilities. Another one is Kitengela medical something…

**I: Kitengela medical services?**

R: Yes. But you wouldn’t really get patients from say St. Mary’s mission hospital or for example Nairobi hospital, or Nairobi West. Unless the patient has a financial constraints. The patient presents there but because of financial constraints, would once or twice get a referral from top flight private facilities.

**I: So, from your observation since the guideline came into effect on the 1^st^ of July…**

R: July, yes.

**I: Has there been a change in terms of referrals from this facility?**

R: Yes. To be honest, there has been a big change. First of all, we’ve not had a volume of like the ones we used to have before. Because before, basically I think they would just refer any patient. Basically, you would hear patients mention things like “I reached Mama Lucy’s gate and was told by the watchman to go to Kenyatta; that I should take this case to Kenyatta”. [Giggling] We’ve really had…

**I: By a watchman?**

R: Yeah, that was actually quite common before. We started engaging this hospital. In fact we had a team from A&E who visited with them and we have made contact of how we give feedback. So, if we receive a referral that we feel does deserve or does not meet the regulations, we do give them feedback; we call the hospital in charges and we give that feedback; we say that “We have received a patient, that so and so has taken a referral note, we don’t feel that this patient deserve to come here. That has really…I would say, we have noticed a very big difference; the patients we do receive now, are genuine referrals, the ones that truly deserve to come here.

**I: The genuine referrals?**

R: Yes. Also, the engagement between the referring facilities and our department have really improved.

**I: What do you mean by improved engagement?**

R: For example, before no one really used to… They wouldn’t call before they bring a patient and even if they do call, it’s probably some one really junior probably a nurse referring the patient or a clinical officer who has been boxed to refer a patient. But ever since we started engaging these facilities, it becomes now a doctor… the majority of the referrals we receive now are referrals from either a medical officer or a consultant seeing the patient but majority of them are by medical officers. At least you are able to get the facts right before and…we do refuse those referrals; they make the phone calls and we are like “No, this patient can benefit from Mama Lucy, he doesn’t have to come to KNH. So, I say engagement; like one on one engagement between the department and those referring facilities, has improved unlike before where they wouldn’t call, just in triage you get a patient with a closed tibiofibular fracture and is not displaced. You just send them to the plaster rom and they get a plaster and some pain killers and they go back home. You see this patient coming with a very big referral form from Mama Lucy. That has really reduced.

**I: That has reduced?**

R: That has really reduced.

**I: Is there a change in patterns of the types of injuries. Like we are saying mostly the ones that have been coming are have been femur and tibiofibular shaft fractures. Has that pattern changed or even the current one is of the same pattern?**

R: No, the pattern has not changed; it’s still the same. I think the only thing that has changed is now is I would say, they are not referring to us fractures that can be managed conservatively. You find that majority of the ones that they refer usually ends up in our wards; we don’t end up discharging them at the point of entry.

**I: Before you used to…**

R: Before it would be everything; before we would be having a queue of patients; orthopaedic patients, orthopaedic review patients and they are all discharged from there.

**I: At least that aspect…The guideline has helped to streamline that aspect?**

R: Yes.

**I: So that you don’t get cases that can easily be managed?**

R: Yes, exactly.

**I: And the fact that senior colleagues are now calling…**

R: Are calling.

**I; Rather than junior guys.**

R: Yes.

**I: So that…**

R: Not anyone who interacts with the patients there.

**I: Yes, before it used to be anybody who sees them?**

R; Yes. Before it used to be very bad. Like, you would get someone from Mbagathi District hospital with a very small cut and when he comes he says “I have been told in Mbagathi this issue is for Kenyatta; you should just go to Kenyatta”. When you look at it, or they even come with a very big dressing and when you open, it is just a small cut.

**I: That…**

R: And they come with a big referral saying for Orthopaedic review suspect fracture; for X-ray and orthopaedic review.

**I: Suspect fracture?**

R: Yes.

**I; they’ve not been examined?**

R: No, they have not examined the patient. That was very common; it was very common.

**I: That was from all the referring facilities?**

R: Yes, especially the ones around Nairobi; Mama Lucy, Mbagathi, that was very common.

**I: Even Mamma Lucy, juniors used to call?**

R: Absolutely

**I: All these you are giving is from most of the facilities?**

R: Yes, absolutely; majority of the facilities used to do that. Majority of the referrals were done by nurses.

**I: Were done by nurses?**

R: Yes, before these guidelines. Before that enforcement of the guidelines here, majority of the referrals; the phone call referrals, the ones that used to be at least a call, was probably by a nurse referring a patient. A junior nurse for that matter, or the ones called…They are called what? The matron.

**I: The matron?**

R: Yes, the hospital matron.

**I: They are the ones who are mostly…**

R: Yeah, most of the times they are the ones who were referring.

**I: And the walk-ins have been reduced or they are still the same?**

R: The walk-ins have been reduced. In fact…Sorry, I think I need to correct that statement, I think what we have done now, is we have a triage desk. So we still have a lot of walk-ins even with fractures but we don’t down refer the fractures, we see them [inaudible 11:18] it does not matter how the pattern of the [inaudible 11:20] is. We still see them. We still have a lot of walk-ins but we try to triage in the ones that need to come in and we down refer the rest.

**I: So, walk-ins…I’m talking about admissions, not just orthopaedic, but those who end up being admitted. You still have…**

R: We still have walk-ins yes.

**I; Are they many, has it reduced?**

R: No, they have reduced.

**I; Pardon?**

R: They have reduced.

**I: Has reduced?**

R: Yes.

**I: And the pattern, what is the commonest pattern and types of injuries that used to be admitted; walk-in?**

R: The ones that used to walk-in?

**I: Yes.**

R: Maybe you need to define for me walk-in...

**I: Walk-in are those ones who don’t have referrals.**

R: They don’t have referrals; they are probably from an accident scene?

**I: Yes.**

R: They are the majority actually.

**I: Why?**

R: The ones who are brought it by…They are called who…The emergency responders?

**I: Yes.**

R: The St. John and the rest?

**I: Yes.**

R: They are the majority.

**I: They are the majority?**

R: Yes, by far.

**I: That one has not changed?**

R: That one has not changed.

**I: I thought the guideline is supposed to sensitize everyone around?**

R: Exactly, I agree with you. In fact the challenge we have is actually from not even the referring facilities, but from basically the general…

**I: Public.**

R: the general public. Because even if…The reason why our numbers have reduced, is because the general public still believes that KNH is the hospital for treatment. So, unless for some whisker they find themselves in Mbagathi, the first hospital they will always think is KNH.

**I: So they see KNH as the first facility of choice?**

R: Yes. So, we still get even those ambulance guys, they don’t make phone calls. Majority of them just land here. They are from an accident scene and this is the nearest hospital.

**I: Are they aware of these referral guidelines?**

R: They should, actually they should.

**I: They are aware?**

R: They are aware.

**I: But they still opt to…**

R: They still opt to come here. Because, I don’t know; sometimes you get a feeling that they get a better reception here than in these other facilities.

**I: Due to better reception?**

R: Yes. Because when they bring the patient here, we don’t refuse the patient. We don’t tell them to now take the patient to another hospital. We always receive the patients.

**I: walk-in s still remains as it is?**

R: Walk-ins are still the majority.

**I: And even the pattern has remained as it is?**

R: The pattern has remained as it is.

**I: Nothing much from what you are saying seem to have changed?**

R: No.

**I: What exactly are the referral guidelines; is there a documentation, do you have where this is what the guidelines says? Is there a written…**

R: No we don’t have a written document. The thing that, or rather what we are doing here in the department is, basically after the team visited several high volume facilities around, they were able to determine the resources that these facilities have.

**I: There is a team that went round?**

R: Yes. There is a team that went round; they went round. Majority of the counties have been visiting all the way to Isiolo, I think Marsabit.

**I: Marsabit is a county?**

R: Yes. So, after that especially the ones around us here; Mama Lucy, Mbagathi, Kiambu level 5, Gatundu, Thika level 5, that team went round. We were able to establish the resources that they have. So, when we get a call, usually the team leader doctor down there is informed of a phone call that is not so…For example it’s an obvious say a femur fracture and we get that phone call from for example Mama Lucy, the people at the referral know that Mama Lucy have a surgeon

**I: And they have the capacity.**

R: And they have the capacity to manage that fracture. So, if they need to refer that patient, then the referral need to come from the consultant. The consultant has to write the referral note and the consultant should make the phone call. Even in the department we have a consultant on call; we have an orthopaedics on call and we have a general surgeon on call. If we still feel like; if the team in the referral feel like they still don’t need them, that the patient can be managed, if the referring doctor doesn’t give sufficient reason to refer, we consult the surgeon on call and then the surgeon now gives consent for the patient to come.

**I: The referral office has an overview of the capability of most of these facilities?**

R: Yes.

**I; They can make that decision; that no this one you can manage, this one you can bring?**

R: Yes.

**I: There is no hard copy, it is just based on that assessment?**

R: No, no hard copy it is just based on that assessment.

**I: That assessment there is a documentation that is…**

R: There is a report that was written, so we are…We have not really transcribed it like a guideline on which hospital has the resource or what, no. it is just that now…Previously what we used to have is that the person at the referral office also used to be a nurse, a nurse team leader. And they would just receive as many as they can when they call. Before, the calls were not as many as now. But now the difference is, when the nurse receives a phone call, they consult a team leader; we have a team leader doctor throughout the shifts in that department. The doctor usually; from experience, a medical officer, makes that decision whether the patient comes in or not. If he feels like he needs further clarification, he consults the surgeon on call. Even there was a time we had seating orthopaedic surgeons, the senior resident. They would actually be the ones who made the decisions on whether the patient comes in; especially the neurosurgery team because that’s a bulk of patients that get referred. The sitting neurosurgeon based in casualty usually makes the decision on behalf of the entire team.

**I: Even for the orthopaedic cases?**

R: Even for the orthopaedic cases yes.

**I: The neurosurgeon makes that decision?**

R: yes.

**I: There is an orthopaedic person who makes that decision?**

R: Yes,

**I: For the registrar?**

R: For the registrars. Yeah.

**I: That means they are informed, they know what…**

R: Yes.

**I: Orientation has been done to them?**

R: Umm really no.

**I: What are the findings of this committee that went round collecting?**

R: The team leading doctors are our senior doctors and they are part of the team that went round. But since it become like a team thing to receive patients, majority of the time the people…Because it’s specific people; team leaders, the nursing team in referral office, there is a specific team. So, over time they got the hang of it, but it’s not really a guideline; they don’t have a guideline per se.

**I: So, that report that you have, can it be accessed, is it possible to share?**

R: Yeah, I think you should be able to have that report. You can ask her.

**I: She has hard copy or email, which one would be the…**

R: I am not sure, even her…But I think she has them on soft.

**I: Soft?**

R: Yeah.

**I: So, I can give you my email and then…**

R: Yes, we can give you the report.

**I: She’s called who?**

R: She’s called Florence

**I; Oh, Florence, the secretary?**

R: Yes, she’s the secretary to the office.

**I: The kind of patients you receive, the profile, has it changed? Like distribution…**

R: In terms of…

**I: Age, in terms of the elderly paediatric cases; more elderly coming in, more… That has not changed?**

R: That one has not changed.

**I: Pardon?**

R: That one has not changed. Actually, the distribution is still the same; majority of the cases

**I: The patterns, even the distribution has remained the same?**

R: Yeah.

**I: It’s not that now we are seeing more spinal cases compared to before…**

R: Previous.

**I: More or less…**

R: More or less the same. More or less it’s the same thing that we used to see before. It I just that before we had more of patients who discharge on entry. Right now we don’t discharge on entry, majority of the patients we actually admit.

**I: Majority, so before you used to discharge on entry?**

R: Yes.

**I: You discharge on entry?**

R: Yes.

**I: But now, more or less you admit**

R: Yes. More or less, we admit the majority. In fact you will find the orthopaedic cases that comes in, if it’s a referral you will most likely admit that patient, but if it’s a walk-in, the walk-in we end up discharge from entry, from casualty.

**I: The walk-ins…**

R: Yes. The walk-ins are the ones that really contribute to our non-admissions. But the ones that we receive as referrals, end up being admitted.

**I; So, discharged on…**

R: On presentation.

**I: On presentation.**

R: Yes.

**I: as compared to referrals, so referrals most of the time will end up in the wards?**

R: Yes.

**I: Because they have already been discussed and they are genuine cases?**

R: Yes.

**I: So, in terms of profile nothing much you have seen changed; referrals, in-patient, type of structures.**

R: No.

**I: That has remained more or less the same?**

R: Yes.

**I: What are the main reasons they say; or the main reasons for the referrals from these facilities? What are the common things they tend to say? Like Mama Lucy for example?**

R: The things they tend to?

**I: The reasons why they are referring.**

R: Oh.

**I: Yes.**

R: Majority of time they will be things like…

**I: Like Mama Lucy for example, why would…**

R: Mama Lucy will be like, we don’t do…Orthopaedic surgeon is not there.

**I: No orthopaedic surgeon?**

R: Yeah, no orthopaedic surgeon. Actually, that is the commonest thing; we don’t have an orthopaedic surgeon. Sometimes that would be the reason given when we know a facility has an orthopaedic surgeon. So it will be a fight; “we know you have an orthopaedic surgeon” “no, we don’t’”. That is the commonest reason. The next common is theatre not functioning, their [inaudible 23:24] is not convertible in treating the patient here or especially patients who are politaruma patients; who probably have also head injury. They will be like “We are referring because the patient requires neurosurgery review or general surgeon review and we don’t have that here.

**I: They don’t have a general surgeon as well?**

R: Yes.

**I: Or the…**

R; Or commonly the neurosurgeon.

**I: The neurosurgeon?**

R: Yes. So politrauma patients are the ones that you will find they be like “They need ICU”. The other common thing is the ICU space if a patient requires ICU. That is one of the biggest; actually I would say that is the biggest reason for referral even for patients who have not been attended to.

**I: Bigger than even not having orthopaedic surgeon?**

R: Yes. Especially politrauma patients; patient is unconscious but it’s a compound tibiofibular, on top of it, it will be like “We need an ICU space and orthopaedic review. The patient will come here and you will examine the patient and the patient doesn’t require theatre. The only thing that is looking at you is the exposed wounds, but otherwise the patient is okay other than hypotensive, things like that but no. you will just see they were just escaping from the fracture.

**I: Those are the main ones for Mama Lucy, and for Mbagathi?**

R: Basically it is just the same; the reasons scatter. It’s like a culture I would say. I will call it a culture; it’s like a common thing.

**I: Machakos level 5 also?**

R: Machakos level 5 I wouldn’t comment; I wouldn’t compare them with Mama Lucy and Mbagathi. I think Machakos referrals are more quality [background noise, inaudible 25:49]. You will not find fault, rather…

**I: They are genuine referrals?**

R: Yes, they are genuine referrals.

**I: And Kiambu level 5?**

R; Kiambu level 5 are the same thing with Mbagathi; their patients are always coming here and they are stable patients, patients that probably need a POP.

**I: And Ngong Sub-County?**

R: It is the same; absolutely the same as… In fact I remember Ngong Sub-County is one of the facility which the team visited because we were wondering why they were not referring their patients to the County referral hospitals. But I think for convenience sake, they were finding it easier to refer patients this side, so they will end up referring…

**I: Because of distance?**

R: Yeah, distance.

**I: They prefer KHN to Kajiado.**

R: They prefer Kajiado to KNH, other than Kajiado referral county hospital.

[Interruption]

**I: And this private hospitals St. Peters orthopaedic?**

R: I’ve never had a referral from St. Peters. Have you?

R2: Yes.

**R: They bring their referrals?**

R2: Yes, they bring.

**I: They do?**

R: Personally I’ve, but maybe they do.

**I: Reason for that?**

R: The same thing; orthopaedic review, they don’t even write any [giggling] that is what they write; orthopaedic review, neurosurgical review, nothing else. If you ask “You don’t have an orthopaedic?” “No”. they look at you.

**I: They just look at you?**

R: Yes.

**I: Those are the main reasons, not even patients…Do patient’s issues; do patients also have a preference where they are being referred? Do we have such cases where…**

R; Yeah, I do agree. Some actually call and say patient…

**I: Requested.**

R: Requested yeah.

**I: The reason why they are requesting?**

R: Sometimes it’s a psychological thing; KNH is a big hospital and I think word has gone round that we don’t charge that much.

**I: Pardon?**

R: Financial constraint is like the commonest thing ever. Financial constraint brings problems mostly.

**I: It’s the big one?**

R: Yeah, financial constraint.

**I: Is the reason the like to come to KNH because they see KNH not to be…**

R: Is cheaper compared to…

**I: Compared to?**

R: You see, it’s cheaper and most trusted in terms of quality of treatment.

**I: Quality of care.**

R: Others want their second opinion.

**I: And others come…**

R: For second opinion.

**I: Issues about infrastructure, availability of equipment, are those being cited as potential reasons for…**

R: I think yeah, I think that is actually…You will get things like X-ray especially Mbagathi. Mbagathi’s reason for referral always is the machine is not working every day.

**I: Not working?**

R: Not working; our X-ray is not working.

**I: And the plans, what the plans for things like that?**

R: No.

**I: No?**

R: No.

**I: Space? Infrastructure?**

R: Theatre a space.

**I: Theatre a space.**

R: Yes. Theatre space is the thing, every day

**I: They say they have no theatre space?**

R: Yes.

**I: That is for all these facilities?**

R: No, basically Mbagathi and Mama Lucy

R2: And Kiambu Level 5. The ones which are near KNH.

R: The ones which are near are Mama Lucy and Mbagathi.

**I: Kiambu level 5?**

R: Yes.

**I: What are some of the recommendations you would give; I think we are coming to a close. What are some of the recommendations you would give for us to try and address these issues?**

R: I think number 1 is, we need a we need a new way of managing health care generally as a country because many time you will find the issue is not lack of personnel but rather lack of commitment. You will find a hospital like Mama Lucy they have everything; the orthopaedic surgeon is there, a functional theatre, a functional radiology unit. But still, depending on who sees the patient whether is the doctor or the nurse, if…

R2: Unavailability of the consultant specifically in those…For example we had a case actually it was a [inaudible 31:21] I was sorting it this morning with the perforated DU from Mama Lucy. This patient was admitted in Mama Lucy almost like…he stayed in Mama Lucy for 8 hours with perforated DU then they brought to Kenyatta. You wonder. They have a surgeon there, they didn’t have any issue with the theatre. So basically the patient [inaudible 31:43].

R: So yeah that culture thing of people being committed to work, that needs to change. Because you will find even Mama Lucy they will receive two patients at night but they will prefer to send those patients to KNH, or admit them to their ward as the doctor has said and do nothing about it.

R2: When they deteriorate is when…

R: Yes, when the patient deteriorates, is when…

**I: So what are our recommendations for that especially for orthopaedic referrals, what would be some of the…**

R: That will be number one.

R2: Commitment.

R: Yes, we need people to be…We need to find a way of enhancing or enforcing commitment for people to see patients. Because if you see a patient; even if now you see a patient; if you take time and see a patient, immediately from the time a patient reaches you, you can be able to make that decision and say you refer this patient with a proper good reason, rather than keeping that patient and when they start deteriorating is when you going helter skelter. Number 2, is human resource. I think we still have a very big problem in terms of not only the numbers but also quality. So, if the referring facilities can be facilitated with trained orthopaedic surgeons and those orthopaedic surgeons are facilitated because they can improve on the kind of referrals…they can have an impact on the referrals that we get.

**I: But in Mama Lucy we have orthopaedic surgeon?**

R: Exactly, so it’s not like they don’t have surgeons.

**I: Mbagathi have?**

[Background noise 33:48]

R: There is something he was telling me, what about theatre spaces?

**I: Theatre spaces, huh? To increase theatre spaces?**

R: Yes, probably to increase theatre spaces. The other thing is that, what needs to come clearly is what needs to be referred to Kenyatta.

R2: The referral system.

R: That needs to be a document written and taken to every referring facility. We need a written guideline; clear guideline on the referrals.

**I: On referral?**

R: On…

R2: Who needs to come to Kenyatta?

R: Which kind of femur fracture needs to come in.

**I: Which one must you manage.**

R: Which one must you manage yourself?

**I: so that guideline is required.**

R: That guideline is required. The one that you have received a phone call from St. whatever, St. Paul, I tell them “According to this guideline which you also have, this patient needs to go to Mbagathi”.

**I: Any other recommendations?**

R: I think that’s it for now.

**I: I think that was great, that’s a good overview. I appreciate your time. I think I have your number; Dr, Sam K.**

R: Yes.

**I: In case I need any clarification at some point, I might just call you to clarify**

R: Just call me anytime

**I: Because I’m going to do KIIs for all these referral; the ones that you have mentioned to also get their perspective and then we consolidate and then I’ll do a report and share it with KNH. So you will probably be one of the people when I’m disseminating, you will have a copy and see what are the issues from everyone. We can relate then we can get what are the findings and the recommendations, then we share with KNH.**

R: Okay.

I: That was great. Nice meeting you.

R; Nice meeting you.
