# Supplementary material for: Patterns of facility and patient related factors to the orthopedic and trauma admissions at the Kenyatta National Hospital: A qualitative assessment
Source: PLOS Glob Public Health. 2024 Jan 25;4(1):e0002323. doi: 10.1371/journal.pgph.0002323 (PMC10810445; doi:10.1371/journal.pgph.0002323)
Supplement: S1 File — (ZIP) [file pgph.0002323.s006.zip › KII TRANSCRIPTS/Machakos County Referral Hospital Dr Ayuka.docx]

| **FACILITY** | **Machakos County Referral Hospital Dr Ayuka** |
| --- | --- |
| **INTERVIEWER** | **Dr Maxwell Omondi** |
| **TRANSCRIBER** | **Dora Bloch** |

**I: I’m orthopaedic registrar at Kenyatta. I’m doing my proposal, for the last two weeks I’ve been in touch with Dr Nyamwea and he referred me to Dr Nderitu, and Nderitu finally referred me to you. He told me you are busy…**

R: Yeah, I was actually in theatre that time.

**I: He told me you were in theatre.**

R: Yes, I was doing some [inaudible 00:24]

**I: Were you in UON also?**

R: No, I was not in UON.

**I: You were not in UON?**

R: No. I was at UON for undergraduate.

**I: This is just the story on UON, for clearance. I just wanted to do a small KII.**

R: Pardon?

**I: Key informant interview that will probably take us few minutes and basically we want to understand the issues around orthopaedic referrals from Machakos to Kenyatta. I just wanted to know where do you normally refer most of your orthopaedic cases.**

R: The only place you can refer is Kenyatta which is a level 5 hospital.

**I: Kenyatta?**

R: Yes.

**I: Is there any other facility you refer to?**

R: They are already a level 5, so the only other place we can refer to is a level 6.

**I: Okay level 6.**

R: So only Kenyatta yes.

**I: Is there any other private place you refer to?**

R: For?

**I: Private place you refer to?**

R: No, maybe just Kenyatta.

**I: Only KNH?**

R: Yes, only Kenyatta. And it’s only spine

**I: Yes?**

R: Only spine cases.

**I: Only spine cases?**

R: Yes, all other things we do.

**I: Pelvic?**

R: We do.

**I: You do pelvic?**

R: We do pelvic, we do hip, we do knees, but we are not doing many; we do arthroplasties…oh and arthroscopy, we don’t have a tower

**I: So you also refer arthroscopy cases?**

R: Arthroscopy yeah.

**I: Arthroscopy cases and spine cases?**

R: Spine cases yes.

**I: Are you aware of this KNH referral guideline that was enforced from 1^st^ of July?**

R: I’m not sorry.

**I: You are not aware about that?**

R: Mmhh.

**I: But usually when you refer, the first thing you have to call; is it you who has to call or it is the MO to call?**

R: It is the MO; that is the work of the MO.

**I: That is the work of the MO?**

R: Yes.

**I: They just make the necessary calls and they have the thing…**

R: Yeah and then they refer, but the decision is always made at the [inaudible 02:45] that this is a case that we cannot be able to handle, call Kenyatta and refer to Kenyatta. Oh, we usually do spinal injury, sorry.

**I: Sorry?**

R: We also do spinal injury, for guys who can be able to afford.

**I; You also do spinal injuries?**

R: Yeah, spinal injury, to spinal injury hospital.

**I: You refer to spinal injury hospital?**

R: yes, spinal injury hospital, sorry I had forgotten.

**I: So, apart from KNH, you also refer to spinal injury hospital?**

R: Yes.

**I: What kind of patients do you end up referring; these spine cases and athroscopy; what are their profile?**

R: Mostly trauma; trauma spine.

**I: Mostly trauma spine?**

R: Yeah, trauma spine.

**I: And age wise; what age bracket are they in?**

R: Mostly young guys; because most situations are from motorcycle accidents.

**I: Motorcycle accidents.**

R: Yeah, that age bracket; mostly men, mostly young guys below 40.

**I: Below 40?**

R: Yes.

**I: In terms of economic status, do they have insurance?**

R: Of course not, they don’t have.

**I: But most of them…**

R: Those who have insurance prefer going to spinal injuries, but those who don’t have they end up in Kenyatta.

**I: They end up in Kenyatta?**

R: Yes.

**I: The cases, are they paediatrics or are mostly adults?**

R: Adults.

**I: They are actually adults?**

R: Yeah, adults.

**I: You don’t usually refer paediatric cases?**

R: I have never seen, leave alone referring; I’ve never seen.

**I: You have never seen one?**

R: Yes.

**I: The reasons for referral; the reasons that you refer these patients are mostly are…**

R: We don’t have sets.

**I: Sets for spine?**

R: Spine yeah.

**I: No sets for spine.**

R: No sets for spine.

**I; What else?**

R: Basically that’s it and maybe implants.

**I: Implants?**

R: Mmhh, sets and implants.

**I: Sets and implants**

R: Yes, those.

**I: Missing.**

R: Yes.

**I; Do you have a neurosurgeon? If you have politrauma patient, how do you handle?**

R: pardon?

**I: Politrauma cases; those who have got head injury together with…**

R: We have a neurosurgeon. If they have head injury plus trauma, it is not spine, we usually sort them out.

**I: so most of the cases, what you don’t sort out is spine?**

R: The spine yeah; specifically spine.

**I: Specifically spine?**

R: Mmhh.

**I: Implants you have; you have enough implants for all those; long bones…**

R: We don’t have. The patients buy.

**I: Usually they buy?**

R: Yeah, they buy.

**I: Either cash or NHIf.**

R: Either cash or NHIF, they usually buy.

**I: Issues about infrastructure, do you have enough theatre space?**

R: There can never be enough theatre space…

**I: But it is not a reason for…**

R: We operate two days a week; Mondays and Wednesdays, and we have two beds…

**I: Two beds?**

R: Two operation tables.

**I: Two operation tables?**

R: Yes, so it’s 4 beds per week. It’s not enough but we are okay.

**I: What space…You have an isolated orthopaedic ward?**

R: yeah, we have male and female orthopaedic wards.

**I: That’s a big facility.**

R: We have a big facility.

**I: These spine cases, are there scenarios where the patient actually want to be referred on their own preference to come to KNH?**

R: Sometimes but rarely. But most of the time is that we cannot be able to sort this case out, we have to go to another facility.

**I: You have to go to another facility.**

R: Mmhh.

**I: How many orthopaedic surgeons are there in Machakos?**

R: Currently we are 1, 2, 3, 4.

**I: You are 4?**

R: Yes.

**I: Where shall we go; where will some of us go when we finish?**

R: There is a lot of work [laughing] like in Machakos we have many facilities; Kangundo has two, [inaudible 07:41] has one, those are around 7 in the county, but the work is also a lot.

**I: The work is a lot.**

R: Mmhh.

**I: You’ve been there for how long?**

R: I’ve worked there for…Since 2018.

**I: 2018…Even…she will be coming there**

R: Who?

**I: Katuse**

R: She’s the 4^th^ actually; because me, some guy called Musi, there is Ndeda and I hear there is new, she’s new I’ve not met her.

**I; Katuse.**

R: Yes, Katuse I think.

**I: But she hasn’t come yet; she is still doing her final exams.**

R: Oh, she hasn’t come?

**I: Not yet.**

R: There is one who reported.

**I; Sorry?**

R: There is one who reported, I don’t know her name; I’ve not met here yet.

**I: Has reported…**

R: But she is around.

**I: But Katuse is a finalist, she will be doing her exams this…**

R: Not Katuse, there is one more; some lady.

**I: Any recommendations you’d make regarding these orthopaedic referrals, management of orthopaedic cases between KNH and Machakos?**

R: If we are able to get the requisite sets, then we will actually be sorting them out in Machakos.

**I: That is the county government; if the county government can…**

R: Yes; if we are able to get the sets for spine. Because we are all trained on spine; at least some bit of spine especially trauma; stabilization and things like that. If we get those requisite equipment, we will be able to sort them out; we won’t need to refer.

**I: You won’t need to refer?**

R: Yes.

**I: That referral process…There is also another thing you said you refer, arthroscopy. What of arthroscopy?**

R: Arthroscopy, those ones we don’t have a tower

**I: You don’t have a tower?**

R: Yes. Those ones I think I have referred some to kikuyu, because even in Kenyatta do you really have tower?

**I: Kenyatta usually the implant guys come with their own set.**

R: They come with the tower.

**I: The implant guys.**

R: I had forgotten, I refer some patients to kikuyu if they have.

**I: For arthroscopy?**

R: Yes, arthroscopy.

**I: To Kikuyu?**

R: Yes, to Kikuyu. Especially NHIF guys, they reserve clients

**I: The implant guys at Machakos can’t come with a tower?**

R: I’ve never seen them.

**I: Those are the issues; the issue of implant is not a big issue from what you are saying except for the spine. The long bone, the pelvic, you have all the implants; you have the sets?**

R: If they are able to afford the implants; the good thing is that the implant guys come with the sets.

**I: For the pelvic or for the long bones as well?**

R: Long bones, pelvic, everything?

**I: They come with the sets plus the implants?**

R: They have to come with full set; that is our rule. When we joined, we used to get someone coming with one recon plates and some screws. That one we said no.

**I: You just come with a full set.**

R: Yeah, you have to come with a full set. Full set with a set of screws so that we can measure and put the collect screws.

**I: You have options to choose from.**

R: You have options yes. Sometimes you find someone coming with all of them size 20, and you need 14.

**I: And then now you are forced to improvise…**

R: Now you are stuck; you have already opened. But the good thing is the implant guys come with the set.

**I: They come with the sets and everything.**

R: Mmhh.

**I: The catchment population for Machakos; the orthopaedic case, they come from which areas mostly?**

R: Mostly Machakos; we used to receive from Kajiado county, Kitui before they got someone; they got someone last year who has been very active.

**I: Mwingi, or where?**

R: Kitui County.

**I: Oh, Kitui?**

R: Yes, there is a guy who joined there; there is a new guy who joined there. But ever since, we have been getting; we have been treating Kitui cases a lot.

**I: And Mwingi?**

R: Mwingi is part of Machakos.

**I: Do they also bring to Machakos?**

R: Yes, all Machakos refer; even Kangundo, for patients they cannot be able to sort out, they refer to Machakos.

**I: You guys have ICU?**

R: We have ICU, we have HDU.

**I: ICU, from which year was it there? It has been there since last year?**

R: It has been there for long.

**I: It has been there for long?**

R: Yes, it should be around 6/7 years; from around 2016.

**I: Because I talked to a guy in Mwingi and they said you guys did not have an ICU so they used to refer patients to Kenyatta because…**

R: We have ICU, but during COVID times it was always full.

**I: It was always full?**

R: Yes. Because it was only 4 beds.

**I: ICU is only 4 beds?**

R: Yes, only 4 beds. If it is full, of course the patients have to be referred.

**I: The reason maybe they kept calling and they are told no beds, no space.**

R: Yeah, no beds sometimes no equipment.

**I: Those are the things that I wanted us to talk about; I don’t see anything else we haven’t discussed. We have looked at the common cases that you refer; you have talked about those trauma cases spinal injury especially, arthroscopy cases. You have talked about the profile of the patients…**

R: The what?

**I: You have said mostly they are adult male.**

R: Adult male, yeah.

**I: Those ones for road traffic accidents.**

R: Yeah, RTAs.

**I: RTAs.**

R: yes.

**I: And you mostly refer them to Kenyatta, Kikuyu and spinal injury.**

R: And spinal injury yes.

**I: Those three, but mostly it’s Kenyatta.**

R: Yes Kenyatta.

**I: Okay, that is it I have for now. In case of any additional issue, I would like to call you for a few clarification.**

R: In case of anything just give me a call, no problem.

**I: Okay.**

R: Thank you very much.

**I: thanks**
