# Supplementary material for: Patterns of facility and patient related factors to the orthopedic and trauma admissions at the Kenyatta National Hospital: A qualitative assessment
Source: PLOS Glob Public Health. 2024 Jan 25;4(1):e0002323. doi: 10.1371/journal.pgph.0002323 (PMC10810445; doi:10.1371/journal.pgph.0002323)
Supplement: S1 File — (ZIP) [file pgph.0002323.s006.zip › KII TRANSCRIPTS/MAMA LUCY KIBAKI HOSPITAL KII.docx]

| **FACILITY** | **MAMA LUCY KIBAKI HOSPITAL** |
| --- | --- |
| **INTERVIEWER** | **Dr Maxwell Omondi** |
| **TRANSCRIBER** | **Dora Bloch** |

**I: We can have a brief introduction, myself is Dr Maxwell Omondi, I’m the researcher and also a resident at the University of Nairobi. I’m doing orthopaedic surgery. You can introduce yourself.**

R: My name is Veronica Wanjiku Gitonga

**I: Veronica?**

R: Wanjiku Gitonga.

**I: Wanjiku Gitonga.**

R: I’m the head nurse here in my department.

**I: Head nurse?**

R: Yeah.

**I: For A&E?**

R: Yeah. Chief Registered Officer.

**I: As a [inaudible 00:49] to you, I would like to get permission from you to basically take this interview to understand the referrals of orthopaedic cases in KNH. This study is part of my masters of orthopaedic studies and in July last year, KNH put in an enforcement of referral guideline that says not all patients should be seen at KNH. There should be guidelines to bring in patients [interruption]…**

R: Sorry.

**I: So the guideline was enforced on 1^st^ of July, so I wanted to see the enforcement of this guideline is there any difference in terms of referring patients and this is orthopaedic cases. There are few questions I will be asking you together with that; they are about 5-6 questions. Where do you refer most of your orthopaedic cases?**

R: To Kenyatta.

**I: To Kenyatta?**

R: Yes.

**I: Why Kenyatta or there are other places you refer to?**

R; Those who are able, they can request to go to Kijabe or Kikuyu, But most request to go to Kenyatta.

**I; Others to Kijabe…**

R; Or Kikuyu.

**I: But mostly…**

R: Those who are able to pay for their cases, but mostly they go to Kenyatta.

**I; Those who are able to pay?**

R: Yes.

**I; But mostly is what percent?**

R: At Kenyatta?

**I: Yes.**

R: Kenyatta, is like 70%.

**I: Say in a scale of 1-10, how many go to Kenyatta?**

R: I think 7 to…

**I: Pardon?**

R: About 7 out of 10 go to Kenyatta.

**I: 7?**

R: A scale of 7, mostly they go to Kenyatta.

**I: 7 out of 10?**

R: Yes.

**I: Go to Kenyatta?**

R: Yes. And these ones are like mostly fracture

**I: They are fracture…**

R: Femur mostly.

**I: Fracture femur mostly.**

R: Yes, and head injury.

**I: And head injury?**

R: Yes. Simply because we have orthopaedic surgeons but we don’t have the space and also a neurologist is not here.

**I: There is no neurologist?**

R: Yeah

**I: But orthopaedic surgeons are there?**

R: They are there.

**I: They are present.**

R: There is one Dr Rono, there is one from JKUAT.

**I: Dr Rono from JKUAT?**

R: Yeah, Dr Rono is for the hospital, the other is from JKUAT, Dr Oluwa.

**I: He comes here?**

R: Yes, on Monday there is clinic.

**I: He’s orthopaedic but he’s employed by?**

R: JKUAT yeah.

**I: But he comes to do what?**

R: He comes to see the patients.

**I: It’s part of the teaching or what?**

R: As part of the teaching yes.

**I; He comes with students?**

R: Yes.

**I: They have students here?**

R: Yes.

**I: Oh, but mostly is Rono who is…**

R: Dr Rono yes.

**I: When you say space, what do you mean?**

R: The bed capacity here, hmmm average is full.

**I: Is what? What is average, about how many beds?**

R: Recently we got a new ward, but I had gone out to another hospital; I was at Korogocho I haven’t been back here. Actually I am one week old, by then they had not opened the new hospital but I can ask for you how many patients are there. I’ll go ask for you…

**I; So you are one week old here?**

R: Yeah, one week old here. But before I was working in this department.

**I: When were you working in this department?**

R: I have worked for almost 6 years now.

**I: Last year you were here?**

R: Yeah last year, what date…Yeah last year I was here I left in October and came back here last week.

**I: Last week?**

R: Yes.

**I: Between October and now, who was in charge?**

R: What is her name…umm Eveline.

**I: Eveline?**

R: Yes, but she left for the USA.

**I: To USA?**

R: Yes.

**I: Then you replaced her?**

R: Yes.

**I: So, the bed capacity you say is small.**

R: Yes.

**I: Because of the space you are forced to refer?**

R: Yes.

**I: What of Tibia?**

R: Tibia is okay, we are able to manage from here.

**I: You manage it here?**

R; Yeah, other fractures we are able to manage them here.

**I: What is the difference between Tibia and fibula…Tibia and femur? Why not femur?**

R: Tibia we have the ward for…Because tibia by the time they need surgical, if they are compound then they can be admitted. I see Dr Rono admitting some of them actually.

**I: Tibia?**

R: Yes.

**I: Why tibia and not femur? Why do you refer femur cases to KNH and not tibia?**

R: Dr Rono says there is no equipment for the tibia here.

**I: No equipment?**

R: Yes, for the femur.

**I: By they have for tibia?**

R: Pardon?

**I: They have for tibia?**

R: Tibia we have yeah.

**I: So, they don’t have equipment for femur?**

R: Mhhh.

**I: So all femur patients you refer?**

R: All of them we refer actually; all the femur we refer them.

**I: All of them?**

R: Yes.

**I: All the femur cases you refer?**

R: Yes.

**I: But tibia, you refer some or…**

R: We refer some but some are not referred. The complicated; we have to refer the complicated ones. There are those who can need [area? 06:59] is it

**I: Pardon?**

R: There are those tibia which need [area? 07:02] or something like that.

**I: Yeah, those ones you manage here?**

R: Yeah, some if they are able to buy those things for orthopaedic, they can be managed here.

**I: But otherwise they can be referred as well?**

R: Mhhh.

**I: Since the referral guideline came, is there any change in pattern; did you find that now you are referring less. What is that crossive referral; is it different? Because referrals [inaudible 07:31] on 1^st^ of July, are you aware?**

R: Yes I am aware.

**I: Is there an impact on that, does it…?**

R: Yes there is an impact because if we are referring for example a fracture femur, Dr Rona the consultant has to call Kenyatta.

**I: Has to call?**

R: Yeah, Kenyatta.

**I: Aha.**

R: And then the patient is accepted and we indicate the name as a patient. Like recently we had a fracture femur, I informed Dr Rona, called Kenyatta…We are not picking the phone actually, I talked to Dr Rona, called Kenyatta, he talked to the consultant from Kenyatta that is Dr Munene and gave the consent to refer the patient. So the patient went to Kenyatta.

**I: Went to Kenyatta?**

R: Yes.

**I: So that is the process nowadays?**

R: Yeah, confirm with consultant.

**I: So nowadays you don’t refer the way you used to refer?**

R: Anyhow?

**I: Yes**

R: You have to call.

**I: You have to call?**

R: Yes.

**I: And the consultant must call?**

R: Yes, a consultant must call?

**I: MO does not call?**

R: Sometimes the consultant can give a mandate to the consulting MO to call, sometimes yes.

**I: It’s either a consultant or the MO calls?**

R: Yeah.

**I: KNH.**

R: Yes.

**I: For orthopaedic admission?**

R: Yes.

**I: Orthopaedic referrals?**

R: Yes.

**I: So, that is what has changed?**

R: Yes.

**I: But in terms of numbers has it really changed? Do you find that you are referring less compared to…**

R: Yes, less because some are able to be managed here.

**I: What has changed that has made you to refer less? I’m comparing before 1^st^ of July and after 1^st^.**

R: I can say they are not many

**I: Okay, and are there any guidelines; are there any guidelines that you were given by KNH saying these are referral guidelines?**

R: I was not here when we got it, because like I said I’m one week old here. I have not seen it actually but I can ask Dr Rona.

**I: It must be verbal.**

R: Pardon?

**I: Is verbal?**

R: Yes.

**I: It’s a verbal guidelines?**

R: Mhh.

**I: So they are saying they are not taking any more?**

R: Yes.

**I: You have not seen any difference in terms of how many cases you referred to KNH after the guidelines? Is there any difference, do you refer less femur, do you refer more children, is there any change?**

R: Nowadays we are not referring many femur…

**I; Pardon?**

R: Not many; not that many.

**I: Is it the same before and after?**

R: It’s different.

**I: What is different?**

R: Now there are not many patients now that we are referring.

**I: Aha.**

R: Yeah. Because definitely you have to call the…When we are referring the femur, we have to call the doctor; that is the consultant Dr Rona and give the history of the patient, you send the X-ray and he advices what to do.

**I: Aha.**

R: Yeah, if the patient is going to remain here or go to Kenyatta.

**I: The kind of patients that you refer, is there anything unique; is there changes on the types of patient you refer; the old, the young, the elderly. Is there change in the profile of the patients that you refer?**

R: Most we get the RTAs patients.

**I: Pardon?**

R: Road traffic accident patients

**I: The RTA?**

R: Yes.

**I: They are the ones who…**

R: We refer.

**I: And is there any change in terms of age, sex when they are coming to..**

R: Mostly they are men. The locality, mmm this Kangundo road and the other Outering road. Kangundo and Outering road.

**I: Are the majority?**

R: Are the majority.

**I: Patients from those areas?**

R: Yes.

**I: The common orthopaedic cases you refer to KNH you said are which ones?**

R: The common ones?

**I: Yes.**

R: Actually the fracture femurs.

**I: Fracture femur, and?**

R: Head Injury patients.

**I: And head injury?**

R: Yes.

**I: Why do you refer head injury patients?**

R: We have no CT scan machine here.

**I: You have no CT scan?**

R: Machine, yes and also neurosurgeon.

**I: No neurosurgeon?**

R: Mmhh

**I: No neurosurgeon here in this facility?**

R: Yes.

**I: So, Children, is there any change in pattern of children that you referring?**

R: Children will come mostly… There are even children, they will come mostly a fall from height.

**I: But you manage them from here, you don’t refer them?**

R: Some yeah.

**I: Children you don’t refer mostly?**

R: We manage them here but some we have to refer if they will need that CT scan.

**I: But will you say you refer most children?**

R: The?

**I: Do you refer most children?**

R: The ones with head injury yes, and the ones who are… But I’ve seen most children have got head injury, most of them yeah.

**I: With head injury?**

R: Mmhh.

**I: But others…**

R: Fall from the height, maybe 4^th^ floor, 3^rd^ floor. And maybe others motor vehicle accidents.

**I: So, fall from a height is…Do you find fall from height being a common thing?**

R: Very much, yeah.

**I: Is it common than RTA?**

R: Yes to children now

**I: Children…**

R: Fall from height, not a…

**I: Fall from…**

R: Height.

**I: Height.**

R: Yeah.

**I: Is common.**

R: Yeah, than RTA.

**I: RTAs.**

R: Yes. RTAs we get mostly from the motor vehicles; they were knocked by moto vehicles…Motorcycle sorry. Motorcycle yeah.

**I: RTAs are…**

R: Mostly from motorcycle.

**I: And for adults is the same motorcycle…**

R: Motorcycles or the other; also vehicles there.

**I: Motorcycles?**

R: Motorcycles.

**I: For adults?**

R: For adults yeah.

**I: Adults also motorcycles?**

R: Yeah, and also other vehicles. And others knocked by a moving…

**I: By vehicles?**

R: Yes.

**I: So, the main reasons you are referring to KNH you have mentioned is lack of neurosurgeon?**

R: Mmhh.

**I: Pardon?**

R: Mmhh.

**I: Lack of neurosurgeon, lack of bed space…**

R: Bed space yeah.

**I: What else? You talked of equipment I think…**

R: Equipment also yes.

**I; Especially for…**

R: Orthopaedic case.

**I: Femur cases.**

R: Mmhh.

**I: What other reasons?**

R: Those are the main reasons actually.

**I: What of the patient prefer…**

R: Pardon?

**I: Do patients prefer to tell you to just “Take me to…”**

R: Yeah, those who have medical cover yeah.

**I: Pardon?**

R: Those who have got medical covers, they would wish to go to a hospital of their choices.

**I: Patients wish to go to…**

R: Hospital of their choice.

**I: To go to hospital of choice?**

R: Yes.

**I: So hospital of choice, which one is this, is it KNH?**

R: Not KNH, there are those who would wish to go to Kikuyu, what do we call it KU; there’s KU, Kikuyu, Kijabe, even others want to go to Agha Khan or other hospitals.

**I; Those with medical covers…**

R: Yeah, with covers.

**I: They actually opt to go out, but not to KNH?**

R: Not to KNH.

**I: Basically they go to private facilities?**

R: Private yes.

**I: Among these; bed space, neurosurgeon, equipment, patient preference, which one is the most common reason for them to…Most common reason why you are referring patients to Kenyatta?**

R: The most common reason?

**I: Yes.**

R: One is lack of bed space. Have you gone to our ward?

**I: No.**

R: I wish you go and visit there right now, any medical ward. You will see they are sharing two, two even I don’t know…

**I: Small space?**

R: Yes, the space is small.

**I: Neurosurgeon to the second, like that?**

R; Mmhh.

**I: This could be…Neurosurgeon, could be two?**

R: Yeah.

**I; Something like that?**

R: Yes.

**I: Have this changed with the referral guidelines; have things changed? Or is it the same reasons that you were referring before, are the same reasons you are referring now?**

R: Recently they expanded the ward; you have seen the new building there, they are expanding the ward whereby we are able to at least admit more patients in those wards.

**I: Is it orthopaedic?**

R: Not orthopaedic, no. there is no orthopaedic ward here

**I: There is no orthopaedic ward?**

R: No, not at all.

**I: Huh?**

R: No.

**I: There is no orthopaedic ward here?**

R: No.

**I: So when you say…**

R: They are calling it surgical ward we are sharing with orthopaedic.

**I: So, there is no orthopaedic?**

R: Orthopaedic at all.

**I: So they….**

R: They share it; they call it the surgical that is where they take the patient.

**I: They share…**

R: The surgical cases.

**I: Surgical ward with orthopaedic cases.**

R: Mmhh. And the same ward we have the medical cases for the patients.

**I: It’s one ward for male?**

R: Yeah, one general ward.

**I: Only one general male ward?**

R: Mmhh.

**I: For both surgical…**

R: And medical cases.

**I: And medical cases?**

R: Mmhh.

**I: So if a surgeon comes to the ground, he picks his patients…**

R: Yes.

**I: And goes.**

R: Mmhh.

**I: So then who limits patients to come…Who limits orthopaedic cases and says “Now we are not admitting orthopaedic cases”?**

R: There is no limitation because I could be having an emergency for a medical patient, only to be admitted and the [inaudible 18:31] to go and take that bed.

**I; So, if an orthopaedic case comes you just refer because there is no enough space?**

R: Mmhh.

**I: Are there a number of beds located to orthopaedic, number for surgical, number for medical?**

R: That one unless I ask, but before they had. Before there was 5 bed space for orthopaedic; before I left. There was 5 bed space for orthopaedic and then there was I think 5 for surgical and then 10 for…Before.

**I: 10 for medical. And that applies also for female side?**

R: Yes.

**I: Same?**

R: But they opened a new ward now so we have no problem with the bed capacity at the moment.

**I: A new ward has…**

R: Ward yeah opened.

**I: When?**

R: For both male and female

**I; When?**

R: Mmhh, when was it opened?

**I: This year or last year?**

R: I think last year; late last year.

**I; Late last year?**

R: Mmhh.

**I: So, the bed space has increased?**

R: Mmhh, but we are getting more patients who are very sick

**I: So…**

R: Like now I’ve got a patient who has to [inaudible 19:45] to be done, the bed made. I’ve not gotten space yet.

**I: Now, because of this increased space, do you think there is less patients being referred; now that you have more space?**

R: More space for? For the…Bed for the…

**I: Yes orthopaedic cases, because now they will have to share; orthopaedic will also have to get more…**

R: Yes.

**I; so do you think because of that now…**

R: Also the surrounding, because the surrounding we have so many patients who get sick. The surrounding; we are overpopulated. Patients are so many that we normally see and when they go to the ward, during the time of discharge, we can also tell they cannot afford this and this to pay medical bill, so it’s a challenge.

**I: Discharging?**

R: Yes.

**I: But then because of increased bed space, do you expect less referrals?**

R: Yeah, they should.

**I: But do you have less referrals?**

R: At the moment?

**I: Yes.**

R: I’m telling you that I’ve not gotten a lot of referrals at the moment

**I: So no…**

R: Since I came, that was last week Wednesday, so fare we have referred only two orthopaedic cases male. Since I came from last week Wednesday.

**I: To KNH?**

R: Yes, to KNH yeah.

**I: Only two?**

R: Yes.

**I: So, most of them are being admitted here?**

R: I’ve not gotten the number for…Orthopaedic I’ve not gotten so far, I’ve not gotten many of them for now.

**I: For admission?**

R: Yes.

**I: So, I may need to ask Dr Rono?**

R: Yes, are you with him at the seminar?

**I: No, I’ve not seen him.**

R: He is supposed to come.

**I: He is supposed to come?**

R: Yes, he is the right person you could have asked these questions. He usually knows.

**I: Maybe I can talk to him on phone, do you have his number?**

R: Yes

**I: You can give me his number then I can talk to him to shade a few light. I’ll talk to him on phone and say I’ve spoken to you then I’ll ask him to clarify a few points.**

R: Yes it’s important. You can talk to him before you leave.

**I: Yes. So any recommendations you suggest so that we streamline these referral thing to Kenyatta?**

R: Recommendation on our side?

**I: [crosstalk 22:39] to recommendation that you would suggest.**

R: Kenyatta when you call them, if you have patients for referral, they should be accepting those patients actually, especially the like of femur fracture.

**I: Is there a time they refuse?**

R: Okay, there was a time…Not even refusing, they even don’t pick the phone. And if there are more calls, they just tell your consultant to call.

**I: They insist the consultant to call?**

R: Mmhh.

**I: Not anybody else?**

R: Mmhh.

**I: Any other thing?**

R: Only for the orthopaedic cases yeah. I think for children it is okay; for children they are very much fast, that’s why I recommend them for being fast.

**I: They are accept…**

R: The children.

**I: Children referrals?**

R: Mmhh, very fast.

**I: Very fast?**

R: Mmhh.

**I: Anything else?**

R: If they go with Thomas splints, how are we supposed to get them back?

**I: Oh, Thomas splints?**

R: Mmhh because sometimes most of them are lying in Kenyatta.

**I: Ways to get the splints back?**

R: Mmhh.

**I: I think that we need to discuss with Rono and see how does he want to address it with Munene or Maina.**

R: Mmhh…Yeah, how we can get them back.

**I: Yeah, because they are actually colleagues with Maina and Munene [Inaudible 24:35] at the same time. So I’ll ask him to check on that.**

R: Mmhh.

**I; Any other thing about this referrals; anything you want to say about the referrals?**

[Silence]

**I: Is there anything that KNH can do to reduce the number of referrals that is taken to KNH?**

R: Basically we get enough bed space. If we can get enough bed space and have the orthopaedic consultant here, there is no much challenges we could be having actually.

**I: You want more bed space?**

R: Mmhh.

**I: But the equipment are there?**

R: Dr Rono can order whatever he needs…

**I: Pardon?**

R: Dr Rono can order them maybe from [inaudible 25:22] whatever he needs and even Kenyatta we can give some few.

**I: The main thing is the bed space?**

R: Yes.

**I: You don’t have issues with the theatre space?**

R: Theatre not really.

**I: No, it is not…**

R: Because in theatre, there is allocation for theatre; medical cases, surgery cases, there is allocation for theatre, orthopaedic, yeah.

**I: But if you are referring very many, you don’t have much to do.**

R: Sure.

**I; Any other thing that you would want us to tell about this issue of referrals? Do you think the system is working better? Are you happy with it the way it is; the referral system?**

R: Maybe you give me a month’s time and ask me the feedback; after…yeah.

**I; Apart from you, is there any person who knows, Rono should be knowing?**

R: Very much.

**I; Very much?**

R: Mmhh.

**I: I would like to call him on the phone and see what…Thank you very much, do you have any other comments to make?**

R: No.

**I: Okay, so at the end of this, I will…Because I’m doing in about 8 facilities. I’ve done…This is the first facility I am doing. I’m trying to see if I can go to Ngong tomorrow and see…**

R: Ngong there is a hospital? Which one?

**I: Ngon’g Sub-County hospital.**

R: Oh, sorry.

**I; Yeah, so I need to go there, I will also go to KU, I need to go to Thika. I will go to Mbagathi next week Tuesday.**

R: If you are to refer patients to KU, you have to ask for deposit first.

**I: Who asks for deposit, KU guys?**

R: Yeah.

**I: That’s why you are saying that patient must have a medical cover if they go there?**

R: Yes.

**I: I will go around and once we get the feedback, we consolidate, we will organize a designation where we shall share the findings.**

R: The better.

**I: And the recommendations. I will also make a point of talking to Dr Rono to get more of this and then I will compile. In fact today before I leave I will talk to him to and he will give me more information about the change in department and will include with whatever you have said, I will compile a report. So, I’m hoping to compile a report in the next three weeks because I still have to go to six others. This is the first, I have done two facilities; I have six facilities to go. I need to go to all of them and then do a report and then share with you.**

R: In Thika I know there is no problem because I Know they have an orthopaedic ward. In Thika I know they have an orthopaedic ward.

**I: Do you know anybody in Thika?**

R: In Thika?

**I: Yeah.**
