# Supplementary material for: Patterns of facility and patient related factors to the orthopedic and trauma admissions at the Kenyatta National Hospital: A qualitative assessment
Source: PLOS Glob Public Health. 2024 Jan 25;4(1):e0002323. doi: 10.1371/journal.pgph.0002323 (PMC10810445; doi:10.1371/journal.pgph.0002323)
Supplement: S1 File — (ZIP) [file pgph.0002323.s006.zip › KII TRANSCRIPTS/MBAGATHI HOSPITAL DR AGUNDA KII.docx]

| **FACILITY** | **MBAGATHI HOSPITAL DR AGUNDA** |
| --- | --- |
| **INTERVIEWER** | **Dr Maxwell Omondi** |
| **TRANSCRIBER** | **Dora Bloch** |

R: The cases for the surgery there, the patients buy the implants; the patients who cannot raise the implants mainly.

**I: The ones who cannot buy implants?**

R: Yeah.

**I: Which kind…**

R: In short patients who don’t have money or NHIF, or even have NHIF but no money to buy the implant.

**I: Either no money or no cash to buy implants.**

R: That is the main indication for the referral.

**I: In the process, which kind of fractures you end up referring to KNH?**

R: Any as long as they can’t buy implant.

**I: Is there any characteristic of these patients that you’ve noted; either they are male, female or particular…**

R: Mainly they are straight people.

**I: They are straight?**

R: Or some of them come…Most of the patients there come from Kibera or Kawangware and if they don’t have any employment, they may not be able to buy the implant to fix their fractures. And then you know the hospital some time back was so small it couldn’t accommodate any one…Initially our criteria or admission was a person with implant we admit, we operate after 1,2, 3 days they go home. Because there is no space number one, number two we don’t have traction beds. So it wouldn’t be fair to keep a femur fracture there who doesn’t have any implant.

**I: So no traction bed, you don’t keep…Patient…**

R: You know you can’t just keep a patient with for example a fracture femur on a bed without a traction. There are no traction facilities there.

**I: I think that also covers the next question which already you have mentioned; is the reason for referral…**

R: When you look at the answer I have given you, it should be able to cover all the questions you have.

**I: Yeah**

R: Because the main criteria for referral was that.

**I: The cost**

R: In ability to raise the implant. And again, if we have a patient that we think will require ICU care after operation. Mostly they are not many who need ICU but in the event that we get any like that, then you may have to refer. But the main indication for referral is in a bid to afford the implant.

**I: And patient preference; is patient preference an issue?**

R: Patients who prefer to go to Kenyatta they don’t come to Mbagathi. So patient preference is not one of them.

**I: Is not one of them. So anything else you would like to add regarding recommendations you would want to make?**

R: Recommendations concerning what?

**I: The orthopaedic cases, the referrals that we are having and how do we mitigate this?**

R: If you worked in government before, you will realise that government does not buy implants. That is the only way they can help; if they were to buy implants, then the need for referral won’t be there.

**I: Okay.**

R: I hope you have been able to summarize well?

**I: I will figure out how to fill in the other because of the time issue.**

R: In case you need a clarification just call me again.

**I; Okay,**

R: The hospital was initially called IDH; infectious disease hospital. It wasn’t oriented for surgery. But later I think Italians came and built theatre now even they are doing general cases. Orthopaedic surgery wasn’t much. But since the theatre was built, there were no implants and even the orthopaedic sets are not there. We are able to do because the people that sell implants come with the set. But the patient pays for the implant and they go back with their set. That arrangement works well when the patient is able to afford the implant. So, a patient who is not able to afford the implant, we cannot help because even if you have a set without implant, of course you will not help.

**I: Okay**

R: Traction table is available, II is available, what else?

**I: The traction table is not available, implants are not available, the orthopaedic sets are not available.**

R: No, the traction table is available

**I; It’s available?**

R: Image intensifier is also available

**I: And II is also available?**

R: Yes.

**I: CT-Scan; the spine cases?**

R: CT-Scan is not available.

**I: CT-Scan is not available?**

R: Spine if we are to do, again most of our patients can’t afford the [inaudible 05:48] in which case we would refer. But those who can afford, CT-scan is not available, they do it outside and then we can proceed and do.

**I: Okay, CT-Scan and MRI are not available?**

R: Those ones are not available. I don’t know why they were not brought because this image intensifier; the project they were carrying out in Counties, and they putting up IIs and CT-Scan. I don’t know why they didn’t put it up here. But they are not there.

**I: Which means if a patients wants, he will go and do an MRI or go to another facility all together?**

R: Yes, if we need them.

**I: We need them.**

R: Yes.

**I: Thanks so much, if I need… And the pelvic things you do? The pelvic fractures?**

R: There is no fracture that we do not do, as long as the patient will afford the implant or if it’s set, whatever we need.

**I: Okay, thanks.**

R: You are welcome.
