# Supplementary material for: Patterns of facility and patient related factors to the orthopedic and trauma admissions at the Kenyatta National Hospital: A qualitative assessment
Source: PLOS Glob Public Health. 2024 Jan 25;4(1):e0002323. doi: 10.1371/journal.pgph.0002323 (PMC10810445; doi:10.1371/journal.pgph.0002323)
Supplement: S1 File — (ZIP) [file pgph.0002323.s006.zip › KII TRANSCRIPTS/MBAGATHI HOSPITAL MUSA REFERRAL NURSE KII.docx]

| **FACILITY** | **MBAGATHI HOSPITAL MUSA REFERRAL NURSE** |
| --- | --- |
| **INTERVIEWER** | **Dr Maxwell Omondi** |
| **TRANSCRIBER** | **Dora Bloch** |

**I: As I’ve told you my name is Dr Omondi, this is Mbagathi Referral hospital. This is Musa, in charge…**

R: Of referral.

**I: Of referral.**

R: Yes.

**I: In Mbagathi.**

R: Yes.

**I: For how long have you been here?**

R: Since 2003.

**I: Been here since,**

R: 2003.

**I: 2003. So, now one of the things that…These are the things that [background noise 1:00] KNH came up with a referral guide on 1^st^ of July last year, that there will be no more referrals; walk in patients from all over the facility around, the…**

R: County and country.

**I: Yeah county and country. They wanted to be a true referral facility where they receive cases of patients who cannot be managed by other facilities around. That was 1^st^ of July. This study seek to understand whether that guideline that was put in place has influenced the nature of referrals to Kenyatta with regard to orthopaedic cases. There are few cases that will… The whole thing will take maximum 30 minutes. So, one of the things I wanted to know where do you refer your orthopaedic cases mostly?**

R: We refer to KNH

**I: You refer to KNH?**

R: Yes.

**I: Which other facilities do you refer to?**

R: It is only KNH; that is the nearest.

**I: Only KNH?**

R: Yes.

**I: It’s the nearest?**

R: Yes.

**I; The nearest health facility. Are you aware about this guideline from KNH?**

R: We are aware of the guideline and we do have our own protocol also and this is the protocol you can take a photo of it.

**I: This is the referral protocol?**

R: Yes, for all cases.

**I: Let me see. So this is the referral protocol; upon decision to refer patient by the team…So it’s the medical officer who calls, not a nurse?**

R: Yes, the medical officer.

**I: The nursing officer informs the nurse covering to prepare the ambulance, nursing officer prepare the patient, documentation is done at the referral register, patient notified the decision is made and actual referral is done. The patient is referred accompanied by the nursing officer. So, this is the referral protocol.**

R: Yes.

**I: So the medical officer is the one to authorize?**

R: To initiate that referral.

**I: The nursing staff executes?**

R: Yes, there is a caveat now that the consultant has to be informed of that decision.

**I: As a referral protocol initiated by the MO**

R: By the medical officer.

**I: Either an intern or anyone?**

R: No, it’s the medical officer, not intern.

**I: Medical officer?**

R: Medical officer.

**I; Qualified.**

R: Qualified.

**I: Registered medical officer in consultation with the consultant.**

R: Yes.

**I: When was this enforced?**

R: It has been enforced I think since almost 3 year; 3 to 4 years

**I: The protocol is now 3 years.**

R: Yes.

**I: Since it has been enforced.**

R: Yes.

**I: How does that intersect with the guideline from Kenyatta?**

R: I have not seen the one for Kenyatta, but every institution has to streamline its protocol towards; to be in line with the one for Kenyatta.

**I: Do you know…have you seen the one for Kenyatta?**

R: I don’t think if I’ve seen it

**I: The circular, did you notice…**

R: There is a circular yes.

**I; Do you have it here?**

R: No, I don’t see it here.

**I: Where did you see it?**

R: There is a circular the time we had a meeting at the board room and the Kenyatta guys came and addressed us. I think they discussed at the higher level with the medical superintendent, but for me I did not see it actually. But I know there is a circular.

**I: There is a circular.**

R: Yes.

**I: But you’ve not seen it.**

R: I have not seen it.

**I: Do you know what the circular says?**

R: To the effect of the referral of patients, the final person who to make a decision is a consultant.

**I: The consultant makes the final decision?**

R: The person who gives that green light; now it is okay you can go ahead.

**I: And initiate the referral?**

R: No, I’ve told you it’s the MO who initiates, but has to consult first.

**I; Consult.**

R: Yes.

**I: Apart from concurrent with the consultant…**

R: Brief the consultant, if the consultant is in agreement, then the medical officer goes ahead. You cannot refer without the knowledge of the consultant.

**I: That is how it is?**

R: Yes.

**I: That is how it was with effect from July?**

R: Yes.

**I: So, from 1^st^ July, consultant for referral approval.**

R: Mmhh.

**I: Without that, no**

R: You cannot refer.

**I: And that should be documented?**

R: Should be documented.

**I: That consultant so and so has agreed patient to be referred to hospital of choice.**

R: Yes.

**I: Or can be referred to KNH; whichever decision has been agreed on?**

R: Mmhh.

**I: That has been the main different from the 1^st^ of July; involvement of consultant?**

R: Yes.

**I: Involvement of consultant has been the main change. Regarding to referrals. What kind of patients orthopaedic; I’m looking at orthopaedic cases do you refer to Kenyatta?**

R: Those are the multiple fractures that we call open reductions.

**I: Multiple fractures that require…And reduction…**

R: Yes. Mostly those are the cases.

[Interruption]

R: So this is a term based on RTA; as a result of RTAs, because most cases are as a result of RTAs. So it involves fractures; internal fractures. So, those are the types of cases that [inaudible 07:52]

**I: What kind of fractures?**

R: Fractured femur

**I: Commonly?**

R: Yes.

**I: Fracture femur.**

R: Most is fracture femur, I’ve not seen head…Mostly it involves the fracture of the femur

**I: Most of the fractures are femur…**

R: Yes, most is femur.

**I: Femur?**

R: Mmhh.

**I: Tibia**

R: No, tibia it is normally served outpatient; tibia fibula you just align it on [inaudible 08:26]. But if it complicated, it’s referred.

**I: But mostly it’s femur fracture?**

R: Yes.

**I: Those are the kinds of fractures that you actually refer; femur fractures?**

R: Yes.

**I: Is it because you don’t have the implants?**

R; First of all we don’t have the capability of buying those resources…We don’t have the resources. Also, the materials such as the orthopaedic beds.

**I: Orthopaedic beds**

R: Not available completely.

**I: Not available?**

R: Yes.

**I: And they cannot afford?**

R: And they cannot afford, but being told to buy all those things, they cannot.

**I; And NHIF**

R: NHIF, most of our catchment area, they cannot even afford the NHIF cover.

**I: The catchment population…**

R: Because they are coming from Kibra…

**I: The catchment population cannot afford NHIF cover.**

R: Yes.

**I: Because they come from Kibra…**

R: And those low income.

**I: Low income…**

R: Areas.

**I: Areas. So they can’t afford…Most of them have no NHIF?**

R: Most of our patients; not only orthopaedic, but most, the majority of our patients…

**I: Have no NHIF?**

R: Yes.

**I: So, they have no NHIF.**

R: Yes.

**I: That becomes difficult; that means you have to refer?**

R: Yes.

**I: So any fracture, basically most of them you can’t manage?**

R: Not any fracture but mostly the femur; fractures are being managed in our…

**I: The one for the forearm; radio [inaudible 10:14]**

R: It is managed in the casualty, we have a trauma…

**I: No, the ones that require to be fixed a plate?**

R: No, it has to be referred.

**I: It has to be referred?**

R: Because if the patient cannot afford and the hospital does not have those plates, there is no point of staying with the patient…

**I: When you have no implant.**

R: That will be bordering negligence

**I: So here you have no at all any implant?**

R: We have no implant at all

**I: At all?**

R: At all.

**I: You have no implant, you have not orthopaedic set?**

R: Yes; orthopaedic set could be there, but just the basic ones; the basic set. But the implants specifically the k-nails and the like, the patient has to buy which of cause most of them just give up.

**I; They give up.**

R: Yes.

**I: They don’t have money, they don’t have NHIF.**

R: They don’t have NHIF; they can’t afford NHIF.

**I: But for those who have NHIF you can fix?**

R: Yes, if they have it, it’s okay but the problem is when it comes to buying of those implants…

**I: NHIF doesn’t cover?**

R: 40, 000, where will they get that resource from?

**I: NHIF can cover that?**

R: I’m not sure if NHIF can cover that amount, but I have not seen them covering. I’ve not had such an experience of which NHIF covering that procedure.

**I: Children; are children also involved?**

R: Yeah, children are involved in fractures…

**I: Do you refer them?**

R: No, I can’t remember a child that was referred, because most of their fractures are managed in trauma department. It is mostly adults, but for children, I have not seen; I have not seen them being referred.

**I: Apart from the fracture femur which we’ve talked about, you also refer…What other types of fracture do you refer apart from the femur? What of politrauma patients; patients who have head injury**

R: In such cases, that one we do refer definitely; head injuries are referred To KNH. Of course this depends with where the patient is coming from because what we do inform the referring facility is to do a CT-scan; come with CT-scan ready and then it be reviewed, the patient will be reviewed…

**I: With the CT-Scan?**

R: Yes, in [inaudible 12:54] with the consultant.

**I: If the patient is being referred to Mbagathi, you always recommend to come with a CT-scan?**

R: Yes, currently you come with a CT-scan because we don’t have the CT scan. Yes. And again to save on that time

**I: So, patients referred here to come with CT-scans.**

R: Mmhh.

**I: Because you have no CT-Scan?**

R: We have no CT-Scan.

**I; So that to save on time.**

R: Yes

**I: On time and decision on management.**

R: Yes.

**I; Paediatric, generally you don’t because…**

R: Mostly managed in trauma department. I have not seen a cases being referred to KNH from peads.

**I: You have not seen cases being referred from peads?**

R: No

**I: How do they manage with the plasters?**

R: They do manipulate and they do that reduction manually; of course under sedation in casualty.

**I: In casualty?**

R: Yes.

**I: What are the common orthopaedic trauma cases that you refer? I think that’s what you’ve talked about. You mentioned the…**

R: Fracture femur, that we carry open reduction, and head injury

**I: Head injury. Basically trauma cases.**

R: Mmhh.

**I: With head injury.**

R: Yes.

**I: Those are the ones that you refer; those are the ones that you end up…**

R: Those are the common ones that we do refer.

**I; Pelvic, spine?**

R: I’ve not heard of…It’s very rare; I think I’ve just had one case of a fractured pelvis, that I think was last year. But it is not common, and of course this was an accident; a motorcycle guy. He had a fractured pelvis and was referred to KNH.

**I: Do you manage pelvic fractures here?**

R: No, he just came to casualty, was reviewed by a consultant and referred to KNH.

**I; Referred to KNH?**

R: Yes.

**I: So, pelvic fractures you also refer?**

R: Yes.

**I: And spine?**

R: Very rare but we had a spine I think also last year; a fall from height, it was referred to I think KNH, yeah KNH.

**I: Even the spine?**

R: Yeah, spinal cord injury was referred.

**I; So, even spines you don’t manage here?**

R: No, we don’t manage

**I: But they rarely come; mostly the ones that come here are femurs, head injury.**

R: Femur and head injury.

**I: And once you get them, you refer straight to Kenyatta?**

R: Yes, because of that gap, we have to refer very fast.

**I: You have to act very fast.**

R: Yes.

**I: The patients who come, are they mostly female or male?**

R: They are mostly male.

**I; Mostly male?**

R: yes.

**I: You say what %; 40% maybe 60?**

R: They are all male.

**I: So all % is make?**

R: Yes.

**I: And adults?**

R: They are adults.

**I: What age bracket?**

R: Between 35 to 45

**I: And they are usually RTA?**

R: Mostly RTAs; motorcycle.

**I: Motorcycle?**

R: Yes, motorcycles.

**I: Accidents?**

R: Yes.

**I: Mostly?**

R: Mostly.

**I: The motorcycles?**

R: Yes.

**I: Any other thing in terms f patients apart from the fact that they are poor, poor income?**

R: Low economic status. That should be the main cause as to why they cannot afford the NHIF, that is the main cause.

**I: And so what factors are associated with the referral; what is the main reason that you refer?**

R: For further management.

**I: What do you mean further management?**

R: For example the open reduction of the fractures, insertion of those plates…

**I: Lack of plates?**

R: Yes.

**I: Implants?**

R: Yes.

**I: Plates, why else do you refer?**

R: Lack of orthopaedic beds.

**I: No orthopaedic beds.**

R: I think that’s all; those are the only things, the main ones.

**I: Do you refer because of expertise?**

R: No, we do have an orthopaedic surgeon.

**I: You have… okay, bed space, is that a reason why sometimes you refer?**

R: No.

**I; Have enough bed space.**

R: yes.

**I: What of issues to do with patients preference; do you have situations where a patient will prefer to go to Kenyatta? The patient has been brought here but the patient prefers to go to Kenyatta?**

R: We rarely… In fact it’s the opposite; they are reluctant to go to Kenyatta. They are very reluctant, I don’t know; it is phenomenal, I don’t know why they are reluctant to go to Kenyatta. It takes a lot of persuasion.

**I: A lot of persuasion?**

R: Yes, a lot of explanation as to why they have to go to Kenyatta. Even the relatives have to be involved to educate them there is no benefit for the patient staying around here; we don’t have enough resources that is why you are being referred to KNH. But mostly they are so reluctant to go to KNH.

**I: They are reluctant to go to KNH>**

R: Yes.

**I: Any recommendations you would like to make regarding this referrals; what can be done to minimize referrals, to make sure patients are managed here and also to smoothen that referral process if there are any hitches around it?**

R: The first is to improve on the infrastructure; that is the main thing. To improve on the infrastructure whereby we do have the resources.

**I: When you say infrastructure what do you mean?**

R: We need those orthopaedic section, orthopaedic ward…

**I; Orthopaedic ward?**

R: Yes, with resources.

**I: With resources?**

R: Yes.

**I: Like what; which resources?**

R: The ones we are missing like plates and the like.

**I: Implants?**

R: Implants.

**I: You want them to be available?**

R: Yes. I think that is the main challenge.

**I: Oh, that is the main…**

R: Yes, the main challenge.

**I: What of the CT-Scan?**

R: CT-Scan yes, those are also important; the CT-Scan and all that. CT-Scan is important.

**I: But the main thing is the implant; you need to have implants…**

R: Implants and the infrastructure itself.

**I: When you talk of the infrastructure…**

R: The art section during orthopaedic such cases.

**I: So you need a separate orthopaedic ward?**

R: Yes.

**I: designated…**

R: Designated specifically for orthopaedic.

**I: section, with all the equipment.**

R: Yes.

**I: Orthopaedic beds**

R: Orthopaedic beds, CT-Scan

**I: MRIs.**

R: MRIs.

**I: Anything else you would like to comment? How is that referral process, it’s smooth?**

R: Yes, there is no problem with the…Okay the only challenge with the referral process, we only have little number of ambulance operator. At times you might find we really need this and the ambulance operator is not available; has been assigned other duties. So, there is that delay.

**I; You have only one ambulance?**

R: Yes.

**I: One ambulance or one operator?**

R: We have one ambulance and the ambulance operator is the one who will run errands at the same time refer the patients to KNH.

**I: Delays.**

R: Yes, definitely it will lead to delays.

**I: You need to have more ambulance.**

R: More drivers; more ambulance operators.

**I: But ambulance, how many do you have?**

R: We do have from NMS; we are being assisted by the NMS. There are many ambulances around…

**I: It’s operators?**

R: The ambulance operators are the ones that are missing. We have the resources but the human resource is not adequate.

**I: But you have many ambulances?**

R: Yes.

**I; How many?**

R: I can’t say how many because they are under NMS.

**I: But they are easy available; they are here?**

R: Yes, there is one standby, but because of lack of the driver, that is why it is just on the parking lot. The driver is engaged in other errands.

**I: Which means you can access more ambulance?**

R: yes…

**I: The only issue is the ambulance operator, but accessing ambulance you can access?**

R: Yes.

**I: If there are more operators, you get more ambulances?**

R: yes. The ambulance are already there.

**I: Under NMS.**

R: Under NMS.

**I; Good, anything else you would like to talk about this referral thing?**

R: I think that is all.

**I: Any other observation, any other question you have?**

R: I have not seen any, because the challenges are just that process, implants. Those are the main challenges. I don’t think I’ve got any other that is unique; I’m not seeing any.

**I: If that is it, I think we can then end there, thank you so much for your time.**

R: No problem.

**I: If I have any questions, I’ll call you just to get more clarifications. So thank you so much.**

R: Okay.

**I: At the end of it, we will do a report and we will call you to share with you the feedback of what we have seen. Otherwise it has been great talking to you. It has been quiet informative. Thank you.**
