# Supplementary material for: Patterns of facility and patient related factors to the orthopedic and trauma admissions at the Kenyatta National Hospital: A qualitative assessment
Source: PLOS Glob Public Health. 2024 Jan 25;4(1):e0002323. doi: 10.1371/journal.pgph.0002323 (PMC10810445; doi:10.1371/journal.pgph.0002323)
Supplement: S1 File — (ZIP) [file pgph.0002323.s006.zip › KII TRANSCRIPTS/MWINGI COUNTY HOSPITAL.docx]

| **FACILITY** | **MWINGI COUNTY HOSPITAL** |
| --- | --- |
| **INTERVIEWER** | **Dr Maxwell Omondi** |
| **TRANSCRIBER** | **Dora Bloch** |

**I: I’m Dr Maxwell Omondi a registrar at Kenyatta National Hospital, orthopaedic surgery. I’m collecting data as part of the proposal…**

I: Data?

**I: Yeah, data. Just a key informant interview on orthopaedic referrals from Mwingi to Kenyatta National Hospital in the year 2021.**

R: 2021?

**I: Yes.**

R: Aha.

**I: I had spoken to Dr Mumo the Med Sup and he suggested that I talk to you because some information he didn’t know. He was not familiar with some information so he said I should talk to you. The main thing that I wanted to get, is to understand where do you refer most of your patients for orthopaedic?**

R: Actually we never refer.

**I: Yes?**

R: We never refer, we usually do here.

**I: But in the records that we have, we have patients who have been referred from Mwingi to Kenyatta last year.**

R: Last year?

**I: Yes**

R: Last year but one…

**I: Not last year but one.**

R: We used to do that with our general surgeon, although [inaudible 1:55] to refer to Machakos mostly.

**I: But our records…**

R: These other simple things like plating and nailing we used to…

**I: Sorry?**

R: We used to do them.

**I: But there are some patients who have been referred to Kenyatta last year.**

R: Last year?

**I: Yes, and that is what I…**

R: Unless there is patient request. Some of the Somalis here when they get accident, they don’t like being admitted here; they like going to Nairobi.

**I: The Somalis?**

R: So most of them is self-request.

**I: Most of them is self-request?**

R: Yes.

**I: Why do they prefer to…Why are they preferring to go to Nairobi? Is it Kenyatta or just any facility?**

R: Once you write patient request, we don t know where they take their patients. There are lots of Somali hospitals in town; the Arabs.

**I: You have got how many orthopaedic surgeons there in Mwingi?**

R: We have 1 now.

**I: Since when?**

R: Since last year

**I: End of last year or beginning of last year?**

R: Mid there.

**I: Mid last year?**

R: Yeah.

**I: And before that, where were the patients being managed?**

R: We used to have a general surgeon.

**I: Oh, you used to do the plating the femur…**

R: Yes, plating, nailing we used to do.

**I: And pelvic surgeries as well?**

R: Pelvic?

**I: Yes.**

R: No, those ones we used to refer to Machakos.

**I: You used to refer them to Machakos; pelvic?**

R: Yes.

**I: And the spine?**

R: Spine, in Nairobi.

**I: You refer to Nairobi?**

R: Kijabe or Kenyatta.

**I: Then there are still patients you refer to Kenyatta last year?**

R: Right now if we get spinal injury, we refer them.

**I: To Kenyatta?**

R: To Kenyatta; head injury, spinal injury.

**I: And head injury?**

R: Yes.

**I: Why do you refer them to Kenyatta?**

R: For those ones with severe cervical damage

**I: Severe cervical damage?**

R: Yes.

**I: Why do you refer those ones to Kenyatta?**

R: Pardon?

**I: Why do you refer those ones to Kenyatta?**

R: Because that’s where we have the neurosurgeons and so.

**I: You guys don’t have neurosurgeons yet?**

R: WE don’t have neurosurgeons.

**I: And ICU, do you have ICU capacity?**

R: Yeah, capacity we don’t have.

**I: You have?**

R: We don’t have.

**I: You don’t have ICU capacity?**

R: We don’t have.

**I: That is the reason…But Machakos have ICU?**

R: Machakos?

**I: Yes.**

R: Unless they created recently; they are level 5 now.

**I: They…okay.**

R: Yes, they have most of the facilities.

**I: Those types of patients you refer you said most of them are Somalis?**

R: No, unless those with simple fractures, the Somalis usually go on their own request. But those with severe damages like cervical, head injury, pelvic, we refer them to Nairobi because we have a neurosurgeon there, we have CT-Scan, we have MRI, but here we don’t have.

**I: So, there you also lack the CT-Scan, you also lack the MRI?**

R: We also lack CT-Scan, MRI, we refer them to Machakos or Nairobi imaging centre; it is just close there at Kenyatta in Nairobi. They go test and bring the results.

**I: They do the CT-Scan in Nairobi then…**

R: They do the scanning and then they come back.

**I: They come back?**

R: Yes, if they are not severe, but if they are severe we tell them they go do the scan as they move to Kenyatta.

**I: They go to Kenyatta?**

R: Yeah.

**I: the type of those, are they men, women, children; what is the kind of…**

R: Mostly women and men; I have never seen a child.

**I: They are usually…are they…**

R: They are usually men and women.

**I: Adults?**

R: Adults.

**I: They are usually adults?**

R: Adults.

**I: Rarely are they children.**

R: Children we usually get these simple fracture of climbing the trees looking for fruits and all that.

**I: Fall from height?**

R: Yes.

**I: When children are falling from height while harvesting fruits…**

R: Playing in school.

**I: Playing in school.**

R: Yes.

**I: Those ones you end up fixing them at Mwingi?**

R: Yeah, we fix them here at Mwingi.

**I: It’s only the adults who have got the severe injuries like crashed cervical; those are the ones you refer to Kenyatta?**

R: The ones we refer to Kenyatta.

**I: Are they mostly men or women?**

R: Unless I go back to the record, most of them are due to those road traffic accidents.

**I; Road traffic accidents?**

R: Yes, and it’s a combination; we can have a female or a male.

**I: The road traffic accident, is it motorcycle or vehicle accident?**

R: Both; vehicle and motorbike.

**I: Motorbike?**

R: Yes.

**I: In Mwingi, those long bone fractures you can fix, you don’t…**

R: long bone fractures we deal with them.

**I: You just refer the ones you have mentioned; severe…**

R: The ones I’ve mentioned are the ones we refer.

**I; Those politrauma and probably head injury…**

R: Politrauma depends where and what, because if we have fractures and then we have internal injuries, we usually…The general surgeon deals with internal injury; if it’s abdomen, he deals with that and then after that we deal with the bones later.

**I; So currently you have one ortho surgeon. And space, do you have issues with space in terms of theatre space at Mwingi?**

R: Theatre space, yeah evert department has one day.

**I: In theatre?**

R: Yeah, because we have two theatres, but there is one specifically for maternity. This other one we use for gynae, general surg, PMT. So we have one day each for each department.

**I: Is it enough, do you feel it’s enough; the one day, is it sufficient?**

R: No, it is not enough because at times you deal with…the general surg has some emergencies. We end up giving some time for the general surg to deal with their patients then we go later; even if his time was yesterday, if he gets an internal injury, we can’t tell him your theatre day was yesterday. So he has to go in and when he is through, we start ours. That is why yesterday when you were calling me, I had just brought the first patient and we had to actually wait to go in at 7:30.

**I: In the morning?**

R: Yes, then he got trapped at the abdomen. We had to give him time; he entered at 7 and we went in at 11.

**I; At 11?**

R: Yes, so those challenges of emergencies.

**I; And the implants, are you have issues with implants and…**

R: We don’t have issues with implants…

**I: You are well stoked?**

R: Although we don’t have at the hospitals, we usually prefer to the patients, direct them where to purchase in Nairobi and they usually get them in time. The issue is only finance.

**I: The implant issue; they buy, you have no major issue there?**

R: Yeah, we don’t have an issue.

**I: You can never refer a patient because of lack of implant?**

R: No.

**I: You don’t?**

R: We don’t.

**I: Do you SIAM; the II, the imaging?**

R: Yeah.

**I: You have SIAM?**

R: We have SIAM.

**I; You guys are well equipped.**

R: We are very well equipped.

**I: Traction table?**

R: Traction table we have.

**I; You have?**

R: We have.

**I; You also have orthopaedic beds?**

R: Yes, orthopaedic beds we have.

**I: You have some?**

R: We have

**I: Is there a special dedicated orthopaedic bed?**

R: No, they are mixed up with general surg.

**I; General surgery?**

R: Yeah, we divided room.

**I; You divide room?**

R: This one to this room and this one, like that.

**I: Are you aware about the KNH referral guideline that was enforced from 1^st^ of July 2021?**

R: July 2021?

**I: In July 2021, KNH came up with enforcement of a guideline that requires that Kenyatta remains a referral facility and for you to refer patients, you must first talk to Kenyatta for concurrence before they can allow you to refer patients there.**

R: Even before July, we usually don’t refer our patients to Kenyatta without…We usually call them before we confirm the vacancy; if they have space, then they give us a guideline they tell us you can now bring the patient. You just don’t refer like that and there is the ambulance.

**I: I think we are almost done, apart from the issues you have talked about that makes you refer patients to Kenyatta, you said when you have a pelvic fracture, you refer them to Machakos or Kenyatta?**

R: Pardon?

**I: Pelvic fracture.**

R: Pelvic we refer to Kenyatta.

**I; Pelvic you refer to Kenyatta?**

R: Yes.

**I: And also spine you refer to Kenyatta?**

R: To Kenyatta.

**I: Those severe injuries you refer them to Kenyatta.**

R: Yeah, those severe injuries and whatever.

**I: most of those cases, you end up referring them to Kenyatta instead of Kenyatta?**

R: Machakos they don’t have…Unless they have made an ICU, they never used to have it. Also they never used to have a CT-Scan, now they have and MRI.

**I: Are there scenarios where patients want you to refer them to Kenyatta? Are there scenarios that patients will just make a preference that they want you to refer them to Kenyatta specifically?**

R: Yeah, some patients request where to go; they tell you “Refer me to Kenyatta because my relatives are near there”

**I: Because their relatives are next to Kenyatta, so they prefer…**

R; Yes.

**I: So they prefer to be sorted from Kenyatta?**

R: Yes.

**I: Any recommendations that you can make; we are coming towards the end of the discussion, any recommendations you will make regarding referrals to Kenyatta?**

R: Recommendations?

**I: Yes.**

R: If I give you a brief history of what we have done, what caused the injury, our examination then the diagnosis, then we refer.

**I: Then you refer?**

R: Yes.

**I; Is there anything that you would require Kenyatta to do differently to improve the referral system?**

R: The referral system, we don’t go more in details like clinicians. After we refer, it’s for the nurse and the driver to take over.

**I: Your role is just to refer and then…**

R: Ours is only to write the referral letter.

**I: And then the nurses take over the whole process**

R: Kenyatta then takes over the whole process.

**I: What do you think…**

R: But we are the ones to call, not the nurses, we call tell Kenyatta we want to send this patient with this and this, they tell us if whoever is supposed to see the patient is around; if it’s a neurosurgeon and what time. That is alerting; there is a patient who is coming from Mwingi.

**I: What other things would you recommend so that you minimize on the referrals; is there anything that you suggest that can be done so that you don’t refer patients to Kenyatta; they can be managed within Mwingi?**

R: It’s only the improvement of the facility.

**I: Which aspects?**

R: Get the required equipment.

**I: Like which equipment?**

R: If it’s…Like CT Scan, MRI, we have an ICU built; that’s a factor thing, and then we get the required staffs; the specialists.

**I: The neurosurgeons.**

R: Yeah, neurosurgeons, [inaudible 16:48] those people, such specialists.

**I: What of the orthopaedic surgeons; the one you have can do pelvic fractures and spine fractures?**

R: Yes, if he gets these facilities, he’s okay.

**I: He’s trained already; on pelvic management and spine…**

R: Yes; not spine.

**I: Sorry?**

R: Spinal is more delicate.

**I: Sorry?**

R: Spine is supposed to be done by the neurosurgeon. But there are those spine, smaller ones than spine, he can deal with them.

**I: You don’t need an experienced orthopaedic surgeon to be there; to manage all…**

R: Being the first post from post graduate, also an experienced one is also needed; it’s a requirement. Experience is better than a teacher

**I: If you get someone who is experienced, can be able to manage most of those complex injuries, if you have the…**

R: I believe so.

**I: If you have the required equipment and facilities.**

R: If we have the required equipment and space.

**I: Anything else that you recommend?**

R: What?

**I: Anything else that you recommend to improve on the referrals and to also minimize on referring patients to Kenyatta?**

R: Our space is too limited because I’ve told you we have general surg and orthopaedic. if we can have orthopaedic department on its own, and theatre for the same, we would minimize referral.

**I: You will minimize referral?**

R: Yeah, because you find we are ever in on a daily basis and now with the current environment; with the motorbikes and also a lot of accidents on the road, and with one doing surgery daily in a week, it’s not enough.

**I: It’s not enough.**

R: It’s not enough. If we could be doing daily basis; from Monday to Friday, we would minimize those referrals

**I: You see Mwingi is on the highway, right?**

R: Yes, it’s on a highway.

**I: And there is a lot of injuries, road accidents on the highway.**

R; Injuries, road accidents.

**I: It is good if we can find a way to make it like a trauma centre.**

R: a trauma centre exactly.

**I; So that we can be able to manage cases as they come and…But even currently you don’t actually refer patients because of limited space. You are able to manage all cases?**

R: Yeah, we have limited space, we have shortage of specialists. Like now it’s on a weekend, now we will see them on Monday.

**I: You will see them on Monday?**

R: They will be managed by MOs over the weekend because we are not around.

**I: But still, even…**

R: When we go there on Monday, also Monday is not our theatre day; it is for another department. Those are some of the disadvantages we have.

**I: Your theatre day is on which day of the week?**

R: It’s on Friday.

**I: Friday?**

R: Yes.

**I: I would expect you to be referring many patients from Mwingi but it doesn’t seem that you refer many patients.**

R: Yes, because most of them; 99% is long bones.

**I: 99 % are long bone.**

R: If a patient has a fracture femur, they are first put on traction awaiting for the surgery.

**I: Oh, so they are put on traction then you give…if they don’t have the nail, you have to buy the nail?**

R: We put on traction awaiting for them to purchase the nails.

**I: As soon as they purchase the nail, you fix it?**

R: Immediately you purchase, we schedule that.

**I: 99% are long bones?**

R: they are long bones.

**I: The long bones that is femur…**

R: Femur, the upper limb.

**I: The upper limb. The tibia.**

R: The tibia.

**I: 99%.**

R: Yes

**I: There is no overwhelming; you don’t have a situation where you are not able to manage the case because of lack of theatre space?**

R; No.

**I: That means the workload for trauma in Mwingi is not too much; it’s not a lot?**

R: It is too much; at times we are caught up. Like last week but 1, actually the other department didn’t have a lot of work and we used to do daily; at least 2, 3.

**I: In a day?**

R: Yes, 2, 3 in a day.

**I: The official allocation is once per week but if the other theatres…**

R: Like yesterday I had around 6 patients; 6 cases.

**I: 6 cases?**

R: 6 cases. Of which we didn’t manage to do them all because of emergency of the general surgeon in the morning

**I: If you didn’t do, that means those patients…**

R: We did 4 up to around some minutes to 11 at night; PM. Then we postponed the others until Monday.

**I: Until Monday?**

R: Monday; Monday actually is our emergency day for all departments. If Mondays we don’t have other department with emergency, we usually go in for the balance left on Friday.

**I: That is good, that is useful information. Most patients who come to Mwingi their catchment is from which area? What is the catchment population for Mwingi for orthopaedic cases?**

R: the catchment is too big.

**I: It’s from where to where; from which…**

R: It starts from the boarder of Garissa and Mwingi.

**I: Boarder of Garissa and Mwingi.**

R: Garissa and Mwingi and the other far end of Syokuru, and then we come to the lower part of Machakos Kibioko and part of Kitui West and the whole of Kitui east.

**I: So you have got a huge catchment population?**

R: We have got a huge catchment population, although some population in some places is very minimal.

**I. Most of the patients who are having accidents, are they patients on transit to Garissa?**

R: Transit to Garissa, transit to Nairobi and also the periphery and the motorbikes of the other parts and the central town.

**I: Even the central town and periphery, are the majority the ones on transit or the local?**

R: From the local.

**I: From the local?**

R: Yeah from the local.

**I: So those ones who are on transit to Garissa, transit to Nairobi are not majority?**

R: They are not the majority; we may even last for almost…we get once, twice in a month we have traffic road accident of vehicles not motorbikes. Motorbikes is daily.

**I: Motorbikes are daily accidents?**

R: Daily; even if they don’t get fractures, they get soft tissue injuries.

**I: Soft tissue injuries?**

R: Mmhh.

**I: But these ones for traffic accidents it’s once in a while?**

R: Once in a while

**I: They are not common?**

R: They are not common.

**I: Are most of them having insurance cover; most of the patients who come to Mwingi?**

R: Most people here don’t have the knowledge of the covers and insurance.

**I: So they are cash payers?**

R; The others who come and will not be able to purchase the implant.

**I: What do you do with them?**

R: We advise them to go and have cover; go to NHIF they register and wait for 60 days, then they top up so it can work.

**I: How do you wait for 60 days with a fracture, the fracture will heal?**

R: [laughing] you [inaudible 26:57] the way it is.

**I: Because if…**

R: There are those who are not able at all, they have to organize for a fundraising.

**I: Fundraising?**

R: Yes, and it will take almost a month or two.

**I: If they wait for fundraising for fractures, the fracture will malunite my friend.**

R: If it malunites we go in and align.

**I: You ostumize and then align it again?**

R: Yes.

**I: So, most of the patients don’t have the NHIF cover?**

R: Yes, they don’t have NHIF cover

**I: They are just cash payers and the have to do fundraising things like that.**

R: This or they sell them cows.

**I: They sell them what?**

R: Cow, cows here have a lot of money.

**I; Oh, they sell cows.**

R: Yes.

**I: They sell cows then they go to buy the implants.**

R: You can have one bull at around 80,000.

**I: And that is enough.**

R: That is their farming here.

**I: That is Mwingi people.**

R: Farming is mostly raring cattle.

**I: That is for people in Mwingi?**

R: Mwingi. Actually you may see a layman but money is not an issue because of the cows and goats.

**I: Cows and goats.**

R: Yeah.

**I: That was useful. I think we’ve discussed quite a bit. You refer most of your patients to Kenyatta.**

R: Mostly to Kenyatta.

**I: Mostly to Kenyatta because Machakos doesn’t have the capacity.**

R: Unless those…There are those who tell us we want to have a discharge, we want to go to another hospital. They don’t even tell you where but you counsel them; you ask them, you pursue them to tell you so that they give proper answers. Because they might be going somewhere they won’t get the service.

**I: But for you guys the majority of the ones you refer, you actually refer them to Kenyatta?**

R: To Kenyatta, those who are brought with ambulance.

**I: With ambulance.**

R: Yes.

**I: Is it because of cost that you also refer them to Kenyatta, because they can also go to Nairobi hospital, they can go to Agha Khan?**

R: Yeah, or Kijabe. They always say they want to go to Kenyatta or Kijabe.

**I: Because of cost as well?**

R: yeah.

**I: Because most of them are poor; they don’t have insurance…**

R: And also they don’t have knowledge of other hospitals; they only know Kenyatta and Kijabe. If you ask them they tell you Kijabe is the one which deals with bones.

**I: They prefer…But mostly it’s Kenyatta?**

R: Kenyatta.

**I: I think that is it for now, if I have any issues that I would like to know then I might call you just to clarify some things.**

R: You are welcome; most welcome.

**I: Thank you very much.**

R: Thanks a lot.
