# Supplementary material for: Patterns of facility and patient related factors to the orthopedic and trauma admissions at the Kenyatta National Hospital: A qualitative assessment
Source: PLOS Glob Public Health. 2024 Jan 25;4(1):e0002323. doi: 10.1371/journal.pgph.0002323 (PMC10810445; doi:10.1371/journal.pgph.0002323)
Supplement: S1 File — (ZIP) [file pgph.0002323.s006.zip › KII TRANSCRIPTS/NGONG SUB-COUNTY HOSPITAL KII.docx]

| **FACILITY** | **NGONG SUBCOUNTY HOSPITAL** |
| --- | --- |
| **INTERVIEWER** | **Dr Maxwell Omondi** |
| **TRANSCRIBER** | **Dora Bloch** |

**I: My name is Dr Maxwell, I’m an orthopaedic registrar and also investigator of this study that we have to seek to understand the factors that are associated with referral of patients from Ngong to Kenyatta. Because Ngong is one of the facilities we have noted that PIDs orthopaedic admissions to Kenyatta referral hospital. This will be a thirty minutes discussion. So your name is?**

R: I’m Julius Nyakundi.

**I: Julius?**

R: Nyakundi.

**I: Nyakundi?**

R: Yes.

**I: Aha…**

R: Clinical medical officer.

**I; Orthopaedic?**

R: Yeah, medical officer

**I: You have been here for how many**

R: Five years.

**I: Five years now?**

R: Yeah, but I completed my orthopaedics in 2018.

**I; In 2018?**

R: Yeah.

**I: so initially you were here as a general?’**

R: Yeah, I was here as a general and then I went for a diploma orthopaedics in KMTC Nairobi.

**I: So, I wanted to understand, where do you refer the orthopaedic cases or what are the places, which are the facilities do you refer them to mostly?**

R: Mostly?

**I: Mmhh.**

R: Mostly this being Kajiado County, you are supposed to refer to Kajiado. But because of distance, most patients decline to be referred there. They prefer to go to the nearest because of proximity; either Kenyatta, Mbagathi and other private hospital which offer orthopaedics…

**I: Which ones?**

R: There is one; there is ST. Peters.

**I: Oh, St Peters in Uthiru?**

R: Yes, in Uthiru. That’s the most; St. Peters Kikuyu…

**I: In Kikuyu also?**

R: Yeah.

**I: Mostly where do you do?**

R: St. Peters

**I: Mostly they go to St Peters?**

R: The private?

**I: No, the general; overall.**

R: No, most of them land in Kenyatta.

**I: Most of them in Kenyatta?**

R: Yeah, most of them land in Kenyatta.

**I: But those who can afford to pay…**

R: But those who can afford to pay, they go to St Peters…No St Peters take NHIF; those who have NHIF, most of them go there by there.

**I: For…**

R: For surgeries.

**I; For cash….**

R: Because they don’t pay any extra amount.

**I: Apart from NHIF?**

R: Yeah, apart from NHIF.

**I: Oh, they don’t pay an extra thing…**

R: The other thing for the bipolar thing that is the only time they can top up.

**I: But for the other things?**

R: The other things, everything is done by NHIF.

**I: Do you say KNH is the major place that they go to or Uthiru; overall?**

R: I can say, overall they go St Peters.

**I: Majority?**

R: Majority, and Kenyatta, those two.

**I: St Peters and Kenyatta that’s where majority go to?**

R: Yeah, they share by the way.

**I: They share?**

R: They share.

**I: So, then Mbagathi will be the slowest?**

R: Yes, the slowest; that’s the…

**I: Do they ask that “I want to go to Kenyatta” or it is you who tells them?**

R: No, they don’t ask; normally when it comes to referral we ask them whether they can be referred to Kajiado. Most of them decline, they say they want to go either to Mbagathi, Kenyatta or they ask “Which hospital do you think is near here?”, now that we stay around here because Kajiado is very far.

**I: Very far.**

R: So you will give the options; you can go to Kenyatta, there’s another one called St Peters or Mbagathi or Kikuyu. Then they come up with a solution and we send them where they…

**I: So, you give them the options and then they choose?**

R: Yeah they choose.

**I: Most of them choose Kenyatta….**

R: Yes, most of them choose Kenyatta, Mbagathi and St Peters.

**I: And St. Peters?**

R: Yes.

**I; So, the patterns of referral, what would you say there are types of fractures or types of orthopaedic do you refer mostly?**

R: Mostly we refer those complex ones like [inaudible 04:29] hip, and those fractures like pelvic fractures that need a most specialized attention and those that need [SIAM?04:56]

**I: Those that need SIAM.**

R: SIAM, yes.

**I: Like which ones?**

R: The SIAM?

**I: Yes.**

R: DFN, DHS things that mostly need the…and some of the k-wires.

**I: K?**

R: K-wires, some of the complicated that needs…

**I; What of the long bones like femur fractures…**

R: Long bones like femur fractures yes.

**I: You refer?**

R: Yeah, we refer them. Those ones that we see…We refer them. You know here we don’t have…

**I: Even tibia.**

R: We have a general surgeon here, but because most of the time you find the general surgeon is not conversant with these things mostly, he normally does those that he can like plating of the [inaudible 05:59]

**I: That one you do here?**

R: Yes, those ones we do here.

**I; And you have the implants?**

R: The implants no, we source from outside. Normally the implants come from…there is somebody who comes with them. He sells to the patients and when the patient pays, that is the time you can fix it.

**I: But plating…had to check.**

R: Plating, not all of them; not all of them I [laughing]

**I: You can manage.**

R: Yeah.

**I: You are right.**

R: And that is the joint, the ones near the joint those ones need surgery.

**I: But the others ones….**

R: The other ones you just do it.

**I: [inaudible 06:39]**

R: Even [inaudible 06:40] can give you headache.

I: Yeah, they are a problem.

R: Those cross you fix them,

**I: You can sit there the whole night**

R: Yeah the whole night. In fact femur and tibia huh

**I: Those ones you refer?**

R: Yes, most of them we refer; a few we do.

**I: Are you aware that KNH gave these referral guidelines from 1^st^ of July; these referrals from 1^st^ of July so you don’t refer all patients.**

R: To Kenyatta.

**I; And if you refer, there is a system. Are you aware?**

R: Yeah, I am aware.

**I: How does it work?**

R: Normally what we do…You mean to KNH, because to private you can just walk in. normally we mobilize the patient when he has a fracture; we mobilize the patient first, you explain to the patient first. From there you refer after doing your thorough investigation.

I: You refer?

R: Yeah.

**I: So, do you just give a letter or you talk to KNH first?**

R: We talk to KNH first; when you go to KNH, you must talk first.

**I; First?**

R: First. Even Mbagathi, you have to talk to them.

**I: So you call KNH, aha**

R: You call KNH…

**I: First. For approval?**

R: For approval, whether they can receive the patient or not.

**I: So sometimes they refuse?**

R: Yes, they refuse; they can refuse. They tell us “You have an orthopaedic surgeon in Kajiado referral, why are you referring here?”

**I: Ah**

R: that distance…

**I: Becomes an issue?**

R: It becomes an issue for the patient.

**I; So…**

R: At the end of the day…

**I; They take?**

R: Sometimes you tell the patient to go and talk to them.

**I; to who?**

R: Not all patients are accompanied by nurse from here, some are not an emergency most of them; so most of them they go by themselves.

**I: So if…**

R: When they go by themselves by the way, they can even be helped more than when you escort them. So that’s the problem.

**I: So sometimes KNH decline?**

R: Yeah they decline, not sometimes, they decline. You are told take your patient to Kajiado, you have an orthopaedic surgeon.

**I: So they just need to approve?**

R: Yeah, just to approve.

**I: Sometimes they approve?**

R: Yeah.

**I: When do they approve, when do they decline?**

R: When you call them and tell them the patient is bleeding in the pelvic fractures,

**I: They take.**

R: Those ones they take. They have to evaluate first before they tell you yes.

**I: Femur…**

R: Femur they tell you that’s a small thing.

**I: Take it to…**

R: Take it to the nearest…Unless it is involved the vascular injuries and the nerves. Unless those things are involved, that is when they can take.

**I; If there is pelvic and fracture plus vascular injuries…**

R: Yes, vascular injury, those ones we refer; they accept

**I; That one they take?**

R: Yeah, those ones they take.

**I; But any other thing, they will…**

R: They will tell you no.

**I: Go elsewhere.**

R: Yeah, unless it’s…or neuro.

**I: You also refer patients with politrauma cases?**

R: Politrauma cases, yeah.

**I: Politrauma.**

R: Yeah, politrauma cases with neuro. Those ones we refer.

**I: Neuro…**

R: Because they we don’t have a…

**I: With head injuries.**

R: With head injuries in short. We don’t have a neuro here

**I: Because you don’t have a neuro…**

R: They will accept

**I: That one they will accept?**

R: Very fast.

**I; They will not tell you to go to county?**

R: They will not tell you to go to county.

**I; Since the guideline came into effect on 1^st^ of July 2021, has there been a change in the type of patients you refer to KNH?**

R: Yes.

**I: What changes are there, before…**

R: Before you would refer any patient.

**I: Before you would refer any patient?**

R: Yeah.

**I: Any patient is what, any…**

R: Any kind of injury or emergency. But nowadays because they know we have surgeons; we have orthopaedic surgeon, we have a general surgeon…

**I: Do you have an orthopaedic surgeon here?**

R: No, we have at Kajiado.

**I: At Kajiado?**

R: Yeah.

**I: But I’m talking of 1^st^ of July.**

R: 1^st^ of July.

**I; Last year.**

R: Last year.

**I: That is when they enforced this referral.**

R: Yes.

**I: So, has there been a change in terms of patterns of patients you refer?**

R: Yeah, there is a change; numbers went down.

**I: Pardon?**

R: For referrals, we don’t refer…Patterns as what, what do you mean?

**I: As in, has the number of patients you refer to Kenyatta reduced, has…**

R: Yeah, by a big margin.

**I: Referrals have reduced?**

R: We rarely refer to KNH; we rarely refer. Unless it is something that the surgeon cannot handle.

**I: Why has it reduced drastically to KNH?**

R: Because of guidelines restrictions.

**I; Due to the restrictions?**

R: Yeah, due to the restrictions of Kenyatta; which is a good thing I can say.

**I: Because of the restrictions, they have reduced?**

R: They have reduced.

**I: Mostly now what do you refer now because of those restrictions?**

R: Pelvic, because they are serious ones.

**I: Like the pelvic fracture.**

R; The pelvic fracture, with bleeding that is, just the fracture. Complex fractures.

**I; Complex fractures?**

R: Yeah complex fracture with politrauma; with neuro.

**I; With neuro.**

R: Yes, those ones we shall…Or any politrauma with abnormal injury; maybe the patient is bleeding like that. That one can be referred even if it’s a simple fracture. That one you can refer and then the fracture can be fixed.

**I: For politrauma patients?**

R: For politrauma patients.

**I: Those are the only ones you refer?**

R: Yeah.

**I: The long bones you don’t refer there?**

R: The long bones we don’t refer there. That one no, they cannot accept, Kenyatta? In Mbagathi they can accept but Kenyatta they cannot.

**I: KNH cannot.**

R: Mmhh. Yesterday there is one I referred from here who had a fracture femur, we referred to; the guardian told us to send them to Mbagathi. They went to Mbagathi, unfortunately the patient didn’t have NHIF. They told them that they don’t see patients who don’t have NHIF there.

**I: In Mbagathi.**

R: Unless you have cash. That [inaudible 13:31] was needy. So, they were denied.

**I: And the patient went where?**

R: They went to, I think they went to Eastleigh.

**I: Oh?**

R: There is a… Eastleigh

**I: Eastleigh?**

R: Yeah.

**I: But Eastleigh you have to pay.**

R: I don’t know how he landed there.

**I: He was sorted?**

R: But he was sorted. He was told that water levels was a bit down.

**I: Huh?**

R: You know Somalis? [Laughing]

**I: Yes.**

R: Somalis they can take anything. So, I don’t understand. I was told today, but I didn’t understand…

**I: How they…**

R: How they….

**I: So, there is no guideline you were given by KNH to say that this is the guideline.**

R: They just become restrictive but there is no…

**I: They are very strict. No guideline that…**

R: When it comes to referrals, they are very strict

**I: Just restrictions?**

R: Yes.

**I: Verbal?**

R: Verbal.

**I: No…**

R: I think they sent a MEMO.

**I: They sent a MEMO?**

R: They sent a MEMO yeah.

**I: That?**

R: No referrals to KNH unless the surgeons cannot manage those cases.

**I: They collected…**

R: Sometimes they say those surgeons are the ones who are supposed to refer.

**I: Surgeons?**

R: Yeah. Surgeons and MOs, they are the ones who refer.

**I: Do you have a copy of that circular?**

R: No, I don’t think so. I saw it long time ago, I never saw it again.

**I; But they actually sent a circular.**

R: Yeah, they sent a circular.

**I: Effective that date?**

R: Yeah.

**I: Saying any more referrals, consultants and MOs must directly talk to KNH and…**

R: On phone.

**I: On phone.**

R: Yeah.

**I: Before cases go there?**

R: Yeah. Confirm whether there is a bed for that patient or if it’s a case that cannot be handled by them. It is that serious.

**I; Oh?**

R: It is that serious.

**I: That one has changed, yeah?**

R: Yeah, that one has changed.

**I: What kind of patients do you normally refer to KNH? The profile means, profile characteristics in terms of Age…**

R: Very serious.

**I: Old people, young people, women, men. Mostly which kind of patients do you refer?**

R: Peads; paediatric.

**I: Mostly children?**

R: Yes, children that is very common; paediatric. They are the ones mostly; they are the ones that we refer most.

**I: Men, women?**

R: And the women.

**I: Huh?**

R: Men.

**I: You refer?**

R: No, even female. You know we have a theatre here. Maternity cases, most of them also they are being scanned there.

**I: No, but we are talking about the orthopaedic.**

R: The orthopaedic.

**I: Yes, orthopaedic only.**

R: Orthopaedic.

**I: Yes.**

R: Mostly are men.

**I: Mostly it’s…**

R: They are male, men are the ones who get…

**I: And male?**

R: Yes. Children and male.

**I: Children and as well?**

R: Male.

**I: Male?**

R: You said what?

**I: Male.**

R: Male, yeah.

**I: So usually old people and male?**

R: Male are the most.

**I: Not old people?**

R: Not old people.

**I: Mostly they are male?**

R: Yes, male.

**I: That one remains even after the guideline it’s still the same?**

R: Yes, still the same in fact, again men that age between 20; the motorcycle riders mostly.

**I: Between 20 and?**

R: 20 and 40 there…

[Interruption]

**I: So, these motorcycle guys…**

R: The motorcycle guys, that cluster of that age; they are the ones who most of the time get accidents.

**I: Oh, they are the ones getting accidents?**

R: Yes, they are the ones who usually get…They are our clients.

**I: The motorcycle guys?**

R: The motorcycle guys. Most of them have no NHIF.

**I: Huh?**

R: Yes.

**I: And…**

R: Most of them have no NHIF.

**I; Have no NHIF?**

R: Yes they don’t have NHIF.

**I: They have nothing?**

R: They have nothing.

**I: They all come from around within?**

R: They come from within yeah, most of them. But we treat patients; they come from as far as Rongai, Kiserian and Matasia

**I: So…**

R: They come here; Kiserian and Matasia.

**I: This a catchment?**

R: Yes, catchment area.

**I: These motorcycle people?**

R: Yes motorcycle people and Ngong itself.

**I: But Ngong has also got a facility?**

R: Ngong, which facility?

**I: They don’t have Ngong hospital?**

R: This is the whole hospital.

**I: No, Rongai, is there a hospital in…**

R: Rongai yeah, but most of them they land here.

**I: Rongai is also a big facility like this?**

R: It’s a big, it’s as big as this but this is bigger than Rongai.

**I: So, the trauma cases; what are the common orthopaedic trauma cases…I think that one you have talked about; the pelvic ones…**

R: In fact they refer patients here because I’m here.

**I; Because of you?**

R: Yeah.

**I: They don’t have orthopaedic…**

R: Yeah, they don’t have orthopaedic right now.

**I: so, pelvic fractures and also the long bones?**

R: Yes, the long bones

**I: that require II**

R: That require II yes.

**I: For Peads?**

R: Peads, yeah they refer, mostly…

**I: Do you prefer Paediatrics?**

R: Yeah, sometimes we refer this [inaudible 19:50]

**I: [Inaudible 19:51]?**

R: Yes.

**I: But are they common?**

R: They are very common.

**I; You refer them…**

R: Are very common.

**I: You refer them where?**

R: To their hospital of choice.

**I; Mostly where do they go?**

R: They go to…Most of them they go to St Peters. [Interruption] Or Mbagathi.

**I: They go to St Peters…**

R: St Peters and Mbagathi.

**I: Peters Uthiru, they also be here…**

R: Yeah.

**I: You also refer to KNH?**

R: Yes, but it’s too much work for those ones because we just refer the ones we can’t manage.

**I: So, you refer mostly to…Not KNH, you don’t refer mostly to KNH?**

R: Mostly Uthiru…

**I: And Mbagathi?**

R: Yes, and Mbagathi.

**I: Mostly.**

R: Mmhh.

**I: KNH, rarely…**

R: You refer the peads?

**I: The peads. That’s important. We are almost done. What are the reasons now for referrals? I think you have mentioned some of them, maybe we can just go through them.**

R: Distance.

**I: Distance?**

R: Yeah.

**I: instead of going to Kajiado…**

R: They prefer near.

**I: County. But why would they prefer…Those who come to KNH, what is the reasons why they want to come to KNH?**

R: The ones who…No, those ones who go to KNH…We refer to KNH very serious patients.

**I; Complicated cases.**

R: Complicated cases.

**I: Only?**

R: Yes, the rest go to these other hospitals.

**I: Why do you refer complicated cases to KNH; what is the reason?**

R: The reason why, we have got personnel in KNH…

**I: Better personnel….**

R: And equipment.

**I: Better equipment, equipment like which ones?**

R: The II you were saying

**I: Like II.**

R: Yeah, and then he personnel there, they’ve got specialists.

**I: Specialists.**

R: Yeah.

**I: What else, what are the other things that make you refer to them? So specialists here include the neurosurgeons you talked about?**

R: Yes. The major thing is the personnel.

**I: It’s the personnel?**

R: Yeah.

**I: There is nothing to do with cost?**

R: Costs, not really. Kenyatta are also high, they are not that as cheap as you think.

**I: Okay.**

R: Yeah, and that is why we only send complex things. Others go there and they change, they change to…When they go to Kenyatta others tend to go to other hospitals.

**I; But the issue about infrastructure…**

R: Infrastructure, that’s another thing

**I: That’s why you refer?**

R: Yeah.

**I; which infrastructure limitation do you have?**

R: Sorry?

**I; Which infrastructure limitations do you have that makes you refer to…**

R: To KNH?

**I: Mmhh.**

R: You know KNH, one thing they have…Their labs, their theatres; they have very many theatres there.

**I: you guys don’t have theatres here?**

R: We have one theatre, just one and we are sharing with the obsgyn

**I: The obsgyn**

R: When we have an emergency from obsgyn, we stop these orthopaedic things. Normally we refer patients to KNH or other facilities.

**I; So, no theatre space basically?**

R: Yes, theatre is one which is being shared by all.

**I: Shared by obsgyn**

R: And surgical team

**I: And surgical…Surgery team.**

R: Yes.

**I: So, that’s a limitation.**

R: But the thing we have I have said is personnel; staff are very few…

**I: Few staff?**

R: Yeah

**I: Staff for?**

R: Even we don’t have enough space here.

**I: Bed**

R: Bed; bed capacity is small. Our bed capacity is very small; it hasn’t even reached 100.

**I; It hasn’t reached 100?**

R: It hasn’t reached 100.

**I: That is for everybody?**

R: Everybody, yes; not 100.

**I: Huh?**

R: Yeah, less than 100.

**I: Less than 100.**

R: Yeah,

**I: And that’s for all? Are there beds that have been allocated to orthopaedic…**

R: No, it’s just a general thing; surgical, everyone is there.

**I: Everybody is there?**

R: Yes. And we don’t have a surgical… male, all of them are there; surgical, medical, all of them are there.

**I: Female the same?**

R: Yes, they are put together.

**I: The same thing?**

R; The same thing; we don’t have where this is surgical, this is medical. They are all together; if it’s male, it’s male.

**I: Oh, you have two wards?**

R: Yeah, male and female; that is it.

**I: Oh, so then they are all combined?**

R: Yeah, they are all combined.

**I: The caesarean section and whatever.**

R: Yeah.

**I: So, if an Obsgyn comes around, he has to know his patients; he picks and goes?**

R: Yes.

**I: General comes and looks at his patient, he picks and goes?**

R: Yeah.

**I: So less bed capacity, that one also…**

R: That one can …

**I; No orthopaedic surgeons?**

R: No orthopaedic surgeons. That’s the major thing; not everything that the general can do.

**I: He’s not comfortable doing everything because he’s not ready.**

R: He is not ready. We just help each other.

**I: You help each other.**

R: We help each other.

**I: So the drills you have?**

R: No, everything comes with the implant person. He comes with everything; it is usually a package. When he comes, he has to come with everything; he comes with the plants, he comes with the drills

**I: The patient pays for that?**

R: Yes, the patient pays.

**I: And the sterilization?**

R: They pay prior

**I; And the sterilization?**

R: Sterilization is done, here

**I: So they bring them here…**

R: It can be done there

**I; But they should…**

R: We like it being done here…

**I: Before they go.**

R: Because we don’t know how it will be done.

**I: What recommendations would you want to say regarding these referrals?**

R: For referrals, we need orthopaedic surgeons

**I: Okay, you need…**

R: Orthopaedic surgeon here

**I: Is it orthopaedic surgeon…**

R: We need another theatre

**I: You need orthopaedic surgeon, need…**

R: Another theatre, should be equipped. We have two theatres, one is not equipped, and the other is equipped.

**I; Another equipped?**

R: Yeah.

**I: Theatre.**

R: We need more; the medical personnel should be increased.

**I: Capacity increased.**

R: What else, personnel.

**I: Which personnel are you talking about?**

R: These Employees; we need more nurses, more clinicians. More nurses when it comes to care. We need more in fact right now we have a shortage that is why we refer most patients to Kajiado. You find some go to Mbagathi or…

**I: Oh, staff?**

R: Yeah. Another thing about theatre, it should be equipped with those IIs

**I: So, you need equipment, you talked about theatre equipment like II.**

R: Yeah IIs.

**I; Do you do X-ray?**

R: Yeah, X-ray we do.

**I: Oh, maybe II imaging.**

R: Yeah. We also need the small imaging like CT-scan, MRI, of which we don’t have right now.

**I: You need CT scan and MRI.**

R: MRI.

**I; But now is that another reason that you refer; the issue of CT-scans and MRI?**

R: Yeah, sometimes we refer them because of that.

**I: But if you do it, you don’t have a surgeon to fix it; if you do MRI and there is a problem, how will you fix?**

R: Like now a patient has come here; has gotten a head injury, this patient. We don’t have an MRI or a CT-scan…

**I: You refer.**

R: You refer there because there is a CT-Scan at KNH

**I; So, the reason why you also refer to KNH is also because there is a lack of equipment,**

R: Yeah, equipment.

**I: Investigations.**

R: Yeah.

**I: CT-scan…**

R: CT-Scan and MRI.

**I: These ones…**

R: Of which we have them at Kajiado; like MRI came the other day, but because of distance also…

**I: If a patient needs CT-Scan MRI, do you refer them to St Peters or do you refer them…**

R: No, St Peters don’t have MRI, don’t have CT-Scan.

**I; So this one you refer to KNH?**

R: KNH straight away where they can be done everything under one roof.

**I: That one those guys will accept? The guys for KNH will accept?**

R: Yes, they can accept after really evaluating that this patient needs to be here.

**I: This is a big reason as well.**

R: Yeah, that’s a reason; MRI we don’t have it here.

**I: Because if you don’t have this and the patient needs it, definitely he has to go to KNH.**

R: Yeah, he has to go to KNH

**I: They don’t have any other place.**

R: And you can see it’s a neural thing.

**I; Yeah that one that needs CT- Scan is a neural thing.**

R: Yeah, it’s a neural thing.

**I: Either way…So, you need orthopaedic surgeon, you need more personnel, but you also talked about you need neurosurgeon.**

R: Neurosurgeon yeah. In fact we need more specialists.

**I; More specialists?**

R: Yeah.

**I: Because without these specialists…More specialists.**

R: Yes, and that is why we refer them; more patients, because we don’t have these specials here.

**I: There are no specialist here. So more neurosurgeons; more specialist.**

R: Yes, and other specialists.

**I: Yes, because if you don’t have them, then there is no…**

R: Speciality.

**I: Even if you have the CT-Scan and you have no neurosurgeon…**

R: There is no…You are doing nothing. You must have those people; those things.

**I: So, Julius it was great. Your phone number? I think they gave me another Julius who is a records person**

R: Yes

**I: I think this is good, I think I’ve now known the issue is the capacity of the facility.**

R: Capacity of the facility.

**I: You have no equipment, no orthopaedic surgeon in that case, for complex cases you refer.**

R: Yeah.

**I; But do patients have a preference? Like they say “Me I want KNH”?**

R: Yes, most of them.

**I: They have…**

R: Most of them they have preferences. Others want us to assist them…

**I: To tell them where to go**

R: Where to go; where they can be helped.

**I: These patients who have a preference for KNH, what are the reasons they are giving for preferring KNH?>**

R: When it comes to KNH, a patient cannot decide to go to KNH by herself/himself.

**I: Since that 1^st^ of July; since that circular came?**

R: Yes.

**I: But before they could decide?**

R: before, they could decide but now they cannot.

**I: So, before, those who were deciding why were they deciding to go to KNH?**

R: Before?

**I: Mmhh.**

R: Because of the services; they are special…All specialists are there. They could be…All services they needed, they could get there. That’s the reason as to why patients wanted to go to Kenyatta, then he will be referred to Kenyatta; let’s go where we can get all doctors there.

**I: All patients [background noise]**

R: Mmhh, so everything is done there

**I; Not cost. Cost is totally not an issue?**

R: Cost is not an issue

**I: Because it’s cheaper in KNH?**

R: It’s cheaper in KNH compared to private. That’s a major thing; that’s a very important point, the cost.

**I: Somebody say I want to go to Kenyatta because of cost.**

R: Yeah.

**I; others will still go to Nairobi hospital.**

R: Cost is also a reason.

**I; Cost is a…**

R: Yes.

**I; KNH is perceived to be cheaper.**

R: Yes.

**I: But guys still prefer Uthiru**

R: Yes, others come and maybe know their relatives were being treated there. There was a relative who was treated there; was treated well, now other come here and tell me “ Send us to St Peters Uthiru, my brother went there and was sorted out very nicely”. By the way St Peters workload is high.

**I: It is high?**

R: It is high, in fact it was one of hospitals which were highly paid by NHIF; it was in the magazine, I think we had that.

**I: So they are doing well.**

R: It’s not a referral hospital but does wonders.

**I: Do you know anybody there at St Peters?**

R: Yeah, I know.

**I: Who do you know? I need to talk to them**

R: Go to Wasena; Dr Wasena is the owner of that hospital.

**I: The owner? Dr?**

R: Wasena.

**I: Wasena.**

R: Yes.

**I: It’s a small place but they are doing wonders?**

R: Yes, they are doing wonders. That guy, he has helped sick people.

**I: He is a medical doctor?**

R: He is a medical doctor, I was told he did surgery, I think he went and specialised in orthopaedic.

**I: He is called doctor?**

R: Dr. Wasena.

**I: Wasena.**

R: Jerry.

**I: Jerry?**

R: Jerry Wasena.

**I: It’s a lady?**

R: No, it’s a man. Jerry.

**I: Oh, Jerry.**

R: Jerry, this one.

**I: His number, so he is called Dr Jerry.**

R: Wasena.

**I: So he is the owner?**

R: Yes, he is the owner.

**I; I can talk to him directly, or he usually…**

R: Pardon?

**I; I can talk to him directly?**

R: Yes, there is no problem

**I: There is no problem?**

R: Yes.

**I: Do you know where the facility is?**

R: Yeah, just go to Uthiru near Uthiru stage, Uthiru girls; do you know where it is?

**I: It’s on the road, right?**

R: In the road, you will just see it there; behind Uthiru girls. Just ask where St Peters is, it is very much known. It is just a walking distance.

**I: It’s a walking distance?**

R: Yes.

**I: I will call him just now**
