# Supplementary material for: Patterns of facility and patient related factors to the orthopedic and trauma admissions at the Kenyatta National Hospital: A qualitative assessment
Source: PLOS Glob Public Health. 2024 Jan 25;4(1):e0002323. doi: 10.1371/journal.pgph.0002323 (PMC10810445; doi:10.1371/journal.pgph.0002323)
Supplement: S1 File — (ZIP) [file pgph.0002323.s006.zip › KII TRANSCRIPTS/ST FRANCIS COMMUNITY HOSPITAL KII.docx]

| **FACILITY** | **ST FRANCIS COMMUNITY HOSPITAL** |
| --- | --- |
| **INTERVIEWER** | **Dr Maxwell Omondi** |
| **TRANSCRIBER** | **Dora Bloch** |

**I: As I’ve mentioned, my name is Dr Maxwell Omondi, I’m a registrar at Kenyatta doing orthopaedics, my third year. So, this is part of the data collection of my project. My study is about orthopaedics referrals from the surrounding facilities to Kenyatta and St Francis is one of the facilities from the records, are the major referral facilities for orthopaedic cases. That is what my study is focusing on; I didn’t look at other referrals but only orthopaedic from last year. So we will go through a few of those questions that are there to basically understand the issue around orthopaedic referrals. So if you don’t mind, your name?**

R: Hellen.

**I; Hellen?**

R: Mmhh.

**I: So, the emergency…**

R: Emergency clinician.

**I: Clinician. Maybe we can just go through this together. I will not write everything that is why this one will help me. Where do you refer most of your orthopaedic cases?**

R: I think most we handle, there is one at the hospital.

R2: Consultant.

**I: Consultants.**

R: But maybe the ones that we don’t refer…We don’t handle, because they could not afford or such a thing. So, according to the protocol, first we ask other level 5s; the Mama Lucy and maybe Mbagathi before we go to Kenyatta, we can’t refer direct. We first refer to those hospitals…

**I: There’s a…**

R: If we call, then there are times they would accept to receive the patient.

**I: But mostly where do you refer to?**

R: Kenyatta of course.

**I: That’s where you refer to most of the time?**

R: Yes.

**I; And then Mama Lucy as well?**

R: yes.

**I: Mostly is Kenyatta followed by Mama Lucy?**

R: Yes.

**I: Mama Lucy. What kind of cases do you refer to these facilities?**

R: Open fractures mostly. The ones that need surgery; most of them are open structures.

**I: Open fractures?**

R: Yeah, most ones we do immobilization here if there is a plaster involved.

**I: You refer open fractures, which fractures of the part of the body?**

R: Pardon?

**I: Which fractures; the long bones…**

R: Any open; the long bone majorly. Even closed, the long bones that require surgery, we do refer if they are not able to afford.

**I; closed fractures?**

R: Mmhh.

**I: Fractures if not able to afford?**

R: Mmhh

**I; that’s the key word?**

R: Mmhh.

**I: So if they can afford, we don’t have to refer? Because you have in-house orthopaedic consultants. So basically you refer for those who are not able to pay?**

R: Mmhh.

**I: So there is no really a particular pattern, anyone who is able to afford…There is no surgery you can’t do here?**

R: No, even the open ones we can do if they are able to afford.

**I: If they can be able to afford?**

R: Yeah. Our orthopaedic consultants…

**I: How many orthopaedic consultants do you have?**

R: Now 3.

**I: 3 orthopaedic consultants?**

R: Yeah.

**I: They do all, even pelvic, spine?**

R: No, not spine; spine they don’t is that right? Do they do spine. Gakuya.

R2: Gakuya does spine.

**I: Gakuya does spine?**

R: Yes.

I: So they do all orthopaedic cases; pelvic, they do?

R: Mmhh.

**I: Yeah?**

R: Yeah.

R2: But they are not residential.

R: Pardon?

R2: I am saying the doctors are not residential

R: They are visiting consultants.

**I: Three visiting consultants. Okay, they are not residential.**

R: Mmhh.

**I; But they can do all these orthopaedic, so you don’t refer because of expertise but you refer because of cost?**

R: Yeah. Majorly.

**I: Majorly is cost?**

R: Yes.

**I: I think that is clear. So, KNH came up with a guideline on the 1^st^ of July. Are you aware of that guideline; where they say you don’t refer patients to them directly because you are a referral facility? Are you aware about that?**

R: Yes.

**I: I wanted to understand, do you have a copy of that memo?**

R: last year…

**I: Are you aware about that or you heard it verbally?**

R: We heard verbally…

**I: You heard verbally…**

R: And even if you call them, they will tell you that you first refer to the others.

**I; You refer where?**

R: Mama Lucy.

**I: First refer to Mama Lucy.**

R: Mmhh.

**I: So you don’t refer directly to them?**

R: Maybe not orthopaedic.

**I: No, we are talking about orthopaedic.**

R: Orthopaedic?

**I: Yes. So, do you refer to them directly or now you don’t refer to them directly?**

R: If you call and they are comfortable receiving the patient, then we do direct the referral. But if you call and they tell you to first refer to Mama Lucy, then you refer first to Mama Lucy.

**I; Is it you who calls or anyone…**

R: Yeah; the one who is handling the patient. If I am not there and there is another clinician handling the patient, then…

**I: You call KNH?**

R: Mmhh.

**I: So, clinician at St Francis. If KNH are comfortable…**

R: If they consent, we refer the patient.

**I: You refer?**

R: Mmhh.

**I: Otherwise you take them to Mama Lucy?**

R: Mmhh.

**I: Are there times you refer outside elsewhere; do you refer to Kijabe as well?**

R: There are times we refer… [Interruption] so, it depends with the patient. There are times we refer to Kijabe, times we refer to Kikuyu

**I: Pardon?**

R: Such times we don’t have a word of “I want to refer to such a hospital”, it’s the patient who…

**I: For how long have you been here?**

R: At the emergency?

**I: Yeah. Last year you were here?**

R: Yeah.

**I; Have there been a change from how you make referrals since that 1^st^ of July, or this issue is…**

R: To KNH?

**I: Before and…**

R: No, we have to make a call first.

**I: Even before?**

R: Yes, you must make a call.

**I: So nothing has changed?**

R: Yes.

**I: The only that has changed is that this issue that you refer to Mama Lucy?**

R: If they don’t consent to receive the patient.

**I: That was there before, or that’s a new thing?**

R: Referral to Mama Lucy?

**I: Yes.**

R: No, it’s not new.

**I; Pardon?**

R: It’s now new. There are people you call and they tell you, you refer to Mama Lucy first. If they can’t handle, then they will refer to Kenyatta.

**I: Then there is this verbal communication you got about referrals, what was it; this verbal communication you got.**

R: That there is no direct referral to Kenyatta from here; you first refer to Mama Lucy.

**I: Which has been there; I am trying to see whether there has been any change.**

R: Oh, I don’t know; I can’t really say. I’ve not been keen on that. All I know is you have to call Kenyatta and they consent or they don’t consent.

**I: The profile of patients [interruption]. So the profile of patients, what can you say are the characteristics of patients you send; old age, poor, what are the types, the nature of patients.**

R: Here we receive all types.

**I: Which ones do you refer? On referral; the ones that you refer.**

R: Any one because they request

**I: That’s correct, but is there any profile you noted in them that you tend to refer thi type of patients?**

R: The elderly.

**I: The elderly?**

R: Mainly the elderly because…We can say mostly on request.

**I: on request…So this particular people who…**

R: On request; relatives request.

**I: Oh, on relative request?**

R: Yes.

**I: So this relative request, there is no pattern you see like able to afford.**

R: I am saying they are not able to afford, so they ask to refer.

**I: There is nothing unique about this group of people who you tend to refer; there is nothing unique about them, it’s just…**

R: No, I’ve observed anything

**I: On cost ground.**

R: Yes, they would say they are not able to afford.

**I: They are not able to afford, and that basis the…**

R: Yes.

**I: You cannot say they are elderly, you can’t say they are children, you can’t say they are middle aged; cutting across. You can’t say they are male, you can’t say they are female.**

R: No. again then, the orthopaedic cases are not so many.

**I: They are not so many?**

R: No, they are not. We can go even a week without having even one.

**I: Oh.**

R: Yeah. They are not many.

**I: They are not that many.**

R: Yes.

**I: And the catchment population for you guys?**

R: Mostly for orthopaedic, it’s now the surrounding area especially Thika road because of the accidents. Just around.

**I: Around is, this is Mwiki…**

R: Kasarani,

**I: Kasarani**

R: Kasarani, Mwiki, Githurai, those sides.

**I: Githurai. What are the causes of the injury; is it a fall, RTA?**

R: Mostly RTA.

**I: Motorcycles?**

R: Motorcycles, cars accidents.

**I; Car accidents?**

R: Yes.

**I: The common orthopaedic patients you say there is nothing like common, it is basically…you can’t really define the cases that you defer but it is just cutting across?**

R: Mmhh.

**I: Cutting across, since you have visiting consultants, huh?**

R: Mmhh.

**I: Who can handle all orthopaedic cases, right?**

R: Mmhh.

**I: You wouldn’t say they are mostly male or female?**

R: Mostly…

**I: Pardon?**

R: We can’t really…

**I: On average?**

R: I think I’ve seen both.

**I; Equal, so there is no dominant sex?**

R: Mmhh.

**I: So, you have seen both male and female in equal proportions?**

R: Yes,

**I: You can’t say mostly it’s male, you can’t say mostly it’s…**

R: No.

**I: Pardon?**

R: No, not unless we check the records, because I can’t tell.

**I: You can’t tell?**

R: Mmhh.

**I: The reasons for referral you have said it’s basically financial grounds.**

R: Yes, or patient’s preference.

**I: Or, patients preference.**

R: Mmhh.

**I: To KNH, the patients’ referral to Kenyatta.**

R: There are those who prefer to go there.

**I: There are those who prefer?**

R: Yes.

**I; So, those who prefer, what are the reason they are prefer Kenyatta?**

R: I think still it should be financial; the reason they are saying their NHIF will cover them at Kenyatta.

**I; It’s still financial?**

R: Yeah.

**I: It’s not like better quality of care or anything?**

R: Okay, I would say maybe especially at night, they prefer to go to Kenyatta because of the visiting consultants, we may not be able to get them at night. We might get some, but maybe most of the time we might not be able to.

**I: So, visiting consultants are not easily available?**

R: They are…

**I: At night.**

R: But at night it might be a trouble.

**I: At night?**

R: Yes.

**I: So if it is an emergency and it has come at night, sometimes**

R: We have no option than to refer

**I: So in that case you refer to KNH?**

R: Mmhh.

**I: So, you don’t cover 24/7?**

R: They are there, but also the logistics of the hospital.

**I: Pardon?**

R: There are some logistics of the hospital so sometimes you will need to refer.

**I: Which logistics?**

R: There is the deposit and all.

**I; Oh.**

R: The financial part.

**I; It just comes that financially.**

R: Yes.

**I: And the issue about implant availability and stuff like that?**

R: They are available.

**I: They are all available?**

R: Mmhh.

**I: There are no constraints about implants?**

R: Mmhh.

**I: Equipment; X-rays, CT- scans.**

R: We have all.

**I: Pardon?**

R: They are there.

**I: You have all those? Those are not reasons; like lack of CT-scan. not working, MRI?**

R: We don’t have MRI but we have a CT-scan and they are working.

**I: And they are working?**

R: Mmhh.

**I: But MRI you don’t have?**

R: No.

**I: Are there times you refer because of that?**

R: For MRI yes, especially if we suspect any form of…any spinal injury, then we have to refer.

**I: That means you refer for the MRI and then come back or they go…**

R: Now, they decide; the patient will decide.

**I: What is…**

R: But majorly if we see there is an issue with sensory or neuromuscular, then we refer direct for MRI and management.

**I: To KNH?**

R: Yes.

**I: So, spinal cases are the kind of cases that you tend to refer. That goes back to the initial question; the patterns of referral will be spinal cases.**

R: Which are very rare anyway, I have not seen any in the past weeks.

**I: So spinal cases for MRI since not available at the facility and refer them to KNH plus for the management. So, any recommendations you will suggest; I think we are in the last part now.**

R: On the referrals?

**I: What would you suggest regard the issues we have discussed, how would you make it better, are there any challenges so far that you are experiencing?**

R: In referral?

**I: In referral of orthopaedic cases.**

R: None.

**I: Any recommendations you have that..**

R: On our side or the other side?

**I: Your side.**

R: I think, recommendations would be we get at least a stipulated document of the referral.

**I: You need a documented?**

R: Yes, on how to go about it because most of the time it’s truly hectic. You call, you are transferred to this one, you are transferred to another person, at the end of it you don’t get to help the patient.

**I: So the patient end up in Mama Lucy?**

R: Yes.

**I: So, referral protocol for clarity?**

R: Mmhh, because somebody maybe did not want to work at night or see the patient. I don’t know what happens in KNH, because you call somebody says “We can’t allow the person” but with no reason.

**I: They don’t give a reason for that?**

R: No.

**I: Anything else? Anything else that you can recommend?**

R: I know maybe the referral to Mama Lucy is an issue we had already discussed, but I would still think direct referral is the best.

**I; To?**

R: Direct referral to KNH is the best because once you refer to Mama Lucy, there is still time lost for the patients. If you refer to Mama Lucy, then they to refer to KNH, that time frame is doing a lot of harm for the patient.

**I: Yeah, because most of the times when we refer [inaudible 19:36]**

R: If we could have referred directly

**I: If you can refer directly instead of referring, because Mama Lucy Kibaki will eventually refer them to Kenyatta.**

R: It will still waste a lot of time.

**I: Wastage of time…**

R: For the patient.

**I: For the patient. So even now if a patient comes and he is not able to afford for, or any other reason, then you have to refer to Mama Lucy; that is the procedure now?**

R: If you call Kenyatta, without consent [inaudible 20:27] if they are not comfortable, they will tell you no first go to Mama Lucy.

**I: Which is the most common?**

R: Mmhh.

**I: Unless it’s a complicated case like a spine?**

R: Mmhh. But I have not said they decline many times; there are times that we call and they are very comfortable.

**I: Even on grounds of cost?**

R: Yes.

**I; Like femur fracture?**

R: Yes, they don’t even ask if it’s the cost or whatever it is why you are referring. For as long as it’s a patient who will benefit from their end, they will pick.

**I: So they don’t really put restrictions like this one refer to Mama Lucy?**

R: No, I have not heard about them asking about the cost or anything.

**I: This issue of referring to Mama Lucy is not really common, where they tell you to refer to Mama Lucy?**

R: For orthopaedic cases?

**I; Yes.**

R: For orthopaedic cases…Maybe for the other, but for orthopaedic cases, the ones I have referred at least I have gotten a go ahead to.

**I: They always say it’s fine bring them?**

R: Yes.

**I: It’s only other cases that they are…**

R: Most, most of the other cases are the ones that…

**I; But orthopaedic they accept?**

R: Yeah, they are lenient by the way.

**I: They are lenient?**

R: Mmhh.

**I: They tend to accept?**

R: Mmhh.

**I: Because they know probably Mama Lucy…**

R: Mama Lucy are not able to handle. For orthopaedic I think they are much better and easier to refer.

**I: I think we’ve gone through quite a bit, so the only thing that I’m not…There’s no really a type, there is no profile; there is no characteristics of the patient you have said this is the kind of patient we tend to refer. If a patient walks in, you can’t predict and say this one we will refer. There is nothing you will see in them?**

R: Maybe just the walk-alone patients; they are the ones that come alone and maybe it’s a [inaudible 22:33] has gotten, they have no relatives, some.

**I: Those ones you tend to refer?**

R: Yes.

**I: That was question number; profile, number 3. For the profile you say walk-alone patients or those…**

R: Without relatives, y

**I: Without relatives, you tend to refer them?**

R: Mmhh.

**I: That is what you have noted?**

R: Because we can’t handle them

**I: Pardon?**

R: Because we can’t handle them.

**I: Because you can’t?**

R: Because, now their capability; if they are not able to cater for their expense…

**I: They just have to go.**

R: Mmhh.

**I: I think we have gone through all these… What of paediatrics, do you have a lot of paediatrics?**

R: Very few.

**I: So, you have got very few paediatrics?**

R: Referral.

**I: Even cases, do you have…**

R: Cases we do, but we don’t refer them.

**I: Paediatric referral is minimal.**

R: Mmhh.

**I: Very few paediatric referrals since it is easy. You are saying it is easy?**

R: Mmhh. Their cases are majorly maybe plaster.

**I: Since it is conservative management.**

R: Mmhh.

**I: Pardon?**

R: Yes; that one we are able to do.

**I: Those ones you are able to handle so you don’t need to refer them; you don’t refer…Which kind of cases do you refer for paediatrics if you can remember?**

[Interruption]

**I: Supposed someone has orthopaedic and is a politrauma case, do you handle it here as a structure…**

R: Yes we do but it’s the neurosurgeon that…

**I: Do you have a neurosurgeon here?**

R: Visiting also.

**I: Visiting?**

R: Yeah.

**I: So when it’s politrauma with a head injury, do you refer or do you manage them here?**

R: It depends also whether the neuro is available, but I can say his availability…

**I: Is not guaranteed?**

R: Mmhh.

**I: So then politrauma cases you tend to refer?**

R: Mmhh.

**I: Politrauma cases with head injury you refer to KNH.**

R: Yes.

**I: Mostly?**

R: Yes.

**I: Sister we are just discussing orthopaedic referrals and we are almost done. Just want to appreciate, because you are one of the major facilities that refers to Kenyatta. So I want to basically understand what are the issues and what can be done to improve on that. So, if you are to give recommendations, what else could you recommend; do you recommend that you guys have resident consultants?**

R: Yeah, what is giving us…

**I: Pardon?**

R: Most of these people [inaudible 26:27] but the major issue is on NHIF [inaudible 26:29]. It’s the major reason why we refer.

**I; Not even the availability of visiting consultants?**

R: If maybe you are requesting for an implant maybe 200 and above, some people may not be able to afford.

**I: Because implant is expensive, so they are not able to afford, so nothing can…**

R: No NHIF. I think very [inaudible 27:02]…

**I: So they are really, pardon?**

[Background noise]

R: [inaudible 27:08] refer to Kenyatta, it’s cheaper going to Kenyatta.

**I: Anything else, because we are coming to the end. Any other issue that you would want to talk about on this orthopaedic referrals?**

R: I think that’s all.

**I: Pardon?**

R: That’s all.

**I: That’s all for now. I appreciate taking your time. As I told you, this is part of a thesis, once I’m done, I will do a report but we might also invite a few of you; one or two, to receive my designation. So if you are available we will invite you through the let’s hook, and if you are able to get time allow you come, I know it’s a private place so that might be an issue allowing you to come…so we might just want for you to come and give feedback directly. Those are issues we can discuss later on. Any question?**

R: I don’t think if I have a question for you now.

**I: Clarification?**

R: I don’t know if the names will appear anywhere?

**I: We will just write 01, 02, 03 here.**

R: Okay.
