# Supplementary material for: Patterns of facility and patient related factors to the orthopedic and trauma admissions at the Kenyatta National Hospital: A qualitative assessment
Source: PLOS Glob Public Health. 2024 Jan 25;4(1):e0002323. doi: 10.1371/journal.pgph.0002323 (PMC10810445; doi:10.1371/journal.pgph.0002323)
Supplement: S1 File — (ZIP) [file pgph.0002323.s006.zip › KII TRANSCRIPTS/ST PETERS ORTHOPAEDIC KII.docx]

| **FACILITY** | **ST PETERS ORTHOPAEDIC** |
| --- | --- |
| **INTERVIEWER** | **Dr Maxwell Omondi** |
| **TRANSCRIBER** | **Dora Bloch** |

**I: So that they may know where we stand.**

R: NA is…

**I: patient who are not referred.**

[Phone interruption]

**I: So you refer KNH for?**

R: We mainly refer due to cost.

**I: Due to?**

R: Cost.

**I: Due to cost?**

R; Mmhh.

**I: So PIDs,**

R: PIDs we refer Kijabe.

**I: Adults KNH?**

R: KNH yeah.

**I: Which kind of cases do you refer; are there specific cases you refer?**

R: Not really, we refer…

**I: Is it long bones…**

R: I think any trauma that is not able to...I think any that is not able to meet the amount that we are requesting for, we refer to KNH.

**I: Not Mbagathi, why KNH in particular?**

R: Because some already came from Mbagathi.

**I: The patient prefer to go to KNH or…**

R: The patient prefer to go to KNH.

**I: So patients…KNH had [phone interruption 01:16] referral guidelines that was enforced in the 1^st^ of July, are you informed about them?**

R: No.

**I: They didn’t inform you about that? But when you refer, is there any resistance from KNH?**

R: Yes.

**I; There is resistance. So they advise…**

R: They tell us to take them to Mbagathi.

**I: That is I think question number 2. So, KNH advice to refer to Mbagathi. So nowadays you refer to Mbagathi?**

R: Mmhh.

**I: All cases that come you take to Mbagathi?**

R: We start with Kenyatta and when they refuse we take them to Mbagathi.

**I: But there are cases Kenyatta takes?**

R; A few.

[Phone interruption]

**I: Sometimes take to Mbagathi since the referral guidelines? [Background noise]**

R: Mmhh.

**I: So, maybe the cost not equipment?**

R: No.

**I; what of politrauma cases who need neurosurgical interventions?**

R: Probably we can do everything.

**I: Oh, even the neurosurgery you can?**

R: Yes.

**I: So you can do everything here?**

R: Yeah, we can do everything

**I: So mainly it’s not specialist, mostly it’s cost?**

R: Mostly it’s cost, but average we do [inaudible 03:00]

**I: And the patient’s preference, is that…**

R: Pelvic surgeries

[Interruption]

Q1. We mainly refer due to cost. We refer paeds to Kijabe Mission Hospital and Adults to KNH

Any trauma case that are not able to meet the cost.

Q2. When they call KNH, KNH advice to refer to Mbagathi DH – however they take most of the patients but patients end up to Mbagathi since the referral guidelines were instituted.

0725 641 410 – Gisaine -Matron

Q5. Some patients prefer to go to KNH. Reasons for referral are

- Patients who need ICU/HDU care
- Neurosurgeon/neurologist care
- Self-requests due to financial constraints
- Neurological complications
- Head injuries – especially polytrauma patients

Q6.

- KNH needs to accept urgent patients who need ICU/HDU
- Patients prefer KNH due to trust/quality is not good in Kimabu DH
