# Supplementary material for: Patterns of facility and patient related factors to the orthopedic and trauma admissions at the Kenyatta National Hospital: A qualitative assessment
Source: PLOS Glob Public Health. 2024 Jan 25;4(1):e0002323. doi: 10.1371/journal.pgph.0002323 (PMC10810445; doi:10.1371/journal.pgph.0002323)
Supplement: S1 File — (ZIP) [file pgph.0002323.s006.zip › KII TRANSCRIPTS/THIKA LEVEL5 HOSPITAL KII.docx]

| **FACILITY** | **THIKA LEVEL 5 HOSPITAL** |
| --- | --- |
| **INTERVIEWER** | **Dr Maxwell Omondi** |
| **TRANSCRIBER** | **Dora Bloch** |

**I: So, this Thika level5, right?**

R: Yes.

**I: Hospital, and we want to have a small discussion about orthopaedic referrals; orthopaedic referrals from Thika level 5 to Kenyatta referral hospital. This is part of my thesis, part of proposal; part of the research we do as orthopaedic surgeons in Kenyatta, university of Nairobi. By the way you were trained there as well?**

R: Mmhh.

**I: You trained there as well. So, we are doing it as a team, all orthopaedic; all registrars must do a research because part of it [inaudible 00:42]. It will take us about 30 minutes and it’s basically the questions you can see. So, you are Dr [inaudible 00:50], you are the MO intern and you are in charge of the orthopaedic ward?**

R: Mmhh Yes, I’m a nursing officer but…

**I: At the orthopaedic ward?**

R: Yes.

**I: So, there are about 5-6 questions there. When you refer orthopaedic cases, where do you refer them?**

R: We handle some; able to handle some orthopaedic cases, the ones that we cannot handle, we refer them to Kenyatta.

**I: You refer…**

R: To KNH

R: Kenyatta national hospital.

**I: To Kenyatta. Any other place you refer?**

R: We also do Kikuyu.

**I: Kikuyu?**

R: Yes.

R2: St Peters.

**I: St Peters?**

R2: Again now….

R: In Kijabe.

R2: In Kijabe too.

R: Kijabe hospital.

R2: Are we involving the spinal cases?

**I: Even spinal ones**

R2: For the spinal we go to the spinal centre.

**I: Spinal centre?**

R2: Yeah.

**I: Spinal injury hospital?**

R2: Yes.

**I: But mostly it’s Kenyatta?**

R2: Yes, Kenyatta the most.

**I: What kind of cases do you refer to Kenyatta?**

R: Mostly spinal as he said, then head injury and complex pelvic injuries.

**I: Complex?**

R: Pelvic injuries.

**I: Pelvic injuries. Aha.**

R: Mainly.

**I; Mainly?**

R: Yeah.

**I: Only those two?**

R: Yeah.

**I: Those are the main ones you refer?**

R: Those are the main ones.

**I: Orthopaedic, the long bones?**

R: We are able to do fracture femurs, tibia, but foot we also refer depending on extent. But simple fractures we handle them from here.

**I; So, those long bones you can refer?**

R: We can refer depending on the extent but mostly we can do here.

**I: The long bones…**

R2: Pelvic

**I: is done here?**

R: Yeah.

**I: KNH has been having a referral guideline, I think from 1^st^ of July last year…**

R: Other things we refer is perhaps injury to tendons especially for the hand…

**I: Hand…**

R: Forearm.

**I: Forearm injuries**

R: Yes, extensive we refer there.

**I: So extensive injuries are those ones you refer?**

R: Yeah.

**I: The crush injuries of the arm?**

R: Yes.

**I: Apart from the spinal and pelvic, there is this complex had injury; tendons.**

R2: Yeah.

**I: Those ones you refer?**

R: Yes.

**I: Apart from that, anything else maybe he’s not talked about? Long bones, femur, tibia those ones you handle here?**

R2: We handle here.

**I: You don’t refer those ones?**

R2: We don’t refer. But we do refer where we get there is a long bone that needs plastics.

**I: So, long bones that require?**

R: Plastics.

**I: Plastics Review.**

R2: Review.

**I; That one you refer?**

R2: We refer.

**I: Significant soft tissue injury?**

R: Yes.

**I: So, patients with degloving injuries?**

R2: Yes, degloving

**I: Injury that require plastic.**

R2: Mmhh.

**I: Plastic review, those ones you refer?**

R: Mmhh.

**I: In July last year, KNH came up with referral guidelines that restricted patients who are being referred to KNH. Are you aware that KNH restricted referrals to KNH in July last year?**

R2: I am not aware.

**I: You are not aware?**

R2: I was not here by then

**I: You were not here at that time?**

R2: Mmhh.

**I; But when you refer patients, how is that process; how do you refer them? Do you just tell patients to go?**

R2: No, we have to make a call first…

**I: You make a call?**

R2: We make a call; mostly the call centre, then…

R: [inaudible 05:29]

**I: Call centre.**

R: Yeah, then you are directed to the significant department.

**I: Directed to…**

R2: The department, then we talk to the relevant person that is there.

**I: Department, for concurrence?**

R2: Yes.

**I: So, before you refer, they must tell you they are ready?**

R2: Yes, so they will tell you “We are ready, we are not ready”, because as we refer, we have to write in our referral forms so and so at the referral accepted a certain time.

**I: Who calls normally, is it you is it the MO?**

R2: No, the MO in charge.

**I: So, you are the one who mostly call or the registrar?**

R: I can call or the registrar; another one call the registrar.

**I; So, the MO intern…**

R2: Or the registrar.

**I: Or registrar.**

R2: Mmhh.

**I: Consultant doesn’t call?**

R: They can call

R2: They can call. If he’s the one that this is available, then he can call.

**I: Call KNH?**

R: Mmhh.

**I: Nurses don’t call?**

R2: No.

**I: Even before?**

R2: They don’t.

**I: For a long time they don’t call?**

R2: They don’t.

**I: It’s only you guys who call?**

R2: We have to tell them to call, because they know more about the patients.

**I: To explain and…So you talk to their counterparts…**

R2: Yes

**I: Are there times that they refuse to accept?**

R2: Definitely

**I: Orthopaedic case?**

R2: Yes.

**I: Like which ones do they refuse?**

R: It depends with not per se the case, but maybe because they don’t have…

R2: Because of capacity.

**I: Huh?**

R2: Because maybe of the case that you are taking, maybe they don’t have that space or something.

R: Maybe they don’t have space, or they want…

R2: Or they want further workups

**I: Or they want further work up?**

R: Yes.

**I: That is orthopaedic cases?**

R: Yes.

**I: Which further workup of orthopaedic cases do they want?**

R2: Maybe you have done just an X-ray, but they want a CT scan or an MRI.

**I: So, they tend to do and MRI and then bring?**

R: Especially concerning the [inaudible 07:26] especially those ones.

**I; CT scans, [inaudible 07:35]**

R: Yes, [inaudible 07: 36]

**I: Referral for spinal, especially spinal injuries?**

R: Yes.

**I: Anything else? That is the process of that referral; how it happens?**

R: Yeah, that is what usually happens.

**I: So, this issue of guidelines you are not aware about it?**

R: I was not aware

**I: You are not aware about it?**

R: Maybe it could have come, then it’s in the office…

**I: How long have you been here?**

R2: I’ve been here since 2017.

**I: Okay. The kind of patients that you refer, what are their kind of profiles in terms of age; are they young, are they old. What are the kind of patients you normally refer?**

R: Middle aged patients because…

R2: Middle aged.

R: They have a lot of [inaudible 08:25]

R2: So middle aged.

**I; What is middle age?**

R: Middle age; in the middle, 30s there.

**I: 30s and?**

R2: Let’s say from 35 to 40.

**I: To 40?**

R2: Yes, because again we don’t have mostly the aged and the young ones; I have not seen.

**I; Aged and young ones…**

R2: Rarely.

**I: Are rare?**

R: The aged and young ones are…

**I: This age 35-40, why are they the most common?**

R: Trauma

R2: Trauma, Motorbikes

R: Results of traffic accidents

R2: Road traffic accidents

**I: RTAs.**

R2: RTAs.

**I: Are they men or women?**

R: Mostly men.

**I: That pattern is still the same?**

R2: Mmhh

R: That one has not changed.

**I: Nothing has changed?**

R2: Nothing has been changed.

**I: Since July…**

R: Even if we do a spot check

R2: Even if we do a spot check at the moment, it will still be the same. Even if you go to the wards, spot check will still be the same

**I: And they come from which areas?**

R: Umm, mostly outside Kiambu County.

R2: Kiambu county and Muranga.

R: And Muranga yeah.

**I: And Muranga?**

R2: Kiambu and Muranga.

**I: That is where your patients mostly come?**

R2: Those are catchment areas.

**I: Those are catchment areas?**

R2: Mmhh.

**I: The common orthopaedic cases you refer, we have talked about.**

R2: Mmhh.

**I: The pelvic fractures, spinal, degloving injuries involving soft tissue cover; need soft tissue cover or need plastic review.**

R2: Mmhh.

**I: Mostly?**

R2: Mmhh.

**I: For kids, anything special about children that you refer?**

R2: For peads mmm…Doctor have you seen any paediatric cases, very rare?

R: Umm, for kids what we mostly get is fracture femur; fracture femur Peads but we don’t have a lot of peads patients.

**I: So, you have few peads…**

R2: Cases

**I; Peads cases in orthopaedics.**

R2: Yeah.

**I: So, rarely do you refer peads cases?**

R: Yeah, rarely because of the numbers that come in, therefore…

**I: Less referrals due to low numbers.**

R: Yeah.

**I: What are the other reasons that is linked to the referrals? What are the factors associated, what are the reasons that referrals… Apart from those complex injuries you have talked about, what are other reasons that patients come from Thika to Kenyatta; in particular Kenyatta, not any other place.**

R2: Sometimes, what I can say is about…Sometimes I don’t think the…I guess sometimes, but I don’t think the financial factor is…the current financial factor is not really there

R: [crosstalk 11: 53]

**I: What is the main factor?**

R: The medical care and the nature of the patient.

R2: The nature of the patient.

I: Personnel issues;

R: nature of the cases.

R2: Nature of the cases, because personnel is there

R: The only thing most likely is the implants.

R2: The implants…

**I: Lack of implants?**

R2: Mmhh.

**I: Like which implants?**

R: Right now we don’t have [Inaudible 12:18]

**I: Which implants are lacking now?**

R2: All implants; at the moment all implants.

R: Plates.

**I: Even plates they have to buy?**

R: Yes, because there is none

R: Nails

**I: They are not there?**

R: They are not there right now.

**I: So patients have to buy?**

R2: Those ones are…

R: We don’t have them. We refer them.

R2: We have to refer them now.

R: The hospital is supposed to supply…

R2: Supposed to supply, but there is none at the moment.

**I: So, mainly it’s the implant?**

R2: The implant, yeah.

**I: Which other reasons do you refer to KNH in particular? This means even the long bones you refer because there is no nails.**

R2: At the moment, we are talking about at the moment now

**I: But before?**

R2: Before no

**I: What was the main reason before?**

R2: But still there is the issue about implants

**I; It has been there for a long time?**

R: Like now when we get the complex…

**I: Last year?**

R: Yes, like now when we are talking about the complex pelvic injuries, we don’t have them…

**I; But do you have people who can do pelvic fractures surgery here?**

R: We do, they can do yes.

**I: So this lack of implants is actually not for plates, but this lack of implant is for pelvic injuries?**

R: Most pelvic. Then again still on the issue of degloving injury that needs…But we talked about that; these other factors.

**I; So, lack of plastic surgeon?**

2: Mmhh

**I: Because you have no plastic surgeon, you end up referring. That’s a considered issue right?**

R; Yes.

**I: What other reasons do you refer?**

R: Those are the main reasons.

**I: What of patient preference, is there a patient that says “No, I want to go to Kenyatta”?**

R2: No, very rare.

**I: Not so much.**

R2: Not so much

**I: Pardon?**

R2: Not so much

**I: So, patient preference is not an issue?**

R2: It’s not an issue.

**I: It’s not an issue. So the main is just complex injuries. We have complex injuries where you don’t have the implants available but also sometimes there could be injuries where the plastic surgeon is needed and there are no plastic surgeons here.**

R: Mmhh.

**I: Those are the only reasons why you refer?**

R: Yeah, those are the main reasons.

**I; With fracture femur those ones you don’t refer?**

R: No, those ones we do here.

**I: Even last year you didn’t refer?**

R2: Even not last year, even the following year, I thinks it’s like…Okay [inaudible 15:09], but all these other months, everything has been running smoothly.

**I: So, usually most of the times if you have a long bone, it is an open wound that has made you to refer the patient?**

R2: yeah, [inaudible 15:25]

R: Even the [inaudible 15: 29] it reduced, and then [inaudible 15:35] [background noise] we have referred cases with open fractures.

**I; You refer the ones with a flap?**

R: Yes.

**I: You refer those ones that you think will need a flap?**

R: Yes.

**I: Any recommendations you would like regarding referral of cases to Kenyatta? Either the process or the cases, what would you need to reduce the way to refer, or to improve on the referral? Or challenges that you are facing; any challenges you are facing in orthopaedic management that you think of?**

R: Management and referral?

**I: Both management and referral.**

R: Probably orthopaedic beds.

**I; Orthopaedic beds?**

R: Yes.

**I: What is wrong with orthopaedic beds?**

R: [inaudible 16:45] regular so that…

**I: You need it for**

R: Option.

**I: They just make normal beds?**

R: Mmhh.

**I: And then you improvise the contraction period?**

R: Mmhh.

**I: Anything else? [Interruption]**

R2: [Inaudible 17:12] within the [inaudible 17: 19] the process should be easy.

**I: Process of?**

R2: The referrals; when you make those calls, by the time you are making that call, you will have a CS that says this patient needs to…Most of the CS in KNH. But again we are told first do all these investigations, do this and this and the patient is more deteriorating, again will not feel that…

**I: So [inaudible 17:53] management and patient deterioration.**

R2: Mmhh

**I; Anything else that you may think of?**

R: Finances

**I: Finances should be a factor.**

R: Because most of these patients don’t have NHIF

**I: Patients have no NHIF?**

R: Mmhh.

**I: So, how does that affect the management of orthopaedic cases?**

R: Getting implants, they have to pay in cash, sometimes there are challenges they don’t have insurance.

**I: Now they have to pay…**

R: Mmhh.

R2: Some patients will have to stay with their fractures

**I: Or you refer them**

R2: Even if you refer them even to that KNH, they will not adhere because even the patient will be reluctant to go there. Because, even if they go there “I will still go and pay”, and you don’t have that cash. So, it becomes an issue.

R: They stay for a long time with that fracture

**I: Anything you would like to say, I think that has been useful. I thought actually long bones cases that you also refer, and you are saying long bones are cases that you don’t refer, and it came up.**

R2: No, that is something that is done here.

**I: Oh, those are done here?**

R2: Yeah. 3 or 4in a day

**I: In a day?**

R2: Yeah.

**I; Every day?**

R2: No, we have three theatre days, so at least its surgeon does three in cases, each three so at least…

**I; You cover…**

R2: At least we would have coved the workload

**I: So, you have 3 surgeons?**

R2: We have 4.

**I: 4 orthopaedic?**

R2: Yes.

**I: You have 4 orthopaedic surgeons?**

R2: We have Faraj, Mwaura, Muringwa and Chege.

**I: You have 4 orthopaedic surgeons**

R: Muringwa does general

R2: let’s say like 3, 3 will be more…

R: Accurate.

R2: More accurate.

**I: So 3 surgeons who do orthopaedic?**

R2: Mmhh.

**I: You are very well equipped, isn’t it?**

R2: Mmhh.

**I: What is missing is the plastic that is what you are trying to say?**

R2: Mmhh.

**I: Once you have plastic it will reduce?**

R2: Yes.

**I; But also, one of the things we talked about is also spine. So lack of personnel there is not only for plastic, but is also for spine.**

R2: Yeah.

**I: Right?**

R2: Mmhh.

**I: So spine surgeon.**

R: I understand that Chege is a spine surgeon.

R2: Chege, yeah.

**I: Does he do spine**

R2: He’s a specialist in that. I think again he feels that maybe the hospital may lack some equipment, so…

**I: So spine surgeon you have**

R: I think we will have to confirm.

R2: We will have to confirm first.

**I: That is the spine surgeon and then the of course…The spine surgeon and of course implants for spine fractures are lacking. Isn’t it?**

R: Mmhh.

**I; Because of that you cannot do.**

R: Mmhh.

**I; But you also need a proper**

R: Mmhh.

**I; Those are the main things I have seen you have talked about; that your refer pelvic and spinal and both of them is because of implants related, but also partly because of personnel.**

R: Mmhh.

**I: If you get complex spine or complex pelvic, and obviously somebody who is specialized in [inaudible 22:00]**

R: Mmhh.

**I: So, those are the gaps that exist?**

R: Mmhh.

**I; Good. I think that has been useful, anything else you would wish to talk… I think that is the thing that I wanted to capture; I think we have addressed most of them.**

R: Most of them, yeah.

**I: Issue of space, it’s not an issue?**

R2: No, it’s not an issue.

**I: You have no issue with space, you have enough bed space; those are not challenges?**

R: Mmhh.

**I: Okay, I think those are the only things I wanted to discuss, unless you have any further comment.**

R: No comment.

**I: I think the essence for me was if I can understand the reason why most of the patients are still coming from…and going…But you have explained very well. So I think those are much issues.**
